# Epigenome-wide meta-analysis of blood DNA methylation in newborns and children identifies numerous loci related to gestational age

**DOI:** 10.1186/s13073-020-0716-9

**Published:** 2020-03-02

**Authors:** Simon Kebede Merid, Alexei Novoloaca, Gemma C. Sharp, Leanne K. Küpers, Alvin T. Kho, Ritu Roy, Lu Gao, Isabella Annesi-Maesano, Pooja Jain, Michelle Plusquin, Manolis Kogevinas, Catherine Allard, Florianne O. Vehmeijer, Nabila Kazmi, Lucas A. Salas, Faisal I. Rezwan, Hongmei Zhang, Sylvain Sebert, Darina Czamara, Sheryl L. Rifas-Shiman, Phillip E. Melton, Debbie A. Lawlor, Göran Pershagen, Carrie V. Breton, Karen Huen, Nour Baiz, Luigi Gagliardi, Tim S. Nawrot, Eva Corpeleijn, Patrice Perron, Liesbeth Duijts, Ellen Aagaard Nohr, Mariona Bustamante, Susan L. Ewart, Wilfried Karmaus, Shanshan Zhao, Christian M. Page, Zdenko Herceg, Marjo-Riitta Jarvelin, Jari Lahti, Andrea A. Baccarelli, Denise Anderson, Priyadarshini Kachroo, Caroline L. Relton, Anna Bergström, Brenda Eskenazi, Munawar Hussain Soomro, Paolo Vineis, Harold Snieder, Luigi Bouchard, Vincent W. Jaddoe, Thorkild I. A. Sørensen, Martine Vrijheid, S. Hasan Arshad, John W. Holloway, Siri E. Håberg, Per Magnus, Terence Dwyer, Elisabeth B. Binder, Dawn L. DeMeo, Judith M. Vonk, John Newnham, Kelan G. Tantisira, Inger Kull, Joseph L. Wiemels, Barbara Heude, Jordi Sunyer, Wenche Nystad, Monica C. Munthe-Kaas, Katri Räikkönen, Emily Oken, Rae-Chi Huang, Scott T. Weiss, Josep Maria Antó, Jean Bousquet, Ashish Kumar, Cilla Söderhäll, Catarina Almqvist, Andres Cardenas, Olena Gruzieva, Cheng-Jian Xu, Sarah E. Reese, Juha Kere, Petter Brodin, Olivia Solomon, Matthias Wielscher, Nina Holland, Akram Ghantous, Marie-France Hivert, Janine F. Felix, Gerard H. Koppelman, Stephanie J. London, Erik Melén

**Affiliations:** 1 Institute of Environmental Medicine, Karolinska Institutet, Stockholm, Sweden; 2 Department of Clinical Sciences and Education, Södersjukhuset, Karolinska Institutet, Stockholm, Sweden; 3 Epigenetics Group, International Agency for Research on Cancer, Lyon, France; 4 MRC Integrative Epidemiology Unit, University of Bristol, Bristol, UK; 5 Population Health Sciences, Bristol Medical School, University of Bristol, Bristol, UK; 6 Division of Human Nutrition and Health, Wageningen University & Research, Wageningen, the Netherlands; 7 Department of Epidemiology, University of Groningen, University Medical Center Groningen, Groningen, The Netherlands; 8 Computational Health Informatics Program, Boston Children’s Hospital and Harvard Medical School, Boston, MA USA; 9 Computational Biology And Informatics, University of California, San Francisco, San Francisco, CA USA; 10 HDF Comprehensive Cancer Center, University of California, San Francisco, San Francisco, CA USA; 11 Department of Preventive Medicine, University of Southern California, Los Angeles, USA; 12 Sorbonne Université and INSERM, Epidemiology of Allergic and Respiratory Diseases Department (EPAR), Pierre Louis Institute of Epidemiology and Public Health (IPLESP UMRS 1136), Saint-Antoine Medical School, Paris, France; 13 NIHR-Health Protection Research Unit, Respiratory Infections and Immunity, Imperial College London, London, UK; 14 Department of Epidemiology and Biostatistics, The School of Public Health, Imperial College London, London, UK; 15 Centre for Environmental Sciences, Hasselt University, Hasselt, Belgium; 16 ISGlobal, Barcelona Institute for Global Health, Barcelona, Spain; 17 Universitat Pompeu Fabra (UPF), Barcelona, Spain; 18 CIBER Epidemiología y Salud Pública (CIBERESP), Madrid, Spain; 19 IMIM (Hospital del Mar Medical Research Institute), Barcelona, Spain; 20 Centre de Recherche du Centre Hospitalier Universitaire de Sherbrooke (CHUS), Sherbrooke, QC Canada; 21 The Generation R Study Group, Erasmus MC, University Medical Center Rotterdam, Rotterdam, the Netherlands; 22 Department of Pediatrics, Erasmus MC, University Medical Center Rotterdam, Rotterdam, the Netherlands; 23 Department of Epidemiology, Geisel School of Medicine, Dartmouth College, Lebanon, USA; 24 School of Water, Energy and Environment, Cranfield University, Cranfield, Bedfordshire, MK43 0AL UK; 25 Division of Epidemiology, Biostatistics, and Environmental Health, School of Public Health, University of Memphis, Memphis, USA; 26 Center for Life Course Health Research, Faculty of Medicine, University of Oulu, Oulu, Finland; 27 Biocenter Oulu, University of Oulu, Oulu, Finland; 28 Department of Genomic of Complex diseases, School of Public Health, Imperial College London, London, UK; 29 Department of Translational Research in Psychiatry, Max-Planck-Institute of Psychiatry, Munich, Germany; 30 Division of Chronic Disease Research Across the Lifecourse (CoRAL), Department of Population Medicine, Harvard Medical School and Harvard Pilgrim Health Care Institute, Boston, MA USA; 31 School of Pharmacy and Biomedical Sciences, Faculty of Health Sciences, Curtin University, Bentley, Australia; 32 Curtin/UWA Centre for Genetic Origins of Health and Disease, School of Biomedical Sciences, Faculty of Health and Medical Sciences, University of Western Australia, Perth, Australia; 33 Bristol NIHR Biomedical Research Centre, Bristol, UK; 34 Centre for Occupational and Environmental Medicine, Stockholm, Stockholm Region Sweden; 35 Children’s Environmental Health Laboratory, University of California, Berkeley, Berkeley, CA USA; 36 Division of Neonatology and Pediatrics, Ospedale Versilia, Viareggio, AUSL Toscana Nord Ovest, Pisa, Italy; 37 Department of Public Health & Primary Care, Leuven University, Leuven, Belgium; 38 Department of Medicine, Université de Sherbrooke, Sherbrooke, Canada; 39 Research Unit for Gynaecology and Obstetrics, Department of Clinical Research, University of Southern Denmark, Odense, Denmark; 40 College of Veterinary Medicine, Michigan State University, East Lansing, MI USA; 41 Department of Health and Human Services, National Institute of Environmental Health Sciences, National Institutes of Health, RTP, Durham, NC USA; 42 Norwegian Institute of Public Health, Oslo, Norway; 43 Department of Epidemiology and Biostatistics, MRC–PHE Centre for Environment & Health, School of Public Health, Imperial College London, London, UK; 44 Unit of Primary Care, Oulu University Hospital, Oulu, Finland; 45 Department of Psychology and Logopedics, Faculty of Medicine, University of Helsinki, Helsinki, Finland; 46 Turku Institute for Advanced Studies, University of Turku, Turku, Finland; 47 Department of Environmental Health Sciences, Mailman School of Public Health, Columbia University Medical Center, New York, NY USA; 48 Telethon Kids Institute, University of Western Australia, Perth, Australia; 49 Channing Division of Network Medicine, Department of Medicine, Brigham and Women’s Hospital and Harvard Medical School, Boston, MA USA; 50 Center for Environmental Research and Children’s Health (CERCH), University of California, Berkeley, Berkeley, CA USA; 51 MRC-PHE Centre for Environment and Health, School of Public Health, Imperial College London, London, UK; 52 Department of Biochemistry, Université de Sherbrooke, Sherbrooke, QC Canada; 53 Department of medical biology, CIUSSS-SLSJ, Saguenay, QC Canada; 54 Novo Nordisk Foundation Center for Basic Metabolic Research, Section on Metabolic Genetics, Faculty of Health and Medical Sciences, University of Copenhagen, Copenhagen, Denmark; 55 Department of Public Health, Section of Epidemiology, Faculty of Health and Medical Sciences, University of Copenhagen, Copenhagen, Denmark; 56 Clinical & Experimental Sciences, Faculty of Medicine, University of Southampton, Southampton, UK; 57 The David Hide Asthma and Allergy Research Centre, Newport, Isle of Wight UK; 58 Human Development & Health, Faculty of Medicine, University of Southampton, Southampton, UK; 59 Nuffield Department of Women’s and Reproductive Health, University of Oxford, Oxford, UK; 60 Murdoch Children’s Research Institute, Australia Faculty of Medicine, Dentistry and Health Sciences, University of Melbourne, Melbourne, Australia; 61 Department of Psychiatry and Behavioral Sciences, Emory University School of Medicine, Atlanta, USA; 62 University of Groningen, University Medical Center Groningen, Groningen Research Institute for Asthma and COPD (GRIAC), Groningen, The Netherlands; 63 Faculty of Health and Medical Sciences, UWA Medical School, University of Western Australia, Perth, Australia; 64 Sachs’ Children’s Hospital, Södersjukhuset, 118 83 Stockholm, Sweden; 65 Center for Genetic Epidemiology, University of Southern California, Los Angeles, USA; 66 INSERM, UMR1153 Epidemiology and Biostatistics Sorbonne Paris Cité Center (CRESS), Research Team on Early life Origins of Health (EarOH), Paris Descartes University, Paris, France; 67 Department of Pediatric Oncology and Hematology, Oslo University Hospital, Oslo, Norway; 68 University Hospital, Montpellier, France; 69 Department of Dermatology, Charité, Berlin, Germany; 70 University of Basel, Basel, Switzerland; 71 Swiss Tropical and Public Health Institute, Basel, Switzerland; 72 Department of Women’s and Children’s Health, Karolinska Institutet, Stockholm, Sweden; 73 Department of Medical Epidemiology and Biostatistics, Karolinska Institutet, Stockholm, Sweden; 74 Pediatric Allergy and Pulmonology Unit at Astrid Lindgren Children’s Hospital, Karolinska University Hospital, Stockholm, Sweden; 75 Division of Environmental Health Sciences, School of Public Health, University of California, Berkeley, Berkeley, CA USA; 76 University of Groningen, University Medical Center Groningen, Department of Pediatric Pulmonology and Pediatric Allergology, Beatrix Children’s Hospital, GRIAC Research Institute Groningen, Groningen, The Netherlands; 77 Department of Biosciences and Nutrition, Karolinska Institutet, Huddinge, Sweden; 78 Folkhälsa Research Institute, Helsinki, and Stem Cells and Metabolism Research Program, University of Helsinki Finland, Helsinki, Finland; 79 Department of Newborn Medicine, Karolinska University Hospital, Stockholm, Sweden; 80 Science for Life Laboratory, Stockholm, Sweden; 81 Diabetes Unit, Massachusetts General Hospital, Boston, MA USA; 82 Sachs’ Children’s Hospital, South General Hospital, Stockholm, Sweden

**Keywords:** Development, Epigenetics, Gestational age, Preterm birth, Transcriptomics

## Abstract

**Background:**

Preterm birth and shorter duration of pregnancy are associated with increased morbidity in neonatal and later life. As the epigenome is known to have an important role during fetal development, we investigated associations between gestational age and blood DNA methylation in children.

**Methods:**

We performed meta-analysis of Illumina’s HumanMethylation450-array associations between gestational age and cord blood DNA methylation in 3648 newborns from 17 cohorts without common pregnancy complications, induced delivery or caesarean section. We also explored associations of gestational age with DNA methylation measured at 4–18 years in additional pediatric cohorts. Follow-up analyses of DNA methylation and gene expression correlations were performed in cord blood. DNA methylation profiles were also explored in tissues relevant for gestational age health effects: fetal brain and lung.

**Results:**

We identified 8899 CpGs in cord blood that were associated with gestational age (range 27–42 weeks), at Bonferroni significance, *P* < 1.06 × 10^− 7^, of which 3343 were novel. These were annotated to 4966 genes. After restricting findings to at least three significant adjacent CpGs, we identified 1276 CpGs annotated to 325 genes. Results were generally consistent when analyses were restricted to term births. Cord blood findings tended not to persist into childhood and adolescence. Pathway analyses identified enrichment for biological processes critical to embryonic development. Follow-up of identified genes showed correlations between gestational age and DNA methylation levels in fetal brain and lung tissue, as well as correlation with expression levels.

**Conclusions:**

We identified numerous CpGs differentially methylated in relation to gestational age at birth that appear to reflect fetal developmental processes across tissues. These findings may contribute to understanding mechanisms linking gestational age to health effects.

## Background

Preterm birth (birth before 37 weeks’ gestation) is associated with increased neonatal morbidity and mortality [1, 2], as well as later health [3–6]. In children born at very young gestational ages, bronchopulmonary dysplasia, retinopathy and neurodevelopmental impairment are major health challenges [7–12]. Lower lung function is observed in children born moderately preterm, i.e. between 32 and 36 completed weeks, compared to those born at term [13]. Even variation in gestational age within the normal range (37–41 weeks) is related to various health outcomes, including neurological and cognitive development [14–17] and respiratory disease [4]. Mechanisms for many of these findings are not well understood.

The epigenome is known to have an important role during fetal development. The best studied epigenetic modification is methylation. DNA methylation patterns have been associated with environmental factors relevant to preterm birth, including smoking, air pollution exposure, microbial and maternal nutritional factors [18–22]. Such exposure-related epigenetic patterns potentially influence gene expression profiles and/or susceptibility to chronic disease during the lifecourse [23, 24]. Further, DNA methylation in whole blood at birth may also reflect development across fetal life. It is possible that DNA methylation changes at birth may contribute to the myriad immediate and late health outcomes that have been associated with gestational age.

Knowledge about DNA methylation and gene expression profiles associated with length of gestation may help to better understand both the molecular basis of abnormal processes related to prematurity as well as normal human development. Several studies have reported associations of gestational age among both term and preterm births with cord blood DNA methylation [25–29]. In the largest EWAS to date (*n* = 1753 newborns), 5474 CpGs in cord blood were associated with gestational age [30]. While these individual studies have identified widespread associations of DNA methylation patterns at birth with gestational age, meta-analysis of results from multiple individual cohorts increases sample size and, thus, greatly increases power to detect robust differential methylation signals.

We examined DNA methylation levels in newborns in relation to gestational age in a large-scale meta-analysis and also examined functional effects on expression of nearby genes of potential relevance for later health. We meta-analysed harmonized cohort specific EWAS results of the association of gestational age with cord blood DNA methylation levels from the Pregnancy And Childhood Epigenetics (PACE) Consortium of pregnancy and childhood cohorts [31]. We also examined associations with continuous gestational age limited to term newborns. CpGs that were differentially methylated in cord blood in relation to gestational age were then analysed in two fetal tissues (lung and brain), with relevance for health impacts of low gestational age [7–12]. We conducted analyses to explore whether associations of CpG methylation with gestational age persisted in older children aged 4–18 years. DNA methylation status at the identified CpGs was analysed for association with gene expression patterns of nearby genes in cord blood during different developmental stages. Finally, we performed pathway and functional network analysis of identified genes to gain insight into the biological implications of our findings.

## Methods

**Fig. 1:**
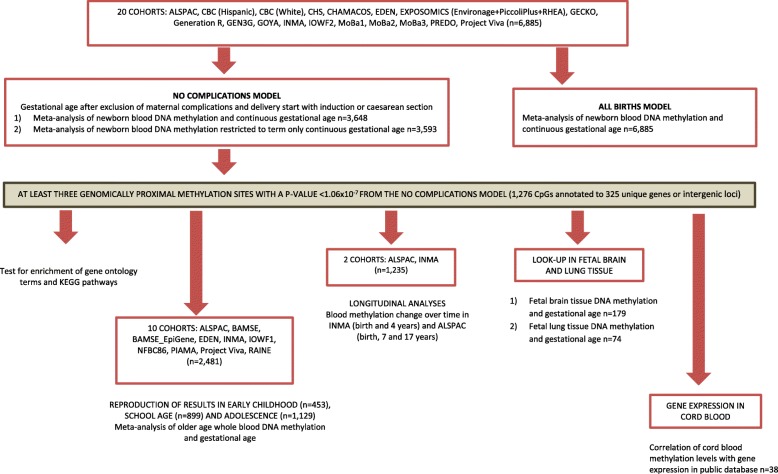
An overview of the study design

Figure 1 gives an outline of the design of this study.

### Study population

A total of 11,000 participants in 26 independent cohorts were included in our study. In the “all births model” meta-analysis, we included *n* = 6885 newborns from 20 cohorts. In our main “no complications model”, we excluded participants with maternal complications (maternal pre-eclampsia or diabetes or hypertension) and caesarean section delivery or delivery start with induction, leaving 3648 newborns from 17 cohorts for this analysis (Additional file 1: Table S1). For the additional look-up of persistent differential methylation at later ages, we used participants from 4 cohorts with whole blood DNA methylation in early childhood (4–5 years; *n* = 453), 5 cohorts with whole blood DNA methylation at school age (7–9 years; *n* = 899) and 5 cohorts with whole blood DNA methylation in adolescence (16–18 years; *n* = 1129). Detailed methods for each cohort are provided in Additional file 2: Supplementary information. All cohorts acquired ethics approval and informed consent from participants prior to data collection through local ethics committees (Additional file 2: Supplementary information).

### Gestational age

In each cohort, information on gestational age at birth was obtained from birth certificates (*n* = 725), medical records using ultrasound estimation (*n* = 1931), or last menstrual period date (*n* = 468), or combined estimate from ultrasound and last menstrual period date (*n* = 6630), or otherwise from self-administrated questionnaires (*n* = 1246). Gestational age was analysed in days. Women with a gestational age of more than 42 weeks (294 days) were excluded from all models. Additionally, multiple births were also excluded from the analysis.

### Methylation measurements and quality control

DNA methylation from newborns and older children was measured using the Illumina450K platform. Each cohort conducted their own quality control and normalization of DNA methylation data, as detailed in Additional file 1: Table S2. Cohorts corrected for batch effects in their data using surrogate variables, ComBat [32], or by including a batch covariate in their models. To reduce the impact of severe outliers in the DNA methylation data on the meta-analysis, cohorts trimmed the methylation beta values by removing, for each CpG, observations more than three times the interquartile range below the 25th percentile or above the 75th percentile [33]. Cohorts retained all CpGs that passed quality control and removed CpGs that were mapped to the X (*n* = 11,232) or Y (*n* = 416) chromosomes and control probes (*n* = 65), leaving a maximum total of 473,864 CpGs included in the meta-analysis.

### Cohort-specific statistical analyses

Each cohort performed independent EWAS according to a common, pre-specified analysis plan. Robust linear regression (rlm in the MASS R package [34]) was used to model gestational age as the exposure and DNA methylation beta values as the outcome. In the primary analysis, gestational age was used as a continuous variable excluding cohorts that had term-only infants. In secondary models, we modeled term-only children defined as a gestational age ≥ 37 weeks (≥ 259 days), but less or equal with 42 weeks. All models were adjusted for sex, maternal age (years), maternal social class (variable defined by each individual cohort; Additional file 1: Table S2), maternal smoking status (the preferred categorization was into three groups: no smoking in pregnancy, stopped smoking in early pregnancy, smoking throughout pregnancy, but a binary categorization of any versus no smoking was also acceptable), parity (the preferred categorization was into two groups: no previous children, one or more previous children), birth weight in grams, age of the child (years) included for older children, batch or surrogate variables. Optionally, cohorts could include ancestry, and/or selection covariates, if relevant to their study. We also adjusted for potential confounding by cell type using estimated cell type proportions calculated from a cord blood cell type reference panel [35] for newborn cohorts or the adult blood cell type reference panel [36] for cohorts with older children using the *estimateCellCounts* function in the *minfi* R package [37].

### Meta-analysis

We performed fixed-effects meta-analysis weighted by the inverse of the variance with METAL [38]. A shadow meta-analysis was also conducted independently by a second study group (see author contribution) and the results were compared [39] (and confirmed). All downstream analyses were conducted using R version 2.5.1 or later [40]. Multiple testing was accounted for by applying the Bonferroni correction level for 473,864 tests (*P* < 1.06 × 10^− 7^). A random effects model was performed using the METASOFT tool [41]. We explored heterogeneity between studies using the *I*^2^ statistic [42]. A priori, we defined *I*^2^ > 50% as reflecting a high level of between-study variation. In case of *I*^2^ > 50%, we replaced values with random effects estimates as these are attenuated in the face of heterogeneity and thus more conservative. To focus functional analyses and bioinformatics efforts on genes and loci that were found to be robustly associated with gestational age, we selected regions that had at least three adjacent Bonferroni significant CpGs (*P* < 1.06 × 10^− 7^) [43]. Genome-wide DNA methylation meta-analysis summary statistics corresponding to the main analysis presented in this manuscript are available at figshare (10.6084/m9.figshare.11688762.v1) [44].

### Analyses of differentially methylated regions

Differentially methylated regions (DMRs) were identified using two methods available for meta-analysis results comb-p [45] and DMRcate [46]. Input parameters used for the DMR calling in both algorithms are provided in Additional file 2: Supplementary information. Comb-p uses a one-step Šidák correction [45] and DMRcate uses an FDR correction [46] per default. The selected regions were defined based on the following criteria: the minimum number of CpGs in a region had to be 2, regional information can be combined from probes within 1000 bp and the multiple-testing corrected *P* < 0.01 (Šidák-corrected *P* < 0 .01 from comb-p and FDR < 0.01 from DMRcate).

### Analyses of embryonic DNA methylation

DNA methylation from lung tissue of 74 foetuses (estimated ages 59 to 122 days post conception [47]) were used for analyses of differentially methylated CpGs (three or more adjacent Bonferroni significant CpGs, *P* < 1.06 × 10^− 7^; *n* = 1276) from the newborn meta-analysis. A linear regression model adjusted for sex and in utero smoke exposure (IUS) was applied. A Bonferroni look-up level correction (0.05/1030; *P* < 4.85 × 10^− 5^) considered as significance threshold, followed by a comparison of the direction of effect with that in the cord blood meta-analysis. We also performed look-up analyses of selected 1276 CpGs in another organ, fetal brain tissue, from 179 foetuses collected between 23 and 184 days post-conception [48]. For these analyses, we kept the available Bonferroni correction *P* < 1.06 × 10^− 7^ as significance threshold, followed by a comparison of the direction of effect with that in the cord blood meta-analysis.

### Look-up analyses in older ages

Differentially methylated CpGs (three or more adjacent CpGs below the Bonferroni correction *P* < 1.06 × 10^− 7^; *n* = 1276) from the newborn meta-analyses were analysed with a look-up approach using data from four early childhood, five school age, and five adolescence cohorts. Cohorts included the same covariates in these analyses as in the cord blood analyses and child age. We performed fixed effects inverse variance weighted meta-analyses using METAL [38] for these three age groups. For this hypothesis-driven analysis, CpG methylation association with gestational age was considered statistically significant at nominal *P* < 0.05, followed by a comparison of the direction of effect with that in the cord blood meta-analysis.

### Longitudinal analysis

Longitudinal DNA methylation data from birth to early childhood and from birth to adolescence were analysed for the three or more adjacent Bonferroni significant 1276 CpGs found to be associated with gestational age. DNA methylation from two time points (birth and 4 years) in INMA and three time points (birth, 7 and 17 years) in ALSPAC were analysed separately. To estimate changes in DNA methylation, we applied linear mixed models with repeated measurement taking into account the within-person time effect. The models were adjusted for covariates and estimated cell count similar to cross-sectional analysis. Interaction terms between age and gestational age were included in the model to capture differences in methylation change between birth and 4 years, birth and 7 years and 7 and 17 years per day increase in gestational age at delivery, respectively. The stable CpGs that did not change significantly from birth to adolescence had no association with age (at nominal *P* < 0.05), and no interaction between gestational age and childhood age (at nominal *P* < 0.05).

### Enrichment and functional analysis

CpGs were annotated using *FDb.InfiniumMethylation.hg19* R package, with enhanced annotation for nearest genes within 10 Mb of each site, as previously described [20]. Gene Ontology (GO) and Kyoto Encyclopedia of Genes and Genomes (KEGG) pathway enrichment analyses were performed using the overrepresentation analysis (ORA) tool ConsensusPathDB (http://consensuspathdb.org/ [49, 50]). *P* values for enrichment were adjusted for multiple testing using the FDR method.

### DNA methylation in relation to gene expression

Correlations between DNA methylation and gene expression levels were tested using paired DNA methylation and gene expression data in publicly available datasets. We tested transcript levels of genes within a 500-kb region of the 1276 three adjacent CpGs (250 kb upstream and 250 kb downstream). The mRNA gene expression (Affymetrix Human Transcriptome Array 2.0) and methylation (Illumina Infinium® HumanMethylation450 BeadChip assay) were measured in cord-blood samples from 38 newborns [51–53]. First, we created residuals for mRNA expression and residuals for DNA methylation and used linear regression models to evaluate correlations between expression residuals and DNA methylation residuals. These residual models were adjusted for covariates, estimated white blood cell proportions, and technical variation. We corrected these analyses for multiple testing using Bonferroni correction.

## Results

### Study characteristics

We meta-analysed Illumina’s HumanMethylation450-array results from 17 independent cohorts with data on newborn DNA methylation status, and 10 cohorts with data on DNA methylation in older children (age 4 to 18 years), including 4 cohorts with DNA methylation data both at birth and at an older age (Fig. 1). Table 1 summarizes the characteristics of participating cohorts. A summary of methods used by each cohort is provided in Additional file 1: Tables S1 and S2. In our main “no complications” model, we excluded participants exposed to maternal pregnancy complications (maternal diabetes, hypertension or pre-eclampsia) and whose labour was induced or who were delivered by caesarean section. With continuous gestational age in the number of days as the exposure (gestational age range 186–294 days corresponding to 27–42 weeks), we analysed results from 3648 newborns and from 2481 older children. This model was selected as the main model because associations of DNA methylation with gestational age related to pregnancy complications or potentially influenced by obstetric interventions may be less reflective of normal developmental processes than newborns with spontaneous uncomplicated delivery. However, we also analysed a larger dataset of 6885 newborns from 20 independent cohorts, including pregnancies with pregnancy complications and obstetric interventions, referred to as the “all births model” (see below).

**Table 1:** Characteristics of each cohort included in the association meta-analysis between gestational age (GA) and DNA methylation in newborns and older children

Study population	Cohort	*N*	*N*, pre-term*	*N*, term	Age mean (SD)	Maternal age mean (SD)	Mean GA (days)	SD GA	Min GA	Max GA	Ethnicity
Newborn	ALSPAC** [29]	249	10	239	0	29.8 (4.6)	277	10.78	224	294	European
	CBC (Hispanic) [54]	128	10	118	0	27.3 (5.8)	273	17.70	196	294	Hispanic
	CBC (European) [54]	132	11	121	0	31.9 (5.7)	273	16.10	189	294	European
	CHS [55]	120	7	113	0	29.4 (5.6)	277	11.20	230	294	Mixed
	CHAMACOS [56]	110	11	99	0	25.3 (5.0)	272	10.66	210	294	Hispanic
	EDEN [57]	100	2	98	0	30.8 (5.0)	276	10.11	217	287	European
	EXPOSOMICS (Environage + PiccoliPlus + RHEA) [58]	252	17	235	0	30.5 (4.8)	273	10.50	217	294	European
	Generation R [59]	486	22	464	0	31.9 (4.2)	280	9.00	239	294	European
	INMA [60]	134	2	132	0	30.5 (4.1)	278	9.57	234	286	European
	IOW F2 [61]	93	2	91	0	23.2 (2.6)	278	10.95	236	294	European
	MoBa1** [30]	749	18	731	0	29.9 (4.3)	279	10.36	209	294	European
	MoBa2** [30]	460	15	445	0	30.0 (4.5)	278	10.49	209	294	European
	MoBa3 [20]	177	3	174	0	29.6 (4.4)	279	10.38	199	294	European
	PREDO [62]	308	5	303	0	33.4 (5.7)	278	11.20	186	294	European
	Project Viva [63]	150	3	147	0	33.2 (4.5)	278	10.11	216	294	European
	Meta-analysis	3648	138								
Early childhood	BAMSE [64]	145	10	135	4.3 (0.2)	31.2 (4.4)	275	16.22	187	293	European
	EDEN [64]	89	2	87	5.6 (0.1)	30.8 (5.1)	276	9.23	245	287	European
	INMA [64]	71	1	70	4.4 (0.2)	30.6 (4.3)	279	8.70	249	288	European
	PIAMA [64]	148	4	144	4.1 (0.2)	30.6 (3.6)	278	10.51	233	294	European
	Meta-analysis	453	17								
School age	ALSPAC [29]	273	12	261	7.5 (0.1)	29.9 (4.6)	277	10.99	224	294	European
	BAMSE [64]	141	10	131	8.4 (0.4)	31.4 (4.5)	276	15.96	197	293	European
	BAMSE_EpiGene [64]	232	8	224	8.3 (0.5)	30.8 (4.4)	278	11.47	209	294	European
	PIAMA [64]	134	3	131	8.1 (0.3)	30.5 (3.6)	278	10.61	233	294	European
	Project Viva [63]	119	2	117	7.8 (0.7)	33.5 (4.4)	278	10.32	216	294	European
	Meta-analysis	899	35								
Adolescence	ALSPAC [29]	272	13	259	17.2 (1.0)	29.9 (4.6)	277	11.04	224	294	European
	BAMSE [64]	159	7	152	16.7 (0.4)	31.2 (4.4)	278	12.70	187	294	European
	IOW F1 [61]	97	2	95	17.1 (0.5)	27.1 (5.1)	280	9.83	238	294	European
	NFBC86 [65]	287	9	276	16.1 (0.4)	29.0 (5.1)	280	8.65	237	294	European
	RAINE [66]	314	9	305	17.0 (0.3)	29.0 (5.8)	274	11.90	196	294	European
	Meta-analysis	1129	40								

*Preterm birth categorized as GA less than 37 full weeks or 259 days and as term greater than 37 weeks or 259 days (but less than 42 full weeks). **This study was included previous EWAS of gestational age [29, 30]. Cohort details and references can be found at Additional file 2 and in Felix et al. [31]

### Associations between gestational age and newborn DNA methylation

**Fig. 2:**
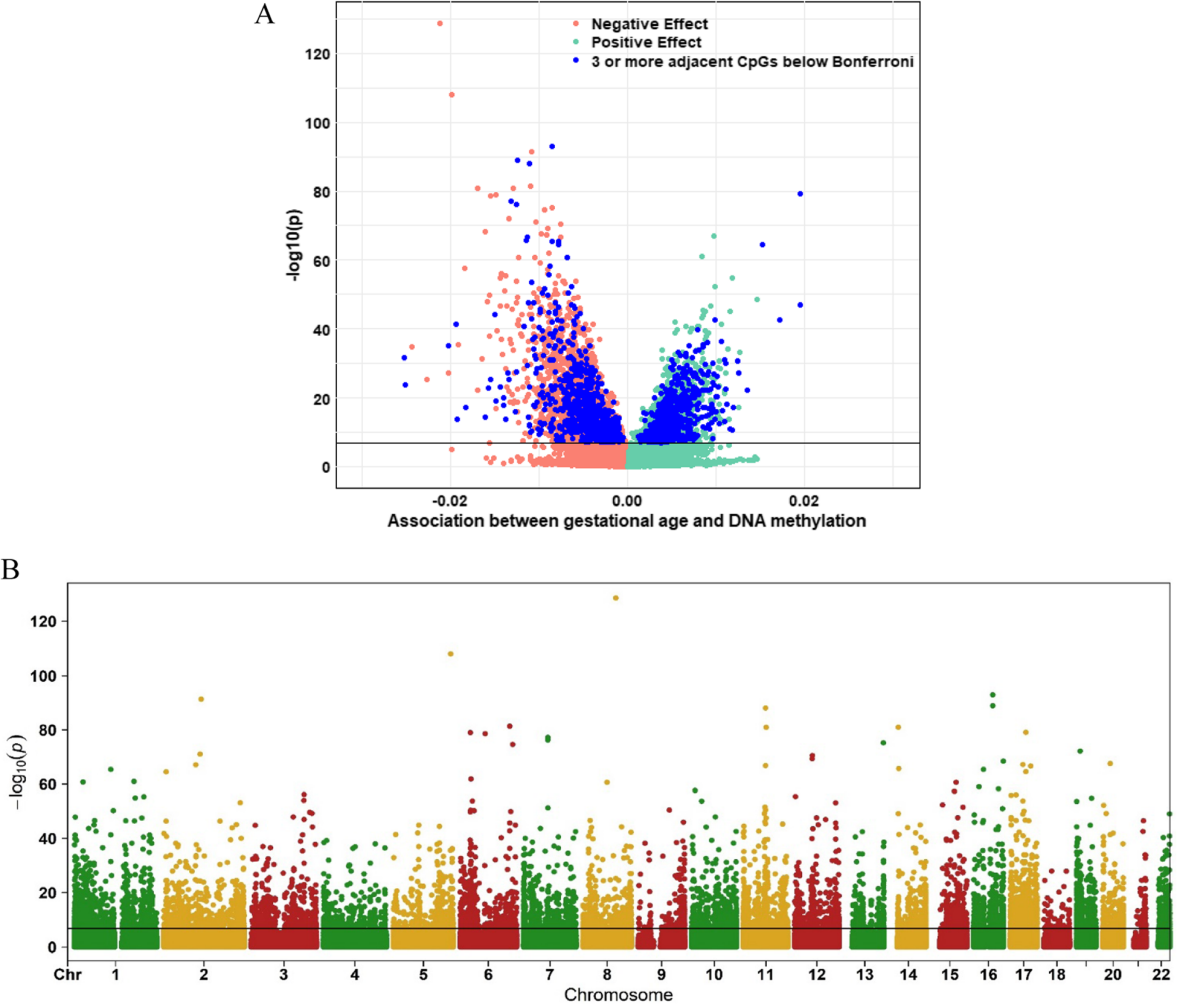
**A**, **B** Volcano (**A**) and Manhattan (**B**) plots for the meta-analysis of gestational age and offspring DNA methylation association at birth, after adjustment for covariates and estimated cell proportions. The effect size represents methylation change per gestational week

We identified 8899 CpGs in cord blood that were associated with gestational age (range 27–42 weeks), at Bonferroni significance, *P* < 1.06 × 10–7, of which 3343 were novel. These were annotated to 4966 genes. CpGs associated with gestational age had a modest predominance of negative (60%) versus positive (40%) direction of effect, with an overall absolute median difference in mean methylation of 0.36% per gestational week, IQR = [0.26%–0.49%] (Fig. 2a). In general, results were highly homogeneous; evidence of high between-study heterogeneity, using a criterion of *I*^2^ > 50%, was seen for only 319 of the 8899 CpGs (Additional file 1: Table S3). Leave one out analyses did not indicate an influential effect on meta-analysis results of any single study. However, we replaced fixed effects values with random effects estimates for those CpGs with between study *I*^2^ > 50%, as these are more conservative in the case of heterogeneity.

Differentially methylated CpGs spanned all chromosomes (Fig. 2b). The CpG with the lowest *P* value (*P* = 2.7 × 10^− 129^ for cg16103712; Table 2) was annotated to *MATN2* on chr 8, and the difference in mean methylation at this CpG was 2.13% lower per additional gestational week (equal to 0.30% per day). The CpG with the largest negative association was cg04347477, annotated to *NCOR2* on chr 12 (Table 3), with a lower mean methylation of 2.53% per additional gestational week. *B3GALT4* (chr 6) had the largest number of significant CpGs negatively associated with gestational age (21 out of 52 (40%) tested CpGs annotated to *B3GALT4*). The largest positive association was observed for cg13036381 annotated to *LOC401097* (chr 3) (Table 3) with a difference in mean methylation of 1.95% per additional gestational week. *DDR1* (chr 6) had the largest number of significant CpGs positively associated with gestational age (26/95 (27%) CpGs). A complete list of associated CpGs is presented in Additional file 1: Table S3 and the CpG variation across cohorts in Additional file 3: Figure S1 (top CpGs).

**Table 2:** The top 10 Bonferroni-significant CpGs from the meta-analysis on the association between continuous GA and offspring DNA methylation at birth adjusted for estimated cell proportions

CpGID	Chr	Genomic coordinates	Gene (Illumina annotation)	Relation to island	Distance to nearest gene	UCSC known gene	Coefficient*	*P* value	Direction of effect in each cohort**
cg16103712	8	99,023,869	*MATN2*	OpenSea	7355	*MATN2*	− 0.0030	2.70E−129	---------------
cg04685228	5	172,462,626		OpenSea	726	*ATP6V0E1*	− 0.0028	8.55E−109	------?--------
cg04276536	16	57,567,813	*CCDC102A*	N_Shelf	0	*CCDC102A*	− 0.0012	1.20E−93	------?--------
cg19744173	2	112,913,178	*FBLN7*	N_Shelf	0	*FBLN7*	− 0.0016	4.91E−92	---------------
cg27518892	16	57,566,936	*CCDC102A*	N_Shelf	0	*CCDC102A*	− 0.0018	1.29E−89	---------------
cg13924996	11	67,053,829	*ADRBK1*	S_Shore	0	*ADRBK1*	− 0.0016	8.59E−89	------?--------
cg04494800	6	149,775,853	*ZC3H12D*	N_Shore	1923	*ZC3H12D*	− 0.0016	4.52E−82	------?--------
cg27295118	14	22,902,226		OpenSea	− 500	*AK125397*	− 0.0024	1.20E−81	------?--------
cg26433582	11	68,848,232	*TPCN2*	N_Shore	917	*TPCN2*	− 0.0019	1.31E−81	------?--------
cg18183624	17	47,076,904	*IGF2BP1*	S_Shore	0	*IGF2BP1*	0.0028	8.36E−80	+++++++++++++++

*Coefficient corresponding to methylation change per additional day of gestational age

**Order of included cohorts in the meta-analysis: MoBa1, MoBa2, MoBa3, EDEN, EXPOSOMICS (Environage+PiccoliPlus+RHEA), CHS, IOWF2, Generation R, Project Viva, CBC (Hispanic), CBC (White), ALSPAC, PREDO, CHAMACOS and INMA.”?” Means that CpG was not measured in that cohort

**Table 3:** The top 10 Bonferroni-significant CpGs ranked by the magnitude of positive and negative effect (5 CpGs each) from the meta-analysis on the association between continuous GA and offspring DNA methylation at birth adjusted for estimated cell proportions

CpGID	Chr	Genomic coordinates	Gene (Illumina annotation)	Relation to island	Distance to nearest gene	UCSC known gene	Coefficient*	*P* value	Direction of effect in each cohort**
cg13036381	3	1.6E+ 08	*LOC401097*	N_Shore	− 927	*C3orf80*	0.00278	1.01E−47	+++++ − +++++++++
cg18183624	17	47,076,904	*IGF2BP1*	S_Shore	0	*IGF2BP1*	0.00277	8.36E−80	+++++++++++++++
cg04213841	13	49,792,685	*NA*	N_Shore	− 1788	*MLNR*	0.00245	3.60E−43	+++++?+++++++++
cg07738730	17	47,077,165	*IGF2BP1*	S_Shore	0	*IGF2BP1*	0.00217	2.87E−65	+++++++++++++ − +
cg09476997	16	2,087,932	*SLC9A3R2*	N_Shore	0	*SLC9A3R2*	0.00208	2.41E−49	+++++++++++++++
cg04347477	12	1.25E+ 08	*NCOR2*	Island	833	*NCOR2*	−0.00361	3.38E−32	---------------
cg08943494	11	36,422,615	*PRR5L*	OpenSea	69	*PRR5L*	−0.00360	1.95E−24	---------------
cg20334115	1	2.26E+ 08	*PYCR2*	N_Shelf	0	*PYCR2*	−0.00350	1.40E−35	---------------
cg16725984	16	89,735,184	*C16orf55*	Island	0	*C16orf55*	−0.00325	3.70E−26	---------------
cg16103712	8	99,023,869	*MATN2*	OpenSea	7355	*MATN2*	−0.00304	2.70E−129	---------------

*Coefficient corresponding to methylation change per additional day of gestational age

**Order of included cohorts in the meta-analysis: MoBa1, MoBa2, MoBa3, EDEN, EXPOSOMICS (Environage+PiccoliPlus+RHEA), CHS, IOWF2, Generation R, Project Viva, CBC (Hispanic), CBC (White), ALSPAC, PREDO, CHAMACOS and INMA.”?” Means that CpG was not measured in that cohort

We performed a sensitivity analysis by excluding cohorts that were included in previous EWAS of gestational age [29, 30] (three cohorts: MoBa1, MoBa2 and ALSPAC) in order to evaluate associations not driven by previous results, and found a high correlation (*r* = 0.89) of effect estimates (Additional file 3: Figure S2) compared with results from all cohorts included in the no complication model.

Next, we performed a meta-analysis of the larger dataset of 6885 participants from 20 studies without excluding maternal complications and caesarean section delivery or induced delivery. In this “all births model”, 17,095 CpGs located in or near 7931 genes were associated with gestational age after Bonferroni correction (*P* < 1.06 × 10^− 7^). Not surprisingly given the higher levels of statistical significance in this much larger data set, we found somewhat more between-study heterogeneity than in the no complications model, but high levels (*I*^2^ > 50%) were observed for only 1784 out of these 17,095 CpGs (Additional file 1: Table S4). We also observed a considerable overlap of CpGs between the two models with 93% of the 8899 CpGs in the no complication model also reaching Bonferroni significance in the all birth model and showing the same direction of effect.

### CpG localization and regulatory region analyses

**Fig. 3:**
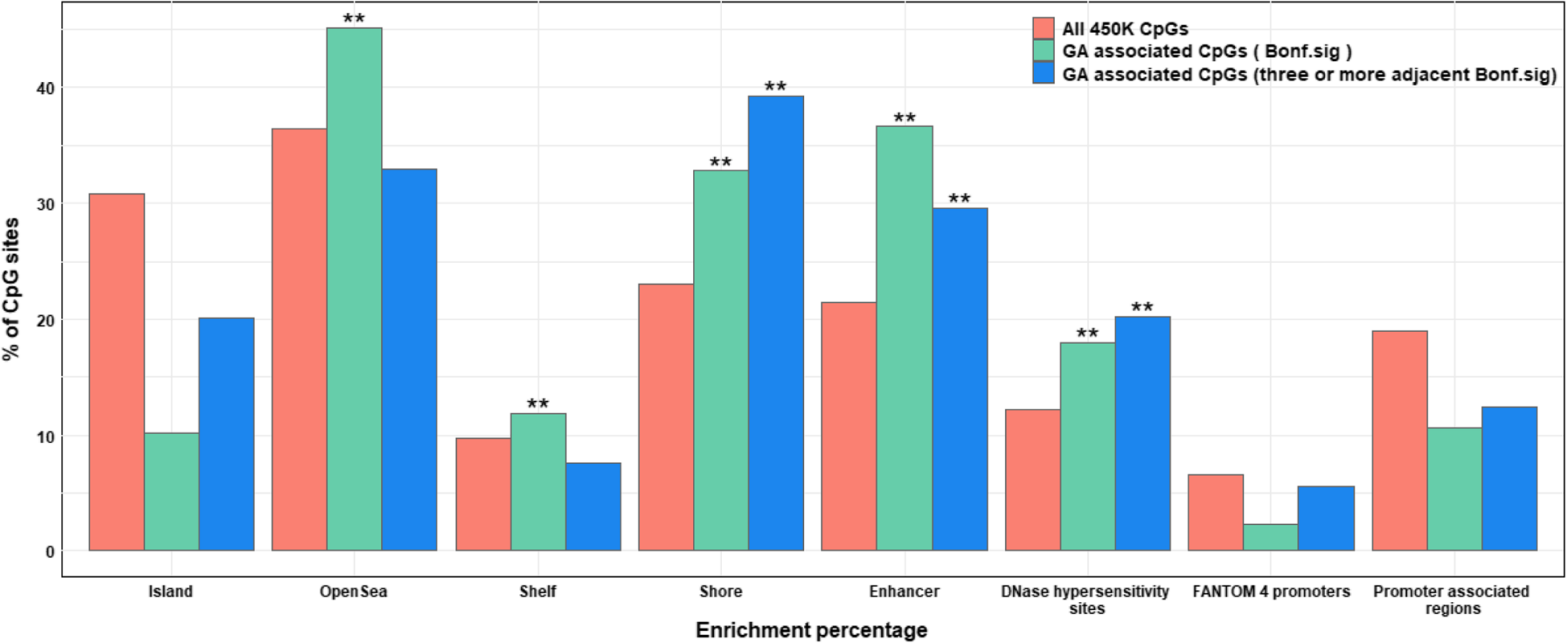
Position enrichment analyses for CpGs. Salmon: all CpGs in the Illumina450k annotation file, green: CpGs significantly associated with GA after Bonferroni correction (*P* < 1.06 × 10^− 7^) and blue: three or more adjacent CpGs associated with GA after Bonferroni correction (*P* < 1.06 × 10^− 7^). “**” represent significant two-sided doubling mid *P* value of the hypergeometric test

The 8899 differentially methylated CpGs in relation to continuous gestational age in the no complications model were enriched for localization to CpG island shores (33% of the 8899 CpGs are in shores, whereas 23% of all CpGs on the 450 K array are in shores, *P*_enrichment_ = 4.1× 10^− 100^, Fig. 3), open sea (45% versus 37%, *P*_enrichment_ = 1.4 × 10^− 63^), enhancers (37% versus 22%, *P*_enrichment_ = 1.05 × 10^− 236^), DNase hypersensitivity sites (18% versus 12%, *P*_enrichment_ = 1.3× 10^− 56^) and CpG island shelves (12% versus 10%, *P*_enrichment_ = 1.2 × 10^− 11^) (Fig. 3). In contrast, we found relative depletion in CpG islands (10% versus 31%, *P*_enrichment_ = 2.2 × 10^− 308^), FANTOM 4 promoters (2.3% versus 6.7%, *P*_enrichment_ = 6.7 × 10^− 79^) and promoter-associated regions (11% versus 19%, *P*_enrichment_ = 2.2 × 10^− 104^).

### Analysis restricted to term-births

**Fig. 4:**
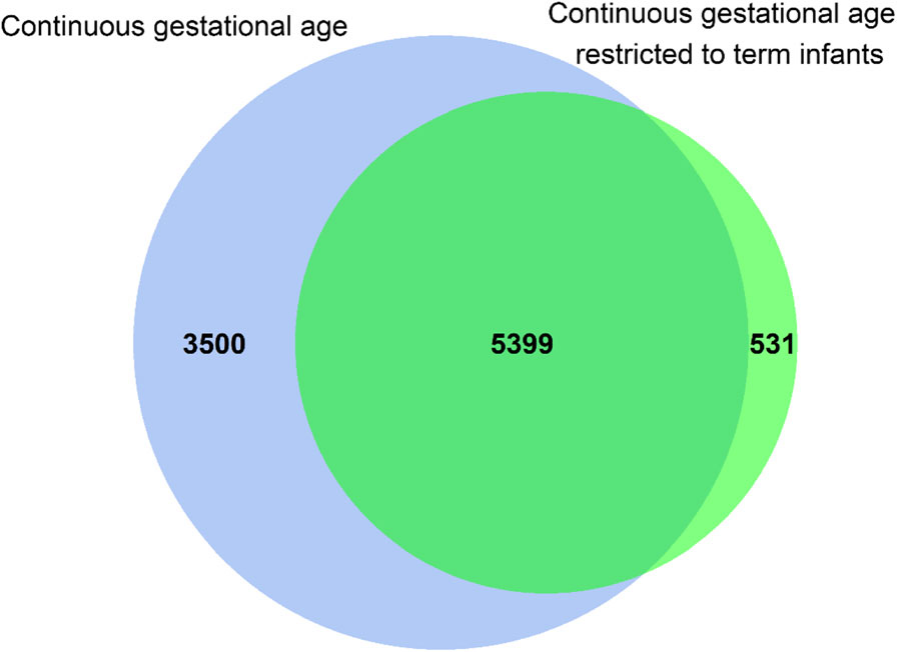
Overlap between Bonferroni-significant CpG sites from two different analyses after exclusion of maternal and delivery start with induction or caesarean section (“no complication” model). The blue colour represents the continuous gestational age main model, and the green represents the continuous model restricted to term only. Overlap of findings alters the colour

To evaluate whether observed DNA methylation differences in relation to continuous gestational age were driven by preterm birth, we repeated the no complication model including only infants born at term (gestational age 37 to 42 weeks). In this analysis, we meta-analysed results from 18 cohorts (one additional cohort with term-birth data only was included; GEN3G) (*n* = 3593). We identified 5930 sites significantly associated with gestational age at Bonferroni correction (*P* < 1.06 × 10^− 7^, median difference in mean methylation per additional gestational week = 0.43%, IQR = [0.32%–0.58%]). The vast majority (5399; 91%) of these differentially methylated CpGs overlapped with those found in the main analyses (no complications model) without exclusion of those born preterm (Fig. 4).

### Selection of CpGs for downstream analyses

Given the large number of significant associations in our main model (8899 CpGs), we focused subsequent analyses on loci including at least three adjacent CpGs that survived Bonferroni correction [43]. There were 1276 differentially methylated CpGs in 325 unique genes that fulfilled this criterion (Additional file 1: Table S5). As in the overall data, we observed a slight predominance of negative (*n* = 702; 55%) versus positive (*n* = 574; 45%) directions of effect (Fig. 2a). The lowest *P* value, *P* = 1.2 × 10^− 93^, was observed for cg04276536 (*CCDC102A,* chromosome 16). As for the full EWAS results, the largest negative and positive association effect sizes were observed for cg04347477 (*NCOR2*) and cg13036381 (*LOC401097*), respectively. These 1276 CpGs had the same CpG localization enrichment pattern as the full set of Bonferroni-significant CpGs (*n* = 8899), except that there was a relative depletion in CpG island shelves (7.6% versus 10% overall, *P*_enrichment_ = 2.3 × 10^− 12^) and open sea (32% versus 37%, *P*_enrichment_ = 2.4 × 10^− 12^) (Fig. 3).

### Differentially methylated region (DMR) analyses

Using two different methods for DMR analysis of gestational age in relation to newborn DNA methylation, we identified 4479 significant (Šidák-corrected *P* < 0.01) DMRs from the comb-p method and 14,671 significant (FDR *P* < 0.01) DMRs from DMRcate, respectively, including 2375 DMRs (representing 11,861 CpGs) that were significant based on both approaches (Additional file 1: Table S6). Out of the 8899 Bonferroni significant single CpGs, 2289 CpGs overlapped with CpGs in identified in the combined DMR analyses (11,861 CpGs). Moreover, from loci included by the three or more adjacent CpG selection (*n* = 1276), 521 CpGs overlapped with those identified in the combined DMR analyses. Of note, out of the 1276 CpGs, 1223 and 1231 CpGs were captured by DMRs identified using the comb-p and DMRcate independent approaches, respectively.

### Assessment of CpG methylation in earlier embryonic stages

We examined whether the CpGs detected in cord blood (that originate from embryonic germ layer mesoderm) were differentially methylated in relation to gestational age in other fetal tissues, lung and brain that originate from the two other embryonic germ layers, ectoderm and endoderm, respectively, collected prenatally [47, 48]. To this end, we performed look-up analyses in DNA methylation data for 74 fetal lung samples representing gestational age 59 to 122 days (~ 8 to 17 completed gestational weeks) [47]. Out of the 1276 CpGs, selected based on three or more adjacent CpGs from our no complications model, 1030 CpGs were available in the fetal lung dataset. We observed associations at Bonferroni look-up level correction significance (0.05/1030; *P* < 4.85 × 10^− 5^) between DNA methylation levels in fetal lung tissue and gestational age at tissue collection for 151 (15%) CpGs (Additional file 1: Table S7). Of these 151 (58 negatively and 93 positively associated), 78 showed the same direction of association with gestational age in cord blood and fetal lung tissue. The look-up analyses of fetal brain tissue were undertaken in 179 samples representing 23 to 184 days (~ 3 to 26 completed weeks) [48]. Out of the 1276 CpGs, we found significant associations (using Bonferroni correction *P* < 1.06 × 10^− 7^ cut-off since only this data was available for analyses; Additional file 1: Table S8) for 268 CpGs (21%) in relation to gestational age at tissue collection. Of these 268 sites, 227 had same direction of effect in the cord blood and fetal brain data. We found enrichment more than expected by chance for our cord blood gestational age associated CpGs (*n* = 1276) in fetal lung (*P* = 2.1 × 10^− 4^) and brain (*P* = 3.9 × 10^− 57^) tissue. Thirty CpGs showed significant associations with gestational age in all three tissues (cord blood, fetal lung and fetal brain).

### Assessment of CpG methylation in older children

We examined whether the differentially methylated CpGs detected in cord blood samples were associated with gestational age at birth in whole blood from older children. We conducted three separate meta-analyses (no complications model) reflecting different age periods in a total of 2481 children: (i) Early childhood (4–5 years; *n* = 453 from 4 cohorts); (ii) school age (7–9 years; *n* = 899 from 5 cohorts) and (iii) adolescence (16–18 years; *n* = 1129 from 5 cohorts), Additional file 1: Table S1. Of the 1276 three or more adjacent genome-wide significant CpGs from our analyses in cord blood, 1258 CpGs were available for analyses in all older age groups. Out of these CpGs, we observed 40 sites in early childhood, 60 sites in school age, and 60 sites in adolescence to be associated with gestational age at the nominal significance level, *P* < 0.05 with the same direction of effect (Additional file 1: Table S9). However, no CpG survived Bonferroni look-up level correction (0.05/1258; *P* < 3.97 × 10^− 5^). One CpG (cg26385222 annotated to *TMEM176B*) previously associated with gestational age at birth [27] was nominally significant in all age groups with same direction of effect.

### Longitudinal analysis

**Fig. 5:**
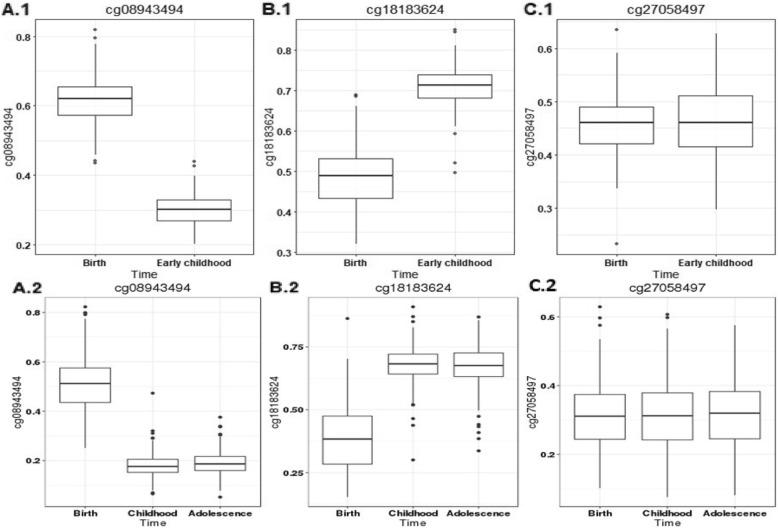
Change in DNA methylation during childhood and adolescence for selected CpG sites associated with gestational age. **A** Decreasing methylation levels from birth to childhood (**A.1**) and stabilization during adolescence (**A.2**). **B** Increasing methylation levels from birth to childhood and stabilization during adolescence. **C** Stable CpGs that did not change during childhood or adolescence; (1) INMA from birth to early childhood and (2) ALSPAC from birth to adolescence. The figures show representative single CpGs for each category (**A–C**)

The results of the longitudinal analyses of blood DNA methylation in the INMA Study (*n* = 177 with paired samples from birth and 4 years) and the ALSPAC Study (*n* = 281 with samples collected at birth, 7 and 17 years) are provided in Additional file 1: Table S10. The vast majority of gestational age associated CpGs (*n* = 1054/1276; 83%) underwent changes in methylation levels with age. Both increasing and decreasing patterns of change during early childhood (4 years) were observed, followed by stabilization during school age (7 years). For example, for cg08943494 in *PRR5L* on chr 11, an initial level of 61.5% and 51.4% in cord blood DNA methylation in INMA and ALSPAC respectively, decreased by 8.2% per year on average during early childhood in INMA and by 3.3% per year on average up to school age in ALSPAC, but then negligible further changes were seen from 7 to 17 years (Fig. 5A). In contrast, increasing levels were seen for cg18183624 (chr 17; *IGF2BP1*), from an initial 48.8% and 38.7% in cord blood DNA methylation in INMA and ALSPAC, respectively, with a 5.1% per year on average between birth to 4 years in INMA and 1.9% per year on average between birth to 7 years, but after that no changes from 7 to 17 years. (Fig. 5B).

Of the 1054 CpGs displaying changes in DNA methylation levels with age, there were 589 CpGs where gestational age was associated with changes in DNA methylation levels (i.e. where an interaction between gestational age and age was found) from birth to 4 years (INMA) and 460 CpGs with changes from birth to 7 years (ALSPAC). However, only 30 of the 1054 CpGs changed significantly in DNA methylation between 7 and 17 years (ALSPAC), suggesting that gestational age-related changes in DNA methylation levels had largely stabilized by age 7.

We identified 222 stable CpGs out of 1276 (17%) that did not change appreciably from birth to adolescence. As an example, the stable DNA methylation at cg27058497 (*RUNX3*, chromosome 1) is shown in Fig. 5C. A much lower proportion of the gestational age associated CpGs were stable from birth to adolescence compared to all CpGs on the array (17% versus 71%, *P*_enrichment_ = 2.23× 10^− 308^).

### Enrichment for biological processes and pathways

Using the complete list of 8899 CpGs annotated to 4966 genes, these were enriched for 1784 GO terms including regulation of cellular and biological processes, system development, different signaling pathways and organ development (Additional file 1: Table S11). Kyoto Encyclopedia of Genes and Genomes (KEGG) pathway analyses revealed 124 significant terms at FDR < 0.05 representing a variety of human diseases, most notably various cancers, viral infections, metabolic processes and immune-related disorders (Additional file 1: Table S12). The 325 genes annotated to the 1276 CpGs, selected by virtue of three or more CpGs being localized to the same gene, were enriched for 198 Gene Ontology (GO) terms very similar to those identified using Bonferroni significant CpGs (Additional file 1: Table S13). When restricting analyses to the 222 longitudinally stable CpGs, corresponding to 139 genes, 13 significant KEGG terms were revealed, primarily representing infection- and immune-related disorders (Additional file 1: Table S14). For 186 genes annotated to the 1054 CpGs changing with postnatal age, only one KEGG terms were identified as statistically significant (*P* = 1.2 × 10^− 3^ for the term MAPK signaling pathways; Additional file 1: Table S14).

### Correlation of DNA methylation and gene expression

For the 1276 CpGs differentially methylated in relation to gestational age with at least 3 adjacent CpGs, we assessed correlations between DNA methylation and gene expression (*cis*-eQTMs). From a publicly available dataset of expression and DNA methylation measured in 38 cord blood samples [51–53], 1174 out of the 1276 CpGs were located within a 500-kb (+/− 250 kb) window of a transcript cluster. Of these 1174, 246 unique CpGs (367 total CpG-transcript associations) correlated significantly with gene expression (Bonferroni *P* < 0.05, Additional file 1: Table S15). Forty-six percent of these DNA methylation-expression correlations were negative, with the lowest *P* = 3.55 × 10^− 6^ coeff = − 6.03 for cg01332054 and *SEMA7A* expression and the largest negative effect estimate (− 12.69) for cg26179948 and *JAZF1* expression (Additional file 3: Figure S3 A, B). Fifty-four percent were positive, with the lowest *P* = 1.04 × 10^− 5^ coeff = 2.88 for cg20139800 and *MOG* expression and the largest positive effect estimate (19.35) for cg03665259 and *CDSN* expression (Additional file 3: Figure S3 C, D).

## Discussion

In this large consortium-based meta-analysis, we identified 8899 sites across the genome where gestational age at birth was associated with cord blood DNA methylation. We also identified numerous unique differentially methylated regions (DMRs) associated with gestational age by applying two independent methods. The results were consistent when restricted to births at term, demonstrating that the majority of our results were not driven by preterm births. We confirmed many of the findings from previously published EWAS of gestational age [23, 26, 27, 29, 30, 67] and found a very high correlation between the significant CpG point estimates in previously published datasets compared to our study (e.g. corr = 0.92 between Hannon et al. CpGs and our data; Additional file 1: Table S16), but importantly, we also found 3343 CpGs corresponding to 2577 genes that had not been described previously. There was a general lack of stability of the cord blood findings into childhood and adolescence. However, there was a significant overlap of differentially methylated CpGs in cord blood, fetal brain and lung tissues.

We found that various functional elements were enriched among gestational age-associated CpGs. CpG island shores, enhancers and DNase I hypersensitive sites were particularly susceptible to DNA methylation changes in relation to gestational age, suggesting that these differentially methylated sites are of functional importance [68].

We found clear overlap of differentially methylated CpGs in cord blood, fetal brain and fetal lung tissues in relation to gestational age. Thus, our cord blood findings seem to partly capture the epigenomic plasticity of prenatal development across tissues. The gene with the largest negative magnitude of association with cord blood DNA methylation in relation to gestational age, *NCOR2*, was also differentially methylated in brain and lung fetal tissues. *NCOR2* is involved in vitamin A metabolism and has previously been associated in GWAS with lung function [69]. Vitamin A supplementation is suggested to reduce the risk of bronchopulmonary dysplasia in extremely preterm-born children [70]. Differential methylation of *NCOR2* in neurons associated with ageing has been reported [71]. The gene with the second largest magnitude of negative association with methylation at birth, *PRR5L*, has been linked in GWAS to allergic diseases, found downregulated (expression) in osteoarthritis, and differentially methylated in type II diabetes [72–74]. The gene with the lowest *P* value in our EWAS, *MATN2* plays a critical role in the differentiation and maintenance of skeletal muscles, peripheral nerves, liver and skin during development and regeneration [75] and is suggested as a potential biomarker in the early stage of osteoarthritis [76].

Differentially methylated CpGs associated with gestational age in cord blood were also present in our childhood and adolescence analyses. The only CpG (cg26385222, *TMEM176B*) that was associated with gestational age at all three time points (birth, childhood and adolescence) has been associated with gestational age in cord blood in previous studies [27]. The protein encoded by *TMEM176B* has also been suggested as a potential biomarker for various cancers [77]. The low number of significant associations with gestational age at older ages with no CpG surviving multiple test correction may be partially explained by smaller sample sizes in childhood and adolescence than at birth and by the fact that many later exposures may obscure the association. However, in agreement with the cross-sectional analyses, our longitudinal analyses showed that DNA methylation at gestational age-associated CpGs typically undergoes dynamic changes during early childhood to a much higher degree than overall for CpGs on the 450K array. For the majority of these dynamics CpGs, change was most prominent during the first years of life, with many sites tending stabilize in methylation levels by school age. We also identified a subset of the CpGs differential methylated at birth (17%) which seem stable over time. For these CpGs, the early alteration of methylation levels by length of gestation was found stable postnatally across childhood and into adolescence.

In recent analyses by Xu et al, 14,150 CpGs related to childhood age were identified [78] and we found 280 overlapping with these CpGs among our 1276 CpG list. Moreover, a study by Acevedo et al. showed 794 age-modified CpGs within 3 to 60 months after birth and 57 CpGs were overlapping with our 1276 CpG list [79]. Thus, a proportion of gestational age-related CpGs are also associated with postnatal ageing. But similar to results from Simpkin et al. [80], we observed very little overlap (only 3 CpGs) with the CpGs used to derive epigenetic age by the Hannum and Horvath approach [81, 82] or the epigenetic clock for gestational age at birth (10 CpGs overlapping) [28]. It should be noted that these studies primarily used the Illumina 27K array for analyses, which makes comparison difficult.

In the functional analyses, we observed significant enrichment for several GO terms related to embryonic development, regulation of process and immune system development. The pathway analyses identified a subset of these genes linked to diseases also associated with low gestational age, for example asthma [83], inflammatory bowel disease [84], type I/II diabetes [85] and cancer (leukaemia) [86]. Importantly, genes annotated to CpGs found stable across childhood also showed enrichment for infection- and immune-related conditions. Whether cord blood DNA methylation at these CpGs affects later disease risk remains to be studied. Interestingly, differentially methylated loci in relation to asthma development have been recently identified in newborns [87]. The stable CpG cg27058497 (*RUNX3*) has been associated with in utero tobacco smoking exposure [88], childhood asthma [89], oesophagus squamous cell carcinoma [90] and chronic fatigue syndrome [91]. Despite adjustment for maternal smoking in our gestational age EWAS model, we observed overlap between all FDR hits from our gestational age EWAS with those FDR hits presented in the maternal smoking related DNA methylation [20] with an overlap of 2302/47,324 CpGs (4.9%, *P*_enrichment_ < 2.2 × 10^− 308^). This overlap likely reflects some pregnant women under reporting their smoking behaviour and the fact that smoking-related CpGs capture quantitative smoking history better than self-report [92, 93]. However, we cannot rule out the possibility that some overlapping CpGs could be involved in biologic pathways linking smoking to the well-established consequence of shorter gestational length [94]. Other potential confounders not accounted for in this study such as maternal obesity and alcohol intake may influence offspring DNA methylation although we have found in the PACE consortium that their impact on methylation [95, 96] is very modest compared with maternal smoking in pregnancy which was included in our models.

This paper aimed at identifying CpGs associated with gestational age while adjusting for birth weight. In a recent PACE paper, we found 1071 CpGs at Bonferroni significant levels association with birth weight [97]. Even after adjustment of birth weight in our gestational age EWAS, we observed overlap between the birth weight EWAS and the current gestational age EWAS for 373/1071 CpGs (34.9% *P*_enrichment_ < 2.2 × 10^− 308^). These two perinatal factors, birth weight and gestational age, may have a shared impact on DNA methylation in newborns. However, it is difficult to disentangle the effects of these correlated factors.

To further investigate a potential functional impact of our differentially methylated CpGs, we examined correlations with gene expression in cord blood. We found multiple *cis*-eQTMs among the gestational age-related CpGs where methylation was strongly correlated with gene expression in cord blood, implying that the identified CpGs may have a direct functional effect in newborns. *IGF2BP1*, known to be involved in adiposity and cardiometabolic disease risk [98], and to play an essential role in embryogenesis and carcinogenesis [99, 100], was the most significant positively differentially methylated CpG in cord blood. Low gestational age is a well-established risk factor for later cardiometabolic disease [101]. Our expression findings likely reflect relevant for health outcomes associated with low gestational age.

There are potential study limitations in our study including heterogeneity in normalization and quality control (QC) protocols since individual cohorts performed their own QC and normalization. However, one of our previous EWAS meta-analysis reported robust results comparing the non-normalized methylation and different data processing methods used across the cohorts for normalization [20]. Furthermore, between-study heterogeneity at our pre-specified threshold was observed for only a minority of differentially methylated CpGs. Cohorts collected gestational age data from medical records, birth certificates or questionnaires in two ways, either ultrasound estimates and/or according to last menstrual period (or combined estimates), which may introduce bias. However, gestational age determined by ultrasound correlates well with last menstrual period data [102]. Despite a large sample size, we had few extreme premature births included in our dataset. Interpretation of effects of DNA methylation on gene expression was done for *cis*-effects only, not *trans*-effects. Since our analyses were primarily cross-sectional, we cannot infer the temporality in the associations and we cannot assume associations are causal [103]. We recognize the possibility that the observed methylation patterns represent fetal maturity, accompanying a “normal” developmental process or determining time in utero; it was however not possible to include foetuses who did not survive pregnancy most of whom will have been delivered very early. The majority of study participants were of European ancestry, and very few cohorts were Hispanic. We were unable to explore ethnic differences in detail since that would require large sample sizes for each ethnic group. However, when analyses were restricted to European-ancestry cohorts, the results were essentially identical with correlation coefficient 0.97 (Additional file 3: Figure S4) to those with all cohorts included. Finally, we acknowledge a potential limitation by applying a filter (regions with at least three or more adjacent CpGs with a Bonferroni-corrected *P* value < 0.05) in order to capture a set of genes robustly affected by gestational age, which may have led to potentially important single CpGs not being included in the functional analyses. In addition, genes with few CpGs represented on the 450K array are likely under-represented in the downstream analyses. The strengths of our study are large sample size, the comprehensive analyses using robust statistical methods, as well as the availability of samples at multiple ages and our ability to compare our findings with those in fetal tissue datasets. To account for potential cell type effects, we adjusted our models for estimated cell counts using cord blood and adult whole blood references [35, 36]. However, we acknowledge the limitations of available blood cell type reference data sets and recognize that some of the signals we identified as effects of gestational age might reflect differences in cell type composition that we did not completely control. Larger panels that better capture cell type composition across the range of gestational age would be a useful advance. Although we present data on all available participants in our all births model, we based our study conclusions on the main no complication model results, after excluding samples related to delivery induced by medical interventions (induction and/or caesarean section) and maternal complications.

## Conclusions

We show that DNA methylation at numerous CpG sites and DMRs across the genome is associated with gestational age at birth. Our results provide a comprehensive catalogue of differential methylation in relation to this important factor, which may serve as utility to the growing community of researchers studying the developmental origins of adult disease. Identified CpGs were linked to multiple functional pathways related to human diseases and enriched for several categories of biological processes critical to fetal development. As such, many sites might capture epigenomic plasticity of fetal development across tissues. We also found that blood DNA methylation levels in identified CpGs change over time for a majority of CpGs and that levels stabilize after school age. Taken together, our findings provide new insight into epigenetics related to preterm birth and gestational age.

## Supplementary information

Additional file 1: **Table S1.** Cohort-specific results from epigenome-wide association analyses of gestational age. **Table S2.** Normalization technique and phenotype definitions used by each cohort. **Table S3.** Bonferroni-significant CpGs from the meta-analysis on the association between continuous gestational age (no complications model) and offspring DNA methylation at birth adjusted for estimated cell counts. **Table S4.** Bonferroni-significant CpGs from the meta-analysis on the association between continuous gestational age (all births model) and offspring DNA methylation at birth adjusted for estimated cell counts. **Table S5.** Gene regions that had at least three consecutive Bonferroni significant CpG sites from the continuous gestational age analyses (no complications model). **Table S6.** DMRs (*n* = 2375) for gestational age in relation to newborn methylation (no complication model) identified by using both comb-p (*P* < 0.01) and DMRcate (FDR < 0.01) methods. **Table S7.** DNA methylation analyses in fetal lung tissue using the no complication gestational age three or more consecutive CpG list. **Table S8.** DNA methylation analyses in fetal brain tissue using the no complication gestational age three or more consecutive CpG list. **Table S9.** Methylation look-up analyses in older children using the no complication gestational age three or more consecutive CpG list. **Table S10.** Longitudinal analysis of methylation levels in the INMA and ALSPAC studies using the no complication gestational age three or more consecutive CpG list. **Table S11.** Gene Ontology (GO) term enrichment analyses for bonferroni-significant CpGs from the meta-analysis (no complications model). **Table S12.** KEGG pathway analyses for bonferroni-significant CpGs from the meta-analysis (no complications model). **Table S13.** Gene Ontology (GO) term enrichment analyses for three or more CpGs being localized to the same gene. **Table S14.** KEGG pathway analyses for stable and dynamic CpGs. **Table S15.** Correlation between methylation and gene expression levels in cord blood (cis-effects). **Table S16.** The replication of bonferroni-significant CpGs from the meta-analysis (no complications model) in previous publication.

Additional file 2. Supplementary information.

Additional file 3: **Figure S1.** Forest plot for the top 10 Bonferroni-significant CpGs from the meta-analysis on the association between continuous GA and offspring DNA methylation at birth adjusted for estimated cell proportions. **Figure S2.** Sensitivity analysis: Correlation of the point estimates for the no complications model main association of DNA methylation with gestational age (y-axis representing 3648 participants from 17 cohorts) with point estimates for a meta-analysis after excluding three cohorts (MoBa1, MoBa2 and ALSPAC) that were included in a previous publication1,2 (x-axis representing 2190 participants from 14 cohorts). **Figure S3.** Correlations between methylation and gene expression levels for selected four pairs. First, we created residuals for mRNA expression and residuals for DNA methylation and used linear regression models to evaluate correlations between expression residuals and methylation residuals. These residual models were adjusted for covariates, estimated white blood cell proportions, and technical variation. **Figure S4.** Sensitivity analysis: Correlation of the point estimates for the no complications model main association of DNA methylation with gestational age (y-axis representing 3648 participants from 17 cohorts) with point estimates for a meta-analysis after excluding Non-European three cohorts (CBC, CHS and CHAMACOS) (x-axis representing 3290 participants from 14 cohorts).
