# Supplementary material for: Epigenome-wide meta-analysis of blood DNA methylation in newborns and children identifies numerous loci related to gestational age
Source: Genome Med. 2020 Mar 2;12:25. doi: 10.1186/s13073-020-0716-9 (PMC7050134; doi:10.1186/s13073-020-0716-9)
Supplement: Supplementary file 2 — Supplementary information. [file 13073_2020_716_MOESM2_ESM.docx]

Supplementary Content

[Supplementary methods 2](#_Toc30421464)

[Cohort-specific methods (alphabetical order) 3](#_Toc30421468)

[Fetal lung methods 28](#_Toc30421501)

[Cohort-specific acknowledgements (alphabetical order) 29](#_Toc30421502)

[Cohort-specific funding statements (alphabetical order) 31](#_Toc30421528)

# Supplementary methods

##

## *Study populations*

A total of n=11,000 participants in 26 independent cohorts were included in the study. The cord blood meta-analysis included n=6,885 participants from 20 cohorts and 10 cohorts with data on methylation in older children (n=4,115 mean age ranges from 4 to 18), including 4 cohorts which also contributed to the cord blood analysis.

The following 20 cohorts participated in the meta-analysis of gestational age and newborn blood DNA methylation: The Avon Longitudinal Study of Parents and Children (ALSPAC), two independent datasets from the Californian Birth Cohort (CBC_Hispanics and CBC_Caucasians), Center for the Health Assessment of Mothers and Children of Salinas (CHAMACOS), Children’s Health Study (CHS), Etude des Déterminants pré et post natals du développement et de la santé de l′Enfant (EDEN), three cohorts from the ENVIRonmental influence ON early AGEing (ENVIRONAGE); the Piccolipiù study (PICCOLIPLUS) and the mother and child cohort in Crete (RHEA) were pooled and analysed as a single cohort referred to as EXPOsOMICS, Groningen Expert Center for Kids with Obesity (GECKO), the Generation R Study (GENR), the Genetics of Glucose regulation in Gestation and Growth (GEN3G), the Genetics of Overweight Young Adults (GOYA), INfancia y Medio Ambiente (INMA), the Isle of Wight Birth Cohort third generation (IOW F2),three independent datasets from the Norwegian Mother and Child Cohort Study (MOBA1, MOBA2, MOBA3), the Prediction and Prevention of Preeclampsia and Intrauterine Growth Restriction (PREDO) and Project Viva (Viva). After excluding participants exposed to maternal pregnancy complications (maternal diabetes, hypertension or pre-eclampsia) and whose labour was induced or who were delivered by Caesarean section these three cohorts (GECKO, GEN3G and GOYA) were excluded and only 17 cohorts were participated in the meta-analysis of gestational age and newborn blood DNA methylation.

An additional four cohorts participated in the meta-analysis of gestational age and whole blood DNA methylation at early childhood (ages 4-5): the Children Allergy Milieu Stockholm Epidemiology cohort (BAMSE), Etudes des Déterminants pré et postnatals précoces du développement et de la santé de l’Enfant (EDEN), INfancia y Medio Ambiente (INMA) and the Prevention and Incidence of Asthma and Mite Allergy birth cohort (PIAMA).

At school age (ages 7-9) five cohorts participated in the meta-analysis of gestational age and whole blood DNA methylation: The Avon Longitudinal Study of Parents and Children (ALSPAC), two independent datasets from the Children Allergy Milieu Stockholm Epidemiology cohort (BAMSE, BAMSE-EPIGENE), the Prevention and Incidence of Asthma and Mite Allergy birth cohort (PIAMA) and Project Viva (Viva).

Moreover, five cohorts participated in the meta-analysis of gestational age and whole blood DNA methylation at adolescence (ages 16-18): The Avon Longitudinal Study of Parents and Children (ALSPAC), the Children Allergy Milieu Stockholm Epidemiology cohort (BAMSE), IOW birth cohort second generation (IOW F1), The Northern Finland Birth Cohort 1986 (NFBC86) and the Western Australia Pregnancy Cohort (RAINE).

***Polymorphic and cross-reactive probes***

To reduce the risk of false hits by probes that may reflect underlying genetic polymorphisms instead of methylation effects, we compared the gestational age associated 1,276 three or more adjacent CpGs below Bonferroni-corrected with two lists of cross-reactive and polymorphic probes potentially influenced by a SNP^3,4^ yielding a total of 124 CpGs as polymorphic (potentially influenced by a SNP). We assessed the influence of SNPs on the methylation levels of each of these CpGs, by performing Hartigans’ dip test^5,6^ for unimodality in distributions of methylation levels in the large MeDALL dataset^7^. However, we found no evidence for multimodality for these 124 CpGs (p > 0.05). Additional visual inspection of density plots for the 20 CpGs with the lowest dip test p-values confirmed a unimodality distribution (data not shown).

## *Assessing fetal tissues and cord blood DNA methylation*

We compared the gestational age associated three or more adjacent CpGs (Bonferroni significant) in cord blood with fetal lung^8^ (151 CpG sites) and fetal brain^9^ (268 CpG sites) data. We then explored the directions of association of these fetal tissues CpG sites with each other and compared them with the directions of association with cord blood CpGs. Finally, we performed a hypergeometric test to calculate enrichment, which tests whether the number of overlapping CpG sites were larger than would be expected by chance.

***CpG localization enrichment***

We evaluated whether the CpGs significantly associated with gestational age in the main “no complications model” were enriched, relative to all CpGs analyzed, for several biologic annotations provided in the Illumina annotation file^10^. We assessed enrichment by using the two-sided doubling mid p-value of the hypergeometric test^11^.

***Analysis of differentially methylated regions***

Input parameters used in the comb-p algorithm:

**Parameter Value Description**

**dist** 1000 Maximum distance to search for adjacent peaks.

**seed** 0.05 A value must be at least this large/small in order to seed a region.

**region-filter-p** 0.01 Maximum adjusted region-level p-value to be reported in final output.

**region-filter-n** 2 Require at least this many probes for a region to be reported in final output.

Input parameters used in the DMRcate algorithm:

**Parameter Value Description**

**lambda** 1000 Gaussian kernel bandwidth for smoothed-function estimation.

Gaps ≥ lambda between significant CpG sites will be in separate DMRs.

**C** 2 Scaling factor for bandwidth. Gaussian kernel is calculated

where lambda/C = sigma. Empirical testing shows that, for 450k data when lambda = 1000, near-optimal prediction of sequencing-derived DMRs is obtained when C is approximately 2.

**Pcutoff** 0.01 p-value cutoff to determine DMRs.

**min.cpgs** 2 Minimum number of consecutive CpGs constituting a DMR.

#

# Cohort-specific methods (alphabetical order)

## Avon Longitudinal Study of Parents and Children (ALSPAC)

***Design and study population***

ALSPAC is a large, prospective cohort study based in the South West of England. 14 541 pregnant women resident in Avon, UK with expected dates of delivery 1st April 1991 to 31st December 1992 were recruited and detailed information has been collected on these women and their offspring at regular interval^16,17^. Please note that the study website contains details of all the data that is available through a fully searchable data dictionary and variable search tool" and reference the following webpage: <http://www.bristol.ac.uk/alspac/researchers/our-data/>. As part of the ARIES (Accessible Resource for Integrated Epigenomic Studies, http://www.ariesepigenomics.org.uk/) project, the Illumina Infinium HumanMethylation450 BeadChip (Illumina Inc., San Diego, USA) has been used to generate epigenetic data on 1018 mother-offspring pairs in the ALSPAC cohort^18^. The ARIES participants were selected based on availability of DNA samples at two time points for the mother (antenatal and at follow-up when the offspring were adolescents) and three time points for the offspring (neonatal, childhood (age 7) and adolescence (age 17)). Written informed consent has been obtained for all ALSPAC participants. Ethical approval for the study was obtained from the ALSPAC Ethics and Law Committee and the Local Research Ethics Committees. Consent for biological samples has been collected in accordance with the Human Tissue Act (2004).

***Gestational age***

Gestation was calculated (in days) based on the date of the mother's last menstrual period (LMP) when the mother was certain of this (n=192), but for uncertain LMPs and conflicts with clinical assessment the ultrasound assessment was used (n=288). Where maternal report and ultrasound assessment conflicted, an experienced obstetrician reviewed clinical records and made a best estimate (n=0).

***Methylation measurements***

Cord blood and peripheral blood samples (whole blood, buffy coats or blood spots) were collected according to standard procedures, spun and frozen at -80˚C. DNA methylation analysis and data pre-processing were performed at the University of Bristol. Following extraction, DNA was bisulfite converted using the Zymo EZ DNA MethylationTM kit (Zymo, Irvine, CA). Following conversion, the genome-wide methylation status of over 485,000 CpG sites was measured using the Illumina Infinium® HumanMethylation450k BeadChip assay according to the standard protocol. The arrays were scanned using an Illumina iScan and initial quality review was assessed using GenomeStudio (version 2011.1). The level of methylation is expressed as a “Beta” value (β-value), ranging from 0 (no cytosine methylation) to 1 (complete cytosine methylation). Genotype probes on the HumanMethylation450k were compared between samples from the same individual and against SNP-chip data to identify and remove any sample mismatches. Data were normalised in minfi using the subset quantile normalization approach. We removed probes that had a detection P-value >0.05 for >5% of samples, probes on the X or Y chromosomes and SNPs (rs probes). 468622 probes remained.

***Covariates***

Data on maternal socio-economic status, smoking, pre-pregnancy body mass index and age at delivery were self-reported by questionnaire during pregnancy. Newborn sex, parity, mode of delivery, pregnancy complications and birthweight were extracted from birth records.

***Cell type correction***

For the cord blood analyses, we used the Andrews and Bakulski reference^12^ with the Houseman method using the estimateCellCounts function in the Minfi package^19^ in R to estimate the proportions of 7 blood cell types (nucleated red blood cells, CD4+ T-lymphocytes, CD8+ T-lymphocytes, NK (natural killer) cells, B-lymphocytes, monocytes and granulocytes). For the childhood and adolescent whole blood analyses, we used the Reinius reference and the Houseman method^20,21^ to estimate the proportions of 6 cell types (CD4+ T-lymphocytes, CD8+ T-lymphocytes, B-lymphocytes, monocytes, granulocytes and natural killer cells).

***Batch correction***

Ten surrogate variables were generated using the sva package in R^22^ and included in models to adjust for technical batch.

***Exclusion criteria***

## For all models we excluded n=3 infants born more than 42+0 weeks (more than 294 days) or multiple birth cases (n=0). In the no complication model, infants whose mothers experienced pre-eclampsia, hypertension or diabetes during pregnancy (n=100), or whose delivery started with induction (n=96) or emergency caesarean section (n=16), were excluded. This information was obtained from medical birth records.

## Children (Barn), Allergy, Milieu, Stockholm, Epidemiology (BAMSE)

***Design and study population***

BAMSE (Children, Allergy, Milieu, Stockholm, Epidemiology in Swedish) is a prospective population-based cohort study of children recruited at birth and followed during childhood and adolescence. Details of the study design, inclusion criteria, enrolment and data collection are described elsewhere^23^. In short, 4,089 children born between 1994 and 1996 in four municipalities of Stockholm County were enrolled. At baseline, when the infant was approximately 2 months of age, parents completed a questionnaire that assessed residential characteristics, as well as socioeconomic and lifestyle factors. When children were 1, 2, 4, 8, 12 and 16 years, the parents completed questionnaires focusing on children’s symptoms related to wheezing and allergic diseases, as well as various exposures. The survey response rates were 96%, 94%, 91%, 84%, 82% and 78%, respectively. Furthermore, blood was obtained at ages 4, 8 and 16 years from 2,605 (63.7%), 2,470 (60.4%) and 2,547 (62.2%) children, respectively. The baseline and follow-up studies were approved by the Regional Ethical Review Board, Karolinska Institutet, Stockholm, Sweden, and the parents of all participating children provided written informed consent.

***Gestational age***

Gestational age was obtained from both the Medical Birth Registry and a questionnaire administered at enrolment based on ultrasound estimations and was used as a continuous variable in the analyses. Preterm birth was defined as a gestational age < 37+0 weeks (< 259 days).

***Methylation measurements***

For this methylation study, we used data from the 4-, 8- and 16-year follow-up. At 4 years, epigenome-wide DNA methylation was measured in 256 Caucasian children, at 8 years methylation was measured in 472 Caucasian children and at 16 years, methylation was measured in 269 Caucasian children. 500 ng DNA per sample underwent bisulfite conversion using the EZ-96 DNA Methylation kit (Zymo Research Corporation, Irvine, USA). Samples were plated onto 96-well plates in randomized order. Samples were processed with the Illumina Infinium HumanMethylation450 BeadChip (Illumina Inc., San Diego, USA). Quality control of analyzed samples was performed using standardized criteria. At 4 years blood methylation data were produced in the Genome Analysis Facility of the University Medical Center Groningen (UMCG) in Holland as part of the MeDALL (Mechanisms of the Development of Allergy) project. DNA methylation data were pre-processed by using the minfi R from the original idat files. Samples that did not provide significant methylation signals in more than 10% of probes (detection P>0.01) were excluded from further analysis. Samples were also excluded in cases of low staining efficiency, low single base extension efficiency, low stripping efficiency of DNA from probes after single base extension, poor hybridization performance, poor bisulphite conversion and high negative control probe staining. For 8 and 16 years, samples were excluded in case of sample call rate <99%, colour balance >3, low staining efficiency, poor extension efficiency, poor hybridization performance, low stripping efficiency after extension and poor bisulfite conversion. We also applied multidimensional scaling (MDS) plot to evaluate gender outliers based on chromosome X data, that produced two separated clusters for male and female. Samples that did not belong to the distinct cluster were removed. Furthermore, we applied median intensity plot for methylated and unmethylated intensity by using the minfi R package (samples below the 10.5 cutoff were excluded). Applying these criteria resulted in exclusion of 8 (8-year DNA) and 2 (16-year DNA) samples, respectively. Probes with a single nucleotide polymorphism in the single base extension site with a frequency of > 10 % at 4 years and of >5% at 8 and 16 years were excluded^4^, as were probes with non-optimal binding (non-mapping or mapping multiple times to either the normal or the bisulphite-converted genome), and the probed belonging to chr X and chr Y, resulting in the exclusion of 46,206, 46,799 and 47,654 probes (4-,8- and 16-year DNA), leaving a total of 439,306, 438,713 and 437,858 probes, respectively, in the analyses. Furthermore, we implemented “DASEN” recommended from wateRmelon package to do signal correction and normalization^24^.

***Covariates***

Information on mode of delivery and parity was collected from the Birth Registry and parity was categorized into 0 and ≥1. Maternal age, sex, maternal smoking status, birth weight and maternal socio-economic status information were collected from a questionnaire administered at enrolment. Maternal socio economic was categorized into blue collar worker, white collar worker and other students, unemployed, housewives. Current age was collected from the 4-, 8- and 16-year questionnaires, respectively. Ever doctor’s diagnosis of asthma was considered to be a selection factor in the 8-year data, but not at 4 and 16 years.

***Cell type correction***

We used the Reinius-based Houseman method^20,25^ with the estimateCellCounts function in the Minfi package^21^ in R^26^ to estimate relative proportions of six white blood cell subtypes (CD4+ T-lymphocytes, CD8+ T-lymphocytes, NK (natural killer) cells, B-lymphocytes, monocytes and granulocytes).

***Batch correction***

Batch correction was attained including the significant (permutation p-value< 10^-4^) principal components derived from the 613 negative control probes presented in 450K arrays. After 10.000 permutations 5 PCs were retained and in additional one batch was also accounted for in the models, based on the bisulfite treatment. The beta-values were batch corrected incorporating these 5 PCs and calculating the residuals of the linear model at 4 years^7^. The covariate batch was also accounted for in the models, based on the bisulfite treatment date at 8 years. At 16 years, the empirical Bayes method via ComBat was applied for batch correction based on sample plate and sentrix position using the sva package in R^27^).

***Exclusion criteria***

For all models we excluded (n=13 at 4 years), (n=23 at 8 years) and (n=20 at 16 years) infants born more than 42+0 weeks (more than 294 days) or multiple birth cases respectively. In the no complication model whose mothers experienced pre-eclampsia, hypertension or diabetes during pregnancy (n=9 at 4 years), (n=13 at 8 years) and (n=11 at 16 years) or delivery start with induction (n=7 at 4 years), (n=18 at 4 years) and (n=10 at 16 years) or caesarean section (n=39 at 4 years), (n=47 at 8 years) and (n=38 at 16 years) were excluded respectively. This information was obtained from questionnaires completed by the mothers shortly after delivery and combined with medical birth records.

##

## California Birth Cohort (CBC)

***Design and study population***

The California Department of Public Health maintains a repository of all neonatal birth bloods as blood dried on a filter paper (Guthrie card), which we term the California Birth Cohort (CBC). These are available for qualified researchers to perform specified health research as monitored by local and State level institutional review boards; all work here was performed under ethics approval at the State of California and University of California. Our current research project using CBC resources is a case-control study of childhood leukemia, the California Childhood Leukemia Study (CCLS) which identifies children with leukemia and matched controls (birthdate, gender, and ethnicity) from selected counties around the State of California^28^. Control subjects were chosen as birthdate, gender, and ethnic matches to leukemia cases. Cases included all consecutive leukemia cases among the counties of recruitment (which varied by time period) during several stages of the CCLS study.

***Gestational age***

Gestational age was collected through a questionnaire that was administered to the mothers at time of enrolment in the study, i.e., at time of their child's diagnosis (or at the equivalent age for controls)

***Methylation measurements***

Approximately 300-500 ng of high molecular weight DNA was extracted from a 1/4 section of a 1.5 cm2 archived neonatal DBS (stored at -20°C from the time of birth) using Qiagen blood card extraction protocol and bisulfite treated using the EZ DNA Methylation-Direct™ Kit (Zymo). Genome-wide DNA methylation was then measured in these bisulfite-converted DNA samples using Illumina(C) Infinium HumanMethylation450 BeadChip arrays. CpG sites with detection p-values > 0.01 were defined as bad CpG sites and discarded. CpG sites with >15% of absence of information (i.e. >15% of total samples) were totally excluded from the analysis. A total of 540 CpGs were excluded. Samples with >15% of bad CpG sites (of the 450K loci) were also excluded from the analysis. The DNA methylation data preprocessing consisted of functional normalization according to Fortin et al.^29^ to control for batch and position effects. Additional correction for probe types was accomplished with BMIQ normalization^30^. Joo et al. have demonstrated that DNA methylation measured by the HM450k array on archived dried blood spots is fully correlated with DNA methylation measured by the same platform on same individuals’ matched frozen buffy coats (correlation coefficient = 0.99), therefore proving that this material is suitable for DNA methylation analyses^31^.

***Covariates***

Birth weight, gender, trichotomous annual household income <=$44,999/<=$74,999/>=$75,000 as maternal social class, dichotomous maternal smoking status, maternal age and mode of delivery, parity were used as covariates in the models. Models were stratified by ethnicity group (Hispanic and Caucasian).

***Cell type correction***

We also adjusted for potential confounding by cell type using estimated cell type proportions calculated from the cord blood cell type reference panel^12^ using the *estimateCellCounts* function in the Minfi R package^19^ to estimate relative proportions of six white blood cell subtypes (CD4+ T-lymphocytes, CD8+ T-lymphocytes, NK (natural killer) cells, B-lymphocytes, monocytes and granulocytes) and nRBC-nucleated red blood cell.

***Batch correction***

Analyses were adjusted for batch by including the Beadchip ID.

***Exclusion criteria***

Information on maternal diabetes and pre-eclampsia was not available, thus we were not able to filter data based on these criteria. For this study we only included control participants, participants with leukemia were excluded. For all models we excluded n=4 infants born more than 42+0 weeks (more than 294 days) in both CBC Hispanic and CBC Caucasian. In the no complication model whose mothers delivery on caesarean section were 33 and 43 in Hispanic and Caucasian respectively were excluded. Information on maternal diabetes and pre-eclampsia was not available, thus we were not able to filter data based on these criteria.

## Center for the Health Assessment of Mothers and Children of Salinas (CHAMACOS)

***Design and study population***

The Center for the Health Assessment of Mothers and Children of Salinas (CHAMACOS) study is a longitudinal birth cohort study of the effects of exposure to pesticides and environmental chemicals on the health and development of Mexican-American children living in the agricultural region of Salinas Valley, CA. Detailed description of the CHAMACOS cohort has previously been published^32,33^. Briefly, 601 pregnant women were enrolled in 1999- 2000 at community clinics and 527 live born singletons were born. Follow up visits occurred at regular intervals throughout childhood. Study protocols were approved by the University of California, Berkeley Committee for Protection of Human Subjects and written informed consent was obtained from all mothers.

***Gestational age***

Gestational age was obtained based on last menstrual period was used as a continuous variable in the analyses. Preterm birth was defined as a gestational age < 37+0 weeks (< 259 days).

***Methylation measurements***

DNA methylation was measured in DNA isolated from the cord blood of 378 CHAMACOS newborns by Illumina Infinium HumanMethylation450 (450K) BeadChips. DNA samples were bisulfite converted using Zymo Bisulfite Conversion Kits (Zymo Research, Irvine, CA), whole genome amplified, enzymatically fragmented, purified, and applied to the 450K BeadChips (Illumina, San Diego, CA) according to manufacturer protocol. 450K BeadChips were handled by robotics and analyzed using the Illumina Hi-Scan system. DNA methylation was measured at 485,512 CpG sites. Probe signal intensities were extracted by Illumina GenomeStudio software (version XXV2011.1, Methylation Module 1.9) methylation module and background subtracted. QA/QC was performed systematically by assessment of assay repeatability batch effects using 38 technical replicates, and data quality established as previously described^34^. Quality was also ensured by only retaining samples where 95% of sites assayed had detection P> 0.01. The same threshold (95% detection at p>0.01) was imposed to CpGs as well (n= 460 removed). Sites with annotated probe SNPs and with common SNPs (minor allele frequency >5%) within 50bp of the target identified in the MXL (Mexican ancestry in Los Angeles, California) HapMap population were excluded from analysis (n=49,748). This left a total of 435,369 CpGs in the analysis. In the secondary model, color channel bias, batch effects and difference in Infinium chemistry were minimized by application of ASMN algorithm^30^, followed by BMIQ normalization^30^.

***Covariates***

Maternal age, parity and education were assessed by participant interview at baseline visit (~13 weeks gestation). Maternal age was treated as a continuous variable. Parity was coded as a binary variable, with 0 as the baseline and ≥1 as the alternative. Maternal education was also treated as a categorical variable, with three levels: no more than a 6^th^ grade education, 6^th^ grade education by not high school graduate, or having completed high school education or beyond. Birth weight (grams) was collected from medical records and abstracted by a registered nurse.

***Cell type correction***

We also adjusted for potential confounding by cell type using estimated cell type proportions calculated from the cord blood cell type reference panel^12^ using the *estimateCellCounts* function in the Minfi R package^21^ to estimate relative proportions of six white blood cell subtypes (CD4+ T-lymphocytes, CD8+ T-lymphocytes, NK (natural killer) cells, B-lymphocytes, monocytes and granulocytes) and nRBC-nucleated red blood cell.

***Batch correction***

Analysis was adjusted for batch effects by including 450K plate (n=10) as additional covariates.

***Exclusion criteria***

## For all models we excluded n=6 infants born more than 42+0 weeks (more than 294 days) or multiple birth cases. In the no complication model whose mothers experienced pre-eclampsia, hypertension or diabetes (n=38) during pregnancy or delivery start with induction (n=79) or caesarean section (n=79) were excluded. This information was obtained from questionnaires completed by the mothers’ shortly after delivery and combined with medical birth records.

## Children’s Health Study (CHS)

***Design and study population***

The Children’s Health Study (CHS) is a population-based prospective cohort study from age 5 onwards in Southern California, which has been described in detail elsewhere^35^. The study protocol was approved by the University of Southern California Institutional Review Board and informed, written consent and assent were provided by the parents and children respectively. A total of 5341 children were recruited, all of whom were born between 1995 and 1997 and were followed until age 18.

***Gestational age***

Gestational age at birth was obtained from California birth certificates (n=273).

***Methylation measurements***

Based on the availability of newborn bloodspots archived by the state of California and our ability to link CHS subjects with California birth records, a subset of 273 children was selected for a sub-study in which epigenome-wide DNA methylation was assessed in newborn bloodspots using the Infinium HumanMethylation450 BeadChip (HM450). Laboratory personnel performing DNA methylation analysis were blinded to study subject information. DNA was extracted from whole blood cells using the QiaAmp DNA blood kit (Qiagen Inc, Valencia, CA) and stored at -80 degrees Celcius. 700-1000ng of genomic DNA from each sample was treated with bisulfite using the EZ-96 DNA Methylation Kit™ (Zymo Research, Irvine, CA, USA), according to the manufacturer’s recommended protocol and eluted in 18 μl. Chips were analysed in three batches over a period of a couple of months. The results of the Infinium HumanMethylation450 BeadChip (HM450) were compiled for each locus and were reported as beta (β) values. A normal-exponential background correction with dye bias correction was applied to the raw intensities at the array level to reduce background noise^36^. We then normalized each sample’s methylation values to have the same quantiles to address sample to sample variability^37^. CpG loci on the HM450 array were removed from analyses if they were on the X and Y chromosomes, or if they contained SNPs, deletions, repeats, or if they have more than 10% missing values, leaving a total of 384,310 probes for analysis. Beta values were considered as outliers and were removed if they fall below Quartile 1-3×IQR or above Quartile 3+3×IQR.

***Covariates***

Newborn sex, newborn birth weight, mode of delivery, maternal age at birth and maternal parity were obtained from California birth certificates. Data on maternal social class and maternal smoking status during pregnancy were obtained from parent-completed questionnaires at study entry when the subjects were around 6 years old. Ancestry was assessed from CHS genome-wide genotypic data using the program STRUCTURE from a set of ancestral informative markers that were scaled to represent the proportion of African American, Asian, Native American and white admixture^38^.

***Cell type correction***

Seven cord blood cell sub-populations were estimated using regression calibration approach algorithm described by Houseman *et al.* that was implemented in minfi^20,21^. Estimated cord blood cell subpopulations (CD8+ T-lymphocytes, CD4+ T-lymphocytes, natural killer cells, B-lymphocytes, monocytes, granulocytes and nucleated red blood cells) were subsequently included as linear predictors in regression models.

***Batch correction***

We additionally corrected the analyses for batch effect by including the Illumina Infinium HumanMethylation450 BeadChip plate number (n=3).

***Exclusion criteria***

For all models we excluded n=11 infants born more than 42+0 weeks (more than 294 days) or multiple birth cases. In the no complication model, mothers who experienced pre-eclampsia, hypertension or diabetes (n=5) during pregnancy or delivery start with induction (n=71) or caesarean section (n=43) were excluded. This information was obtained from California birth certificates. Additionally, we excluded subjects with missing data on gestational age or any of the other covariates in the model (birth weight, sex, mode of delivery, maternal education level, maternal smoking status, maternal age, parity, methylation plate, estimated cell proportions and ancestry), leaving 120 subjects in the main analysis.

## Etude des Déterminants pré et post natals du développement et de la santé de l′Enfant (EDEN)

***Design and study population***

The EDEN (Etude des Déterminants pré et post natals du développement et de la santé de l′Enfant) study is a prospective Birth Cohort Study (https://eden.vjf.inserm.fr/), which has been described in detail elsewhere^39^. The Pregnant women visited for a prenatal visit at the departments of Obstetrics and Gynecology of the University Hospital of Nancy and Poitiers before their twenty-fourth (24th) week of amenorrhea were invited to participate. Enrollment started in February 2003 in Poitiers and September 2003 in Nancy; it lasted 27 months in each centre. Among eligible women, 55% (2002 women) accepted to participate. The study was approved by the ethical committees "Comité Consultatif pour la Protection des Personnes dans la Recherche Biomédicale", Le Kremlin-Bicêtre University hospital, and "Commission Nationale de l’Informatique et des Libertés".

***Gestational age***

The gestational age was obtained from both the medical birth registry and a questionnaire administered at the time of enrolment based on ultrasound estimation and was used as a continuous variable in the analyses. Preterm birth was defined as gestational age <37 weeks.

***Methylation measurements***

DNA was extracted from 150 cord blood samples. Amplified and genomic DNA samples are now stored in 96- well plates at -80°C. More than 40 single nucleotide polymorphisms (SNPs) have been genotyped either from genomic or from amplified DNA. The samples underwent bisulfite treatment using the EZ-96 DNA Methylation kit (Zymo Research Corporation, Irvine, USA), and were subsequently processed with the Illumina Infinium Human Methylation 450 BeadChip (Illumina Inc., San Diego, USA). In total, 439,306 CpGs are available in children with DNA measurements.

***Covariates***

The information on parity and mode of delivery was were collected from the birth registry and were categorized in to 0 and ≥1. Maternal age, sex of the child, maternal smoking status, birth weight and maternal socio-economic status information were collected from a questionnaire at enrolment. Maternal socio-economic status was categorized into low, medium and high income.

***Cell type correction***

We used the Bakulski-based Houseman method^12,20^ with the estimate Cell Counts function in the Minfi package^21^in R^26^ to estimate relative proportions of six white blood cell subtypes (CD4+ T-lymphocytes, CD8+ T-lymphocytes, natural killer (NK) cells, B-lymphocytes, monocytes and granulocytes).

***Batch correction***

Batch correction was attained including the significant (permutation p-value< 10^-4^) principal components (PCs) derived from the 613 negative control probes^40^ presented in 450K arrays because these negative control probes did not relate to biological variation. After 10000 permutations 5 PCs were retained. The beta-values were batch corrected incorporating these 5 PCs and calculating the residuals of the linear model at 5 years^7^. The covariate one batch was also accounted for in the models, based on the bisulfite treatment.

***Exclusion criteria***

In EDEN cohort infants born more than 42+0 weeks (more than 294 days) or multiple birth cases were excluded respectively from the study. In the no complication models whose mothers experienced pre-eclampsia hypertension or diabetes (n=16) or delivery start with induction (n=27) or caesarean setion (n=17) were excluded. This information was obtained from questionnaires completed by the mothers’ shortly after delivery and combined with medical birth records.

**EXPOsOMICS: RHEA, ENVIR*ON*AGE and Piccolipiù**

***Design and Study Population***

Within the EXPOsOMICS collaborative European project, a combination of three population-based birth cohorts, ENVIRonmental influence ON AGEing in early life (ENVIR*ON*AGE), Rhea and Piccolipiù, was established to conduct DNA methylation analyses^41^.The phenotypic variables were harmonized across the three cohorts, and their biospecimen were semi-randomized on the DNA methylation arrays such that the latter would incorporate proportional representations of the three cohorts and that batch effects do not completely confound with biological covariates of interest.

RHEA: The mother-child Rhea study in Crete is a prospective cohort examining a population sample of pregnant women and their children, at the prefecture of Heraklion (n = 1500)^42^.Ethical approval was obtained from the local ethics committee in Heraklion and parents provided written informed consent. The study aims are to evaluate a) nutritional, environmental, biological and psychosocial exposures in the prenatal period and in early childhood, b) the association of these exposures with the development of the foetus and the child, c) mother’s health during and after pregnancy, and d) genetic susceptibility and the interactions between genetic and environmental factors affecting child health. A set of 100 newborns from the Rhea cohort is included in the EXPOsOMICS children studies, for which data on cord blood DNA methylation is available. A subsample of 91 newborns with complete covariate information was included in the current analysis.

ENVIR*ON*AGE: The ongoing Belgian birth cohort ENVIR*ON*AGE (ENVIRomental influence *ON* early AGEing) is a longitudinal study, starting with recruitment at birth and follow-up at the age of 4-6 years and at preadolescent age^43^. The birth cohort study is designed with a strong focus on molecular mechanisms to understand the determinants of molecular ageing in early life and its role in the developmental origins of health and disease. ENVIR*ON*AGE recruits’ mother-newborn pairs when they arrive for delivery at the East-Limburg Hospital in Genk, Belgium. Mothers without planned caesarean section and able to fill out a questionnaire in Dutch are eligible for participation. Procedures are approved by the Ethical Committee of Hasselt University and the East-Limburg Hospital and recruitment is carried out according to the Helsinki declaration. A set of 200 newborns from the ENVIR*ON*AGE cohort is included in the EXPOsOMICS children studies, for which data on cord blood DNA methylation is available. A subsample of 189 newborns with complete covariate information was included in the current analysis.

Piccolipiù**:** Piccolipiù is a multicentric Italian birth cohort that recruited 3338 newborns and their mothers in 5 centres: Turin, Trieste, Florence, Viareggio and Rome between 2011 and 2015. Details about the study protocol have been published elsewhere^44^. Families were contacted 6, 12, 24 and 48 months after delivery to collect follow-up information using questionnaires, and children underwent a medical examination at 4 years of age. Cord blood was collected and stored in a centralized biobank. Ethical approvals have been obtained from the Ethics committees of the Local Health Unit Roma E (management centre), of the Istituto Superiore di Sanità (National Institute of Public Health) and of each local centre. Parents provided written informed consent. A sample of 99 newborns from the Turin centre - who were resident in Turin, with growth data at birth and until at least 2 years of age and full availability of cord blood samples - was included in the EXPOsOMICS Children Studies. A subsample of 96 newborns with complete covariate information was included in the current analysis.

***Methylation Measurements for Rhea, ENVIRONAGE, Piccolipiu***

Aliquots of cord blood samples (collected and frozen at birth at -80°C) were shipped on dry ice to the Epigenetics Group at the International Agency for Research on Cancer (IARC), Lyon, France, where DNA was extracted (QIAamp 96 DNA Blood Kit, Qiagen 51161), quantified (Quant-iT PicoGreen dsDNA Assay Kit, Molecular Probes P7589) and bisulfite converted (600 ng of DNA using EZ-96 DNA Methylation kit, Zymo Research D5004). DNA methylation was measured at 485 577 CpGs using Illumina Infinium HumanMethylation450 BeadChip (Illumina Inc., San Diego, USA). The arrays were designed such that batch effects (e.g. sample position and intra- and inter-variability in arrays and chips) do not completely confound with biological covariates of interest. This design allows the retention of biological variation even after correction of technical variation.

Raw intensity (.idat) files were handled in R using the minfi package to calculate the methylation level at each CpG as the beta-value (β=intensity of the methylated allele (M)/(intensity of the unmethylated allele (U) + intensity of the methylated allele (M) + 100)), and the data were exported for quality control and processing. Methylation features were filtered from cross-reactive probes and low-quality probes (probes having bead counts < 3 in at least 5% of samples). Data quality was further assessed using box plots for the distribution of methylated and unmethylated signals, and multidimensional scaling plots and unsupervised clustering were used to check for sample outliers and potential gender mismatches, which were removed from the analysis. Also, samples having >1% of CpG sites with a detection P-value >0.05 were removed. The remaining dataset was normalized using the funnorm normalization of the minfi package. Cohort and batch (sample plate, sentrix position) effects were then corrected with surrogate variable analysis (SVA).

***Gestational age***

RHEA: Gestational age was obtained from the last menstrual period and if missing ultrasound estimations were used. Gestational age was used as a continuous variable in the analyses.

ENVIR*ON*AGE: Gestational age was obtained from ultrasound estimations and was used as a continuous variable in the analyses.

Piccolipiù: Gestational age was obtained from last menstrual period and was used as a continuous variable in the analyses.

***Covariates***

RHEA: Information on maternal age, smoking, education, parity and origin was obtained from questionnaires administered at recruitment between week 12 and 14 of pregnancy (at the time of the first routinely scheduled major ultrasound test). Maternal smoking status was categorized as smoking during pregnancy yes or no. Maternal education was defined as primary school, secondary school, and university degree or higher. Maternal race was defined as maternal Greek origin or not. As this cohort is quite homogeneous with respect to ethnicity, the ancestry of all newborns was set to European for the current study. Information on gender, mode of delivery and birth weight was collected by the midwives during the birth admission.

ENVIR*ON*AGE: The variables gender, delivery mode, birth weight and maternal age were retrieved from the medical records of the hospital. Information on maternal smoking, maternal education, parity and newborn’s ethnicity was obtained from questionnaires filled out after delivery. Maternal smoking status was defined as smoking during pregnancy yes or no. Newborns were classified as European when at least two grandparents were European, and non-European when at least three grandparents were of non-European origin. Maternal education was classified as primary school, secondary school, and university degree or higher.

Piccolipiù: Information on maternal age, smoking, birth weight, parity, mode of delivery, education and origin was obtained from a baseline questionnaire administered at recruitment (in the Turin centre participants are recruited at admittance to the hospital for delivery). Maternal smoking status was defined as smoking during pregnancy yes or no. Maternal education was categorized as primary school, secondary school, and university degree or higher. With respect to race, information on country of birth of the mother and of the parents of the mother is available. As this cohort is quite homogeneous with respect to ethnicity, the ancestry of all newborns was set to European for the current study.

***Cell type correction***

We used the Bakulski-based Houseman method^12,20^ with the estimate Cell Counts function in the Minfi package^21^in R^26^ to estimate relative proportions of six white blood cell subtypes (CD4+ T-lymphocytes, CD8+ T-lymphocytes, natural killer (NK) cells, B-lymphocytes, monocytes and granulocytes).

***Batch correction***

We additionally corrected the analyses for batch effect using surrogate variable analysis (SVA).

***Exclusion criteria***

In the methylation subset of the EXPOsOMICS cohort we excluded infants born more than 42+0 weeks (more than 294 days) or multiple birth cases were excluded respectively from the study. In the no complication models whose mothers experienced pre-eclampsia hypertension or diabetes (n=44) or delivery with caesarean setion (n=95) were excluded. This information was obtained from questionnaires completed by the mothers’ shortly after delivery and combined with medical birth records.

## Groningen Expert Center for Kids with Obesity (GECKO Drenthe)

***Design and study population***

The Groningen Expert Center for Kids with Obesity (GECKO) Drenthe cohort is a population-based prospective birth cohort study in Drenthe, a northern province in the Netherlands. All mothers of infants born between April 2006 and April 2007 were invited to participate during the third trimester of pregnancy. Of all 4,778 infants born in this period, a total of 2,874 newborns (60%) participated in the study. This study has been approved by the Medical Ethical Committee of the University Medical Center Groningen and parents of all participants gave written informed consent. Details about this cohort have been described elsewhere^45^.

***Gestational age***

Gestational age was obtained from both the Medical Birth Registry and a questionnaire administered at enrolment based on ultrasound estimations and was used as a continuous variable (days) in the analyses. Preterm birth was defined as a gestational age < 37+0 weeks (< 259 days) and for the methylation sub sample we excluded preterm births.

***Methylation measurements***

Within the GECKO Drenthe birth cohort, we selected 258 infants for the methylation study: 129 exposed to maternal smoking during pregnancy and 129 unexposed to both maternal and paternal smoking during pregnancy. From these 258 infants, we used DNA which was extracted from cord blood for the epigenome-wide DNA methylation analyses. To limit batch effects, we randomized all samples over the 96-well plates, based on gender and smoking status. Samples (500 ng per sample) were placed on three 96-well plates. Bisulfite conversion was performed using the EZ-96 DNA methylation kit (Zymo research Corporation, Irvine, USA). Then we used the Infinium HumanMethylation450 BeadChip (Illumina Inc., San Diego, USA) to measure the methylation level as a beta value ranging from zero (no methylation) to one (complete methylation). We used minfi to calculate betas and p values for all 485,577 CpGs. During the quality control, we excluded two males that clustered in the female group, based on X chromosome betas, which was probably due to maternal blood contamination. We performed Illumina-suggested background normalization, colour correction and Subset-quantile Within Array Normalization (SWAN). We excluded one sample because it did not meet the criteria of ≥99% of the CpGs with detection p value <0.05. This resulted in 129 children exposed to maternal smoking during pregnancy and 126 unexposed children. We excluded control probes, probes on X or Y chromosomes and probes that did not meet our criteria of a detection p value of <0.05 in ≥99% of the samples, resulting in 465,891 remaining CpGs.

***Covariates***

Information on mode of delivery and parity was recorded by obstetricians or midwives in the delivery room or abstracted from obstetric records and parity was categorized into 0 and ≥1. Maternal age, sex, maternal smoking status, birth weight and maternal socio-economic status information were self-reported in a questionnaire administered at enrolment. Maternal socio economic was categorized into low/average vs university educated.

***Cell type correction***

We used the Bakulski-based Houseman method^12,20^ with the estimate Cell Counts function in the Minfi package^21^in R^26^ to estimate relative proportions of six white blood cell subtypes (CD4+ T-lymphocytes, CD8+ T-lymphocytes, natural killer (NK) cells, B-lymphocytes, monocytes and granulocytes).

***Batch correction***

We corrected the analyses for batch effect by including the Illumina Infinium HumanMethylation450 BeadChip number (n=3).

***Exclusion criteria***

In the methylation subset of the GECKO Drenthe cohort we excluded infants born preterm (<37 weeks) or more than 42+0 weeks (more than 294 days), multiple birth cases and those whose mothers experienced pre-eclampsia or diabetes during pregnancy, this information was obtained from questionnaires completed by the mothers shortly after delivery and combined with medical birth records.

## The Genetics of Glucose regulation in Gestation and Growth (Gen3G)

***Design and study population***

The Genetics of Glucose regulation in Gestation and Growth (Gen3G) is a prospective observational cohort study aiming to increase our understanding of biological, environmental, and genetic determinants of glucose regulation during pregnancy and their impact on foetal development and was described in details previously^46^. In brief, we recruited a total of 1034 pregnant women aged ≥18 years old between January 2010 and June 2013 representing the general population of women in reproductive age receiving care at our institution. Women were excluded if they had non-singleton pregnancy, known pre-pregnancy diabetes or overt diabetes diagnosed based on biochemical screening that we performed at first trimester. The study protocol was approved by the Centre Hospitalier Universitaire de Sherbrooke (CHUS) ethic committee board and every participant gave written informed consent before enrolment in the study, in accordance with the Declaration of Helsinki.

***Gestational age***

Gestational age was obtained from the medical records: in clinic, gestational age is usually derived from last menstrual period (LMP), but when there was a discrepancy of more than 5 days between LMP and crown-rump length (CRL) estimated gestational age, CRL estimated gestational age was then used. We used gestational age as a continuous variable in the analyses. Preterm birth was defined as a gestational age < 37+0 weeks (< 259 days).

***Methylation measurements***

Within the Gen3G birth cohort we randomly selected 182 infants for the methylation study in the single pregnancies without gestational diabetes. From these 182 infants, we used DNA which was extracted from cord blood for the epigenome-wide DNA methylation analyses. To limit batch effects, we randomized all samples over the 96-well plates. Samples (500 ng per sample) were placed on three 96-well plates. Bisulfite conversion was performed using the EZ-96 DNA methylation kit (Zymo research Corporation, Irvine, USA). Then we used the Infinium HumanMethylation450 BeadChip (Illumina Inc., San Diego, USA) to measure the methylation level as a beta value ranging from 0 (no methylation) to 1 (complete methylation). During the quality control, we excluded 5 participants that clustered in the wrong sex group and one that was considered an outlier from the MDS plot. Finally, we excluded 9 samples of the current analyses because of missing values. We performed DASEN normalization from the watermelon package. This resulted in 167 children. Models after removing maternal and perinatal complications were done on 83 children. We excluded probes that did not meet our criteria of a detection p value of <0.01 in ≥80% of the samples and that overlapped with SNPs, had multiple locations on the genome or was on the X chromosome, resulting in 434 476 remaining CpGs.

***Covariates***

Gender, birth weight and mode of delivery were collected from medical records. For mode of delivery, we use only three categories (vaginal delivery spontaneous, vaginal delivery induced and caesarean section delivery) because there was no indication if the caesarean was elective or urgent. Maternal age was collected by questionnaire at first trimester visit. Maternal smoking was derived in three categories (no smoking in pregnancy, stop in the beginning of pregnancy and smoked during pregnancy) by comparison between the last menstrual period date and the smoking ending date (collected by questionnaire at first trimester only). Smoking was not included in models after removing maternal and perinatal complications because of small numbers in categories. Parity was defined as the number of term pregnancies as asked in the first trimester questionnaire. We did not adjust for maternal social class since data are not available in our study.

***Cell type correction***

We used the Bakulski -based Houseman method^12,20^ with the estimate Cell Counts function in the Minfi package^21^ in R^26^ to estimate relative proportions of six white blood cell subtypes (CD4+ T-lymphocytes, CD8+ T-lymphocytes, natural killer (NK) cells, B-lymphocytes, monocytes and granulocytes).

***Batch correction***

Five surrogate variables were generated and included in models to adjust for technical batch, based on the sentrix plate number.

***Exclusion criteria***

## In the no complication model whose mothers experienced pre-eclampsia hypertension or diabetes (n=2) during pregnancy or delivery start with induction (n=54) or caesarean section (n=28) were excluded. This information was obtained from the medical records.

## The Generation R Study

***Design and study population***

The Generation R Study is a population-based prospective cohort study from early pregnancy onwards in Rotterdam, the Netherlands^47,48^. The study was approved by the local Medical Ethics Committee of Erasmus MC (MEC 198.782/2001/31). Pregnant women with a due date between April 2002 and January 2006 and living in Rotterdam were enrolled. Of all eligible women, 61% participated in the study. Written informed consent was obtained for all participants. In total, 9,778 mothers were enrolled in the study^47^.

***Gestational age***

Information on gestational age (GA, in days) at birth was obtained from medical records and was established using first trimester ultrasound. Women with a gestational age of more than 42+0 weeks (more than 294 days) were excluded from all models. Preterm birth was defined as a GA < 37+0 weeks (< 259 days).

***Methylation measurements***

DNA extracted from cord blood from children of European-ancestry was used for this analysis. 500 ng DNA extracted (using the salting-out method) from cord blood per sample underwent bisulfite conversion using the EZ-96 DNA Methylation kit (Shallow) (Zymo Research Corporation, Irvine, USA). Samples were plated onto 96-well plates in no specific order. Samples were processed with the Illumina Infinium HumanMethylation450 BeadChip (Illumina Inc., San Diego, USA) which analyses methylation at 485,577 CpGs.

Quality control of analysed samples was performed using standardized criteria. Samples were excluded in case of low sample call rate (<99%), colour balance >3, low staining efficiency, poor extension efficiency, poor hybridization performance, low stripping efficiency after extension, poor bisulfite conversion and sex mismatch. After quality control, a total of 969 Generation R samples remained in the analysis. Probes with a single nucleotide polymorphism in the single base extension site with a frequency of > 1% in the GoNLv4 reference panel were excluded^49^, as were probes with non-optimal binding (non-mapping or mapping multiple times to either the normal or the bisulphite-converted Genome^50^), resulting in the exclusion of 49,564 probes, leaving a total of 436,013 probes in the analysis.

We ran DASES normalization using a pipeline adapted from that developed by Touleimat and Tost^37^. DASES normalization includes background adjustment, between-array normalization applied to type I and type II probes separately, and dye bias correction applied to type I and type II probes separately and is based on the DASEN method described by Pidsley *et al*, but adds the dye bias correction, which is not included in DASEN^24^.

***Covariates***

We obtained information on maternal age, social class, smoking status and parity from questionnaires during pregnancy. Maternal social class was defined by maternal education and divided into lower (no education, primary or secondary school finished) and higher (higher education finished). Smoking status was categorized as no smoking, stopped early in pregnancy, or continued smoking. Parity was classified as nulliparity or multiparity. Child sex, mode of delivery and birth weight in grams were obtained from midwife or hospital registries.

***Cell type correction***

Cell type correction was applied using the reference-based Houseman method^20^ in the minfi package^19^ in R^26^ using the cord blood-specific Bakulski reference^12^. This method estimates the relative proportions of six white blood cell subtypes (CD4+ T-lymphocytes, CD8+ T-lymphocytes, NK (natural killer) cells, B-lymphocytes, monocytes, granulocytes) and nucleated red blood cells, based on a standard reference of cord blood samples.

***Batch correction***

We additionally corrected for batch effect by adjusting for plate number.

***Exclusion criteria***

For all models we excluded one of each sibling pair, chosen based on data availability and if all data were complete, randomly (17 excluded), postterm born children (GA > 42 + 0 weeks; 58 excluded) and participants with missing observations for any of the covariates (132 excluded). For the no complication model we additionally excluded women who experienced pre-eclampsia, gestational hypertension or HELLP (54 excluded), gestational diabetes (3 further excluded), induced delivery (141 further excluded), caesarean section (70 further excluded), or participants with missing observations for any of the complications (8 excluded). For the model without preterm born children (GA < 37 + 0 weeks), 11 participants were excluded.

## Genetics of Overweight Young Adults (GOYA)

***Design and study population***

The Genetics of Overweight Young Adults (GOYA) study is described in^51,52^. It is nested within the Danish National Birth Cohort (DNBC) who included 91 387 pregnant women during 1996–2002. Of 67 853 women who had given birth to a live born infant, had provided a blood sample during pregnancy and had BMI information available, 3.6% of these women with the largest residuals from the regression of BMI on age and parity (all entered as continuous variables) were selected for GOYA. The BMI for these 2451 women ranged from 32.6 to 64.4. From the remaining cohort, a random sample of similar size (2450) was also selected. In total, 3908 mothers were successfully genotyped. DNA methylation data were generated for the offspring of 1000 mothers in the GOYA study, equally distributed between “cases” with a BMI>32 and “controls” who were sampled from the remaining BMI distribution. Phenotype data was collected during four telephone interviews, two interviews in pregnancy at approximately 16- and 30-weeks’ gestation and two interviews at six months and 18 months postpartum. Further Information about the child (for example, childhood BMI, asthma) was obtained from a questionnaire seven years after the birth. Register linkage was used to identify diseases treated in hospital. All participants in the DNBC gave written informed consent and the collection and use of their data has ethics approval. Ethnicity was obtained using register data.

***Gestational age***

Gestational age at birth was extracted from birth records.

***Methylation measurements***

Cord blood was collected according to standard procedures, spun and frozen at -80˚C. DNA methylation analysis and data pre-processing were performed at the University of Bristol. Following extraction, DNA was bisulfite converted using the Zymo EZ DNA MethylationTM kit (Zymo, Irvine, CA). Following conversion, the genome-wide methylation status of over 485,000 CpG sites was measured using the Illumina Infinium® HumanMethylation450k BeadChip assay according to the standard protocol. The arrays were scanned using an Illumina iScan and initial quality review was assessed using GenomeStudio (version 2011.1). The level of methylation is expressed as a “Beta” value (β-value), ranging from 0 (no cytosine methylation) to 1 (complete cytosine methylation). Samples from all time-points in ARIES were distributed across slides using a semi-random approach (sampling criteria were in place to ensure that all time-points were represented on each array) to minimize the possibility of confounding by batch effects. Samples failing quality control (average probe detection p-value ≥ 0.01) were repeated. As an additional quality control step genotype probes on the HumanMethylation450k were compared between samples from the same individual and against SNP-chip data to identify and remove any sample mismatches. Data were normalized using the functional normalization approach in the Minfi R package.

We removed probes that had a detection P-value >0.05 for >5% of samples, probes on the X or Y chromosomes and SNPs (rs probes). 473 864 probes remained.

***Covariates***

Data on maternal parity, socio-economic status, smoking and pre-pregnancy body mass index were collected via a telephone interview at around 16 weeks’ gestation. Maternal age was derived from the mother’s report of her own date of birth. Newborn sex, parity, mode of delivery and birthweight were extracted from birth records.

***Cell type correction***

We used the Bakulski-based Houseman method^12,20^ with the estimate Cell Counts function in the Minfi package^21^in R^26^ to estimate relative proportions of six white blood cell subtypes (CD4+ T-lymphocytes, CD8+ T-lymphocytes, natural killer (NK) cells, B-lymphocytes, monocytes and granulocytes).

***Batch correction***

Ten surrogate variables were generated using the sva package^22^ in R and included in models to adjust for technical batch.

***Exclusion criteria***

## For all models we excluded n=43 infants born more than 42+0 weeks (more than 294 days) or multiple birth cases.

## Infancia y Medio Ambiente (INMA)

***Design and study population***

The INMA—INfancia y Medio Ambiente—(Environment and Childhood) Project is a network of birth cohorts in Spain that aims to study the role of environmental pollutants in air, water and diet during pregnancy and early childhood in relation to child growth and development^53^. Mothers were enrolled at week 12 of pregnancy from 1997 to 2008 in seven regions of Spain (Flix, Granada, Menorca, Asturias, Gipuzkoa, Sabadell and Valencia). The cohort consisted of 3,768 children at birth. During the follow-up visits information on environmental exposures and health outcomes (reproductive, growth and obesity, lung function, allergies and neurovelopment) were assessed through questionnaires, biomarker measurements, clinical data, and physical exploration. The study website contains details of the design and data available in INMA project (http://www.proyectoinma.org/). The study was approved by the Ethical Committees of each participating centre and written consent was obtained from parents.

In this study, DNA methylation data was analyzed for 370 cord blood samples from INMA Sabadell subcohort, and 191 peripheral blood from the same children followed up at age 4 who were born at a gestational age less than 42 weeks (<294 days of gestation). All of them were of European ancestry according to self-reported data.

***Gestational age***

We calculated gestational age from the date of the last menstrual period (LMP) reported at recruitment and confirmed using estimates based on the first ultrasound examination (about 12th week of gestation). When the difference between the LMP reported at recruitment and estimated from the ultrasound was ≥ 7 days, we estimated LMP using the crown-rump length^54^.

***Methylation measurements***

Both umbilical cord blood and peripheral blood collected at 4y of age were extracted using the Chemagen kit (Perkin Elmer). DNA concentration was determined by a NanoDrop spectrophotometer (Thermo Scientific) and with the Quant-iT PicoGreen dsDNA Assay Kit (Life Technologies).

Blood methylation data was measured in two different facilities: a first batch of umbilical cord blood samples(n=192, of which 184 were included in this analysis) and 4 year old peripheral blood (n=201, of which 191 were included in this analysis) was analyzed in the Genome Analysis Facility of the University Medical Center Groningen (UMCG) in the Netherlands as part of the MeDALL (Mechanisms of the Development of Allergy) project, a second batch of umbilical cord blood samples only (n=193, of which 186 were included in the current analysis) was analyzed at IDIBELL Bellvitge Biomedical Research Institute, Barcelona (IDIBELL) as part of the BREATHE project (BRain dEvelopment and Air polluTion ultrafine particles in scHool children). Within each batch, samples were randomized and processed following the Illumina protocol for the Infinium HumanMethylation450 BeadChip. Briefly, 500 ng of DNA was bisulfite-converted using the EZ 96-DNA methylation kit, and DNA methylation was measured through hybridization on the BeadChips.

DNA methylation data were pre-processed in R using minfi^19^, from the original idat files extracted from the HiScanSQ scanner. Samples with low overall quality according to MethylAid^55^, and call rate <98% were excluded. Probes with call rate <95%, assuming a detection P value below 10E-16, were excluded from the analyses^56^. Further, we assessed the methylation distribution of the X-chromosome to verify gender^29^. Data was normalized using the functional normalization with background correction (Noob) and dye bias correction^29^. We removed the 65 SNPs probes. A total of 561 samples, 370 cord blood and 191 4y-old blood, and 476,946 CpG probes were analyzed for this study. Although we combined samples selected for both MeDALL and BREATHE projects, as the initial selection criteria for MeDALL samples included cases and controls with allergic history (asthma, eczema and/or rhinitis), 116 out of 370 cord blood samples and 68 of 191 peripheral blood were classified as having any of the three diseases and this covariate was still significant in the principal component regression, all the models were adjusted for this covariate.

***Covariates***

Models were adjusted for subject sex, maternal age at birth, maternal social class, maternal smoking status, parity, mode of delivery, batch for the models of cord blood (MeDALL, BREATHE, see methylation measurements above), and selection criteria (asthma, eczema and/or rhinitis).

Infant sex was abstracted from clinical records. Data on maternal age at birth was self-reported and collected using a questionnaire at enrolment (week 12 of pregnancy). Maternal socioeconomic status was based on maternal occupation at pregnancy, it was categorized into three levels: low (levels V/VI semi-skilled/unskilled occupations), medium (levels III/IV skilled manual/non-manual) or high (managers/technicians). Pregnant women were asked whether they were current smokers (at week 32 of pregnancy) and if so, how much. They were also asked if they had stopped smoking due to pregnancy and when (before pregnancy or at what month of pregnancy). Any smoking was defined as smoking any number of cigarettes at any time during pregnancy. Parity was defined as nulliparous (first children born) or multiparous (previous pregnancies with live birth). Mode of deliver was abstracted from medical records and was classified as vaginal delivery or cesarean section. For models at 4 yr of age, the child age at sample collection was included as a covariate.

***Cell type correction***

We used the *estimateCellCounts* function from minfi which estimates cell proportions using constrained projection/quadratic programming through the Houseman method^20^. For umbilical cord blood we estimated seven blood cell subtypes (CD4+ T-lymphocytes, CD8+ T-lymphocytes, NK (natural killer) cells, B-lymphocytes, monocytes, granulocytes and nucleated red blood cells) using the Bakulski reference^12^. For the samples of children at 4 yr we estimated six cell types (CD4+ T-lymphocytes, CD8+ T-lymphocytes, NK (natural killer) cells, B-lymphocytes, monocytes, and granulocytes) using the adult Reinius reference^25^.

***Batch correction***

We used ComBat^27^ to correct for the two laboratory processing batches (while preserving the variability for the age and sex covariates) using the package sva^22^. As these two laboratory processing batches were still significant in the principal component regression after ComBat, we also included this covariate in the umbilical cord blood models^57^.

***Exclusion criteria***

For this analysis we only excluded children that were born more than 42 weeks of gestation (>294 days). 15 samples in the umbilical cord blood group, and 10 samples in the 4 yr group born more than 42 weeks of gestational age were excluded. In the no pregnancy complication models, we excluded children whose mothers experienced high blood pressure or diabetes mellitus (n= 79 in newborns and n= 50 at 4yr) during pregnancy; those children with induced vaginal delivery (n= 74 for cord blood and n= 32 at 4 yr); and those children born through c-section (n=28 for cord blood and n=14 at 4 yr). Subjects with missing information of complications or mode of delivery were also excluded from the models (n=110 for cord blood and n= 50 at age 4). Pregnancy complications were abstracted from medical records.

## Isle of Wight Birth Cohort (IOW F1)

***Design and study population***

A whole population birth cohort was established on the Isle of Wight, UK, in 1989 to prospectively study the natural history of allergic diseases from birth onwards^58,59^. Both the Isle of Wight and the study population are 99% Caucasian. Ethics approvals were obtained from the Isle of Wight Local Research Ethics Committee (now named the National Research Ethics Service, NRES Committee South Central –Southampton B) at recruitment and for the 1, 2, 4, 10- and 18-years follow-up. Of the 1536 children born between January 1, 1989, and February 28, 1990, written informed consent was obtained from parents to enrol 1456 new-borns. Children were followed up at the ages of 1 (n = 167), 2 (n = 1174), 4 (n = 1218), 10 (n = 1373), and 18 years (n = 1313). Demographic information of parents and offspring, status of allergic diseases, phenotypic measures on allergic sensitization, IgE, and lung function, and environmental exposures, along with other phenotypic measures, were collected at birth and updated at each follow-up.

***Gestational age***

Gestational age was checked and collected from the maternity notes and was used as a continuous variable in the analyses. Preterm birth was defined as gestational age < 37+0 weeks (< 259 days).

***Methylation measurements***

The 102 samples were taken from randomly selected 367 (aged 18) years subjects, we measured DNA methylation from whole blood processed with the Illumina Infinium HumanMethylation450 BeadChip (Illumina Inc., San Diego, USA). CPACOR^56^ pipeline was used for QC and normalisation of the data. Methylation markers on 65 single nucleotide polymorphism (SNP) and sex chromosomes were removed. We applied Illumina background Correction to all intensity values. Any intensity values having detection p-values >= 10^-16^ were set as missing data. Samples with call rate < 98% were excluded. After the QC, 461,230 sites remain for the subsequent analysis. A quantile normalisation was applied using limma on intensity values separately based on six different probe-type categories (Type-I M red, Type-I U red, Type-I M green, Type-I U green, Type-II red, and Type-II green). Beta values were then calculated from these normalised intensity values. Outlier CpGs were removed using 3IQR method after which, 373,936 sites remained for analysis

***Covariates***

Covariates were collected via questionnaires collected at recruitment, before and during pregnancy. Maternal age was derived from mothers’ date of birth. Maternal smoking status and parity were collected from the responses from the questionnaires. Maternal smoking status in pregnancy (Yes/No) was defined as any smoking in pregnancy or no smoking in pregnancy. Socioeconomic status was defined using maternal socioeconomic cluster information (high, low, low-low, low-mid, and mid). Sex of the child and delivery method was obtained from the hospital admin system (patient centres).

***Cell type correction***

Six cell counts (i.e. CD4 T cells, CD8 T cells, NK cells, B cells, monocytes and granulocytes) estimated using the Houseman algorithm^20^ using the minfi package^19^.

***Batch correction***

Indicator of different batches that DNA methylation data were generated were included as a covariate to adjust for batch effect and ComBat^27^ was used to remove any batch effect.

***Exclusion criteria***

## Not applicable

## Isle of Wight Third Generation (IOW F2)

***Design and study population***

This is the Isle of Wight (IOW) 3^rd^ Generation Study^58,59^. The recruitment of newborns started from April 2010. Data used in the analyses were from infants born between April 2010 to May 2014. In total, 200 newborns were recruited such that at least one of their parents is in the IoW birth cohort (IoW F1) and the recruitment is ongoing. Ethics approvals were obtained from the Isle of Wight Local Research Ethics Committee (now named the National Research Ethics Service, NRES Committee

***Gestational age***

Gestational age was checked and collected from the maternity notes and was used as a continuous variable in the analyses. Preterm birth was defined as gestational age < 37+0 weeks (< 259 days).

***Methylation measurements***

The 121 samples were taken from randomly selected from epigenome-wide DNA methylation of 130 newborns using DNA extracted from cord blood. One thousand ng DNA per sample underwent bisulfite conversion using the EZ-96 DNA Methylation kit (Shallow) (Zymo Research Corporation, Irvine, USA). Samples were processed with the Illumina Infinium HumanMethylation450 BeadChip. "CPACOR" method by Lehne et al. 2015^56^ has been used in normalisation the beta values. The 65 single nucleotide polymorphism (SNP) markers were removed. Illumina Background Correction was applied to the intensity values. The CpGs with set intensity values with detection p-value ≥10^-16^ was set as missing and removed in the further analysis. Samples exhibiting call rate <98% were excluded. Quantile normalisation on intensity values was applied by incorporating control probe adjustment and reduction of global correlation. After pre-processing a total 399, 383 CpG sites were remained for subsequent studies.

***Covariates***

Data on covariates were collected via questionnaires collected at recruitment during the 1st trimester of pregnancy, including maternal age, smoking status during pregnancy, parity, and social classes. Information on maternal age was collected by questionnaire at enrolment and it is included in the analysis as a continuous variable. Maternal smoking during pregnancy was assessed by questionnaires at 20 and 28 weeks of pregnancy, and 3 months after birth. Maternal social class was classified on maternal educational level. Sex of the child and delivery method was obtained from the hospital admin system (patient centres).

***Cell type correction***

Seven cell types (B, CD4^+^ T, CD8^+^ T, granulocytes, monocytes, NK and nucleated red blood cells- nRBC) proportions were determined using a cord blood reference panel^12^ using the minfi package^19^.

***Batch correction***

DNA methylation from the 121 subjects were measured in six batches. The R function ComBat^27^ built upon an empirical Bayes framework was used to remove batch effects. Beta-values were calculated for all CpG sites.

***Exclusion criteria***

## Not applicable

## Norwegian Mother and Child Cohort Study (MoBa1, MoBa2 & MoBa3)

***Design and study population***

Participants represent three subsets of mother-offspring pairs from the national Norwegian Mother and Child Cohort Study (MoBa) (1-3)^60^. The years of birth for MoBa participants ranged from 1999-2009. MoBa mothers provided written informed consent. Each subset is referred to here as MoBa1, MoBa2, and MoBa3. MoBa1 is a subset of a larger study within MoBa that included a cohort random sample and cases of asthma at age three years^61^. We previously reported an association between maternal smoking during pregnancy and differential DNA methylation in MoBa1 newborns^62^. We subsequently measured DNA methylation in additional newborns (MoBa2) in the same laboratory (Illumina, San Diego, CA)^14^. MoBa2 included cohort random sample plus cases of asthma at age seven years and nonasthmatic controls. MoBa3 was designed to evaluate the association between differential cord blood DNA methylation and later childhood cancer status. Methylation measurements for MoBa3 were made at the International Agency for Research on Cancer (IARC)^14^. Years of birth were 2002-2004 for children in MoBa1, 2000-2005 for MoBa2, and 2000-2008 for MoBa3. All three studies were approved by the Regional Committee for Ethics in Medical Research, Norway. In addition, MoBa1 and MoBa2 were approved by the Institutional Review Board of the National Institute of Environmental Health Sciences, USA. MoBa1 approved by the regional ethics committee South East B (reference no.: 13419).

***Gestational age***

In MoBa1 gestational age was calculated using ultrasound estimation. If ultrasound was not available, last menstrual period was used (n=964) and MoBa2 gestational age was calculated using ultrasound estimation. If ultrasound was not available, last menstrual period was used (n=609). Gestational age data was collected in MoBa3 based on Medical Birth Record (MBR) as length of gestation in days. Calculated from ultrasound due date (US) n= 21. If no ultrasound, calculated from last menstrual period (LMP) n=2.

***Methylation measurements***

Details of the DNA methylation measurements and quality control for the MoBa1 participants were previously described^63^ and the same protocol was implemented for the MoBa2 participants. Briefly, umbilical cord blood samples were collected and frozen at birth at -80°C. All biological material was obtained from the Biobank of the MoBa study^63^. Bisulfite conversion was performed using the EZ-96 DNA Methylation kit (Zymo Research Corporation, Irvine, CA) and DNA methylation was measured at 485577 CpGs in cord blood using Illumina’s Infinium HumanMethylation450 BeadChip. Raw intensity (.idat) files were handled in R using the minfi package19 to calculate the methylation level at each CpG as the beta-value (β=intensity of the methylated allele (M)/(intensity of the unmethylated allele (U) + intensity of the methylated allele (M) + 100)) and the data was exported for quality control and processing. Probe and sample-specific quality control was performed in the MoBa1, MoBa2, and MoBa3 datasets separately. Similar protocols were applied to MoBa1 and Moba2, as follows: Control probes (N=65) and probes on X (N=11 230) and Y (N=416) chromosomes were excluded in both datasets. Remaining CpGs missing > 10% of methylation data were also removed (N=20 in MoBa1, none in MoBa2). Samples indicated by Illumina to have failed or have an average detection p value across all probes < 0.05 (N=49 MoBa1, N=35 MoBa2) and samples with gender mismatch (N=13 MoBa1, N=8 MoBa2) were also removed. For MoBa1 and MoBa2, we accounted for the two different probe designs by applying the intra-array normalization strategy Beta Mixture Quantile dilation (BMIQ)^30^. After quality control exclusions, the sample sizes were 1,068 for MoBa1 and 685 for MoBa2.

For MoBa3, bisulfite conversion and methylation measurements were done at the International Agency for Research on Cancer (Lyon, France). Similar data quality control and processing was applied with some slight differences. Methylation features were filtered from (i) cross-reactive probes, (ii) probes mapping to sex chromosomes and (iii) probes overlapping with a known single nucleotide polymorphism (SNP) with an allele frequency of at least 5% in the overall population (all ethnic groups), resulting in the exclusion of 36 231 probes. Data quality was further assessed using box plots for the distribution of methylated and unmethylated signals, and multidimensional scaling plots and unsupervised clustering were used to check for sample outliers. After background correction and color-bias adjustment, type I and type II probe distributions were aligned using the intra-array BMIQ from the watermelon package. After quality control, the sample size for MoBa3 was 253.

***Covariates***

For all three datasets, information on child’s sex, maternal age, smoking during pregnancy, education, and parity was collected via questionnaires completed by the mother or from birth registry records as previously described (4). Birth weight, mode of delivery was collected in the Medical Birth Registry. Birth weight, maternal age, and pre-pregnancy BMI were included as continuous variables. Child’s sex and parity were included as dichotomous variables. Maternal smoking status during pregnancy was classified into three groups: non-smoker, stopped smoking in early pregnancy, and smoked throughout pregnancy. Maternal educational level was categorized into four groups based on years of education: less than high school/secondary school, high school/secondary school completion, some college or university, or 4 years of college/university or more. In addition EWAS principal component was included to adjust for ancestry in both MoBa1 and MoBa2 and selection criteria two level categories asthma case/control at age 3 for MoBa1, three level categories asthma case/control and and folate use at age 7 for MoBa2 and two level categories nested case/control study for childhood cancers and year of birth for MoBa3.

The current analyses include the children who had cord blood DNA methylation measurements that passed quality control, birthweight and covariate data (N=964 from MoBa1; N=609 from MoBa2; N=213 from MoBa3). Each dataset was analysed independently.

***Cell type correction***

Seven cell types (B, CD4^+^ T, CD8^+^ T, granulocytes, monocytes, NK and nucleated red blood cells- nRBC) proportions were determined using a cord blood reference panel^12^ using the minfi package^19^.

***Batch correction***

We additionally corrected the analyses for batch effect using ComBat^27^ for MoBa1 and MoBa2 and surrogate variable analysis (SVA) for MoBa3.

***Exclusion criteria***

## For all models we excluded (N=59 for MoBa1, N=37 for MoBa2, and N=19 for MoBa3) infants born more than 42+0 weeks (more than 294 days) or multiple birth cases. In the no complication model whose mothers experienced pre-eclampsia hypertension or diabetes (N=90 for MoBa1, N=76 for MoBa2, and N=23 for MoBa3) during pregnancy or delivery start with induction (N=75 for MoBa1, N=51 for MoBa2, and N=21 for MoBa3) or caesarean section (N=113 for MoBa1, N=77 for MoBa2, and N=9 for MoBa3) were excluded. This information was obtained from questionnaires completed by the mothers shortly after delivery and combined with medical birth records.

## The Northern Finland Birth Cohorts (NFBC1986)

***Design and study population***

The Northern Finland Birth Cohort 1986 consists of 99% of all children, who were born in the provinces of Oulu and Lapland in Northern Finland between 1 July 1985 and 30 June 1986. 9,203 live-born individuals entered the study^64^. At the age of 16, the subjects living in the original target area or in the capital area (n=9,215) were invited to participate in a follow-up study including a clinical examination. 7344 participants attend the study in year 2001/2002, of which 5654 completed the postal questionnaire, the clinical examination and provided a blood sample^65^. DNA was extracted from all 5654 blood samples. An informed consent for the use of the data including DNA was obtained from all subjects. The ethical committee of Northern Ostrobotnia Hospital District has approved the study.

***Gestational age***

Gestational age at birth was self-reported in questionnaires during pregnancy.

***DNA methylation***

DNA methylation was recoded on Illumina HumanMethlation450K array for 566 randomly selected subjects. 24 technical replicates were excluded. 18 samples did not reach a call rate of >95% applying a detection P-value filter of 10^-16^. We excluded 7 samples with gender inconsistency, no sample was outlying from the overall data structure (1^st^ PC score of the DNA methylation values outside mean +/- 4SD). DNA methylation data of 517 samples with 466290 autosomal probes (call rate filter 95%) each were used for this analysis. Methylation of genomic DNA was quantified using the Illumina HumanMethylation450 array according to manufacturer’s instructions. Bisulfite conversion of genomic DNA was performed using the EZ DNA methylation kit according to manufacturer's instructions (Zymo Research, Orange, CA).

***Covariates***

Data on maternal age, parity, socio-economic status, and smoking in pregnancy, age at birth, newborn gender and gestational age at birth were self-reported in questionnaires during pregnancy. New-born birth weight and mode of delivery was recorded by obstetricians or midwives.

***Cell type correction***

Potential confounding effects of blood cell subtypes were estimated by the Houseman method^20^.

***Batch correction***

To account for batch effects in the data, Beta values underwent a functional normalization approach described by Fortin et al^29^ using the first 10 PCs of the Illumina 450K array control probes. This approach includes subset quantile normalization of the data and normal-exponential out-of-band background correction.

***Exclusion criteria***

## For all models we excluded n=9 infants born more than 42+0 weeks (more than 294 days) or multiple birth cases. In the no complication model whose mothers experienced pre-eclampsia hypertension or diabetes (n=122) during pregnancy or delivery start with induction (n=71) or caesarean section (n=75) were excluded. This information was obtained from questionnaires completed by the mothers shortly after delivery and combined with medical birth records.

## Prevention and Incidence of Asthma and Mite Allergy birth cohort (PIAMA)

**Design and Study population**

PIAMA (Prevention and Incidence of Asthma and Mite Allergy) is a birth cohort study of children born in 1996-1997 in the Netherlands. Details of the study design have been published previously^66^. In brief, 10,232 pregnant women completed a validated screening questionnaire at their prenatal health care clinic (n=52). Based on this screening, 7,862 women were invited to participate, of whom 4,146 women agreed and gave informed consent. The study started with 3,963 newborns. Questionnaire based follow-up of the children took place at 3 months of age, yearly from 1 to 8 years of age, and at 11, 14, and 16 years of age, with clinical investigations at ages 4, 8, 12 and 16 years. The Medical Ethical Committees of the participating institutes approved the study, and the parents and legal guardians of all participants as well as the participants themselves gave written informed consent.

**Gestational age**
The variable 'gestational age' is based on self-report. The first question in our pregnancy questionnaire was 'How many weeks are you pregnant now?' and we also asked for the date that this questionnaire was completed. The next questionnaire was sent to the mothers at the child's estimated age of 3 months and in that questionnaire we asked for the date of birth and we also asked for the expected date of birth. The variable for gestational age has been calculated from the difference between these 2 dates.

**Methylation measurements**

In the PIAMA study, DNA from peripheral blood samples was extracted using the QIAamp blood kit (Qiagen or equivalent protocols), followed by precipitation-based concentration using GlycoBlue (Ambion). DNA concentration was determined by Nanodrop measurement and Picogreen quantification. 500 ng of DNA was bisulphite-converted using the EZ 96-DNA methylation kit (Zymo Research), following the manufacturer’s standard protocol. After verification of the bisulphite conversion step using Sanger Sequencing, DNA concentration was normalized, and the samples were randomized to avoid batch effects. The DNA methylation was measured using the Illumina Infinium HumanMethylation450 beadchip. Each chip included one control DNA sample for quality control purposes.

Data preprocessing was performed using the Minfi package^19^. We implemented sample filtering to remove bad quality samples (call rate <99%). Moreover, we used 65 SNP probes to check for concordances between paired DNA samples. Paired samples from the same individuals which show Pearson correlation coefficient <0.9 were regarded as sample mixed ups and were excluded from the study. We further assessed the methylation distribution of the X-chromosome to verify gender. After QC, 217 good quality 4 years and 199 good quality 8 years PIAMA samples were used in the analysis. During processing, the probes on sex chromosomes, the probes that mapped to multiple loci, 65 SNP-probes and the probes containing SNPs at the target CpG sites with a MAF>5% were excluded^4^. We implemented “DASEN”^24^ to perform signal correction and normalization. After quality control, 439306 probes remained for further analysis.

***Covariates***

Maternal age and birth weight were defined as a continuous variable. Maternal social class was defined as the highest attained educational level and coded in three categories: 1=primary school, lower vocational or lower secondary education (low) 2=intermediate vocational education or intermediate/higher secondary education (intermediate) 3= higher vocational education and university (high). Parity was defined as older siblings living in the PIAMA home. Mode of delivery was defined as caesarean section or normal delivery. Maternal smoking during pregnancy was coded as 1= no smoking in pregnancy, 2=smoking but stopped in the first 16 weeks, 3=smoking for longer than 16 weeks.

***Cell type correction***

Cell type correction was determined applied using the reference-based Houseman method^20^ using the minfi package^21^ in the R statistical program^26^ and included relative proportions of six white blood cell subtypes (CD4+ T-lymphocytes, CD8+ T-lymphocytes, NK (natural killer) cells, B-lymphocytes, monocytes and granulocytes), based on a standard reference population.

***Batch correction***

Batch correction was attained including the significant (permutation p-value< 10^-4^) principal components (PCs) derived from the 613 negative control probes^40^ presented in 450K arrays because these negative control probes did not relate to biological variation. After 10000 permutations 5 PCs were retained. The beta-values were batch corrected incorporating these 5 PCs and calculating the residuals of the linear model at 5 years^7^. The covariate one batch was also accounted for in the models, based on the bisulfite treatment.

***Exclusion criteria***

## For all models we excluded (n=4 at age 4 and n=0 at age 8) infants born more than 42+0 weeks (more than 294 days) or multiple birth cases. In the no complication model whose mothers experienced pre-eclampsia hypertension or diabetes (n=23 both 4 and 8 years) during pregnancy or delivery on caesarean section (n=20 at age 4 and n=19 at age 8) were excluded. This information was obtained from questionnaires completed by the mothers shortly after delivery and combined with medical birth records.

## Prediction and Prevention of Preeclampsia and Intrauterine Growth Restriction study (PREDO)

***Design and study population***

The Prediction and Prevention of Preeclampsia and Intrauterine Growth Restriction (PREDO) Study is a longitudinal multicenter pregnancy cohort study of Finnish women and their singleton children born alive between 2006-2010^67^. We recruited 1,079 pregnant women, of whom 969 had one or more and 110 had none of the known clinical risk factors for preeclampsia and intrauterine growth restriction. The recruitment took place when these women attended the first ultrasound screening at 12+0-13+6 weeks+days of gestation in one of the ten hospital maternity clinics participating in the study. The cohort profile^67^contains details of the study design and inclusion criteria. The study protocol was approved by the Ethical Committees of the Helsinki and Uusimaa Hospital District and by the participating hospitals. A written informed consent was obtained from all women.

***Gestational age***

Gestational age was based on ultrasound screening estimation.

***Methylation measurements***

Cord blood samples were run on Illumina 450k Methylation arrays. We randomized all samples on 96-well plates based on gender and maternal risk factors. The quality control pipeline was set up using the R-package *minfi*  (https://www.r-project.org). Three samples were excluded as they were outliers in the median intensities. Furthermore, 20 samples showed discordance between phenotypic sex and estimated sex and were excluded. Nine samples were contaminated with maternal DNA according to the method suggested by Morin et al.^68^ and were also removed. Methylation beta-values were normalized using the *funnorm* function.We excluded any probes on chromosome X or Y, probes containing SNPs and cross-hybridizing probes according to Chen et al.^4^ and Price et al.^69^ Furthermore, any CpGs with a detection p-value > 0.01 in at least 25% of the samples were excluded. The final dataset contained 428,619 CpGs.Association analyses were corrected for infant gender, maternal age at delivery, maternal social class, smoking during pregnancy, parity, mode of delivery with data derived from the Finnish Medical Birth Register, as well as for cell type and the first two principal components derived from genome-wide level genotypes.

***Cell type correction***

Cord blood cell counts were estimated for seven cell types (nucleated red blood cells, granulocytes, monocytes, natural killer cells, B cells, CD4(+)T cells, and CD8(+)T cells) using the method of Bakulski et al.^12^ which is incorporated in the R-package *minfi^19^*.

***Batch correction***

After normalization, two batches, i.e., slide and well, were significantly associated and were removed iteratively using the *Combat* function in the *sva* package^22^.

***Exclusion criteria***

## In the no complication model whose mothers experienced pre-eclampsia, hypertension or diabetes (n=135) during pregnancy, or delivery start with induction (n=198) or caesarean section (n=162) were excluded. This information was obtained from medical records and Finnish Medical Birth Register.

## Project Viva

***Design and study population***

Project Viva is a longitudinal pre-birth cohort. We recruited pregnant women at their initial prenatal visit at Atrius Harvard Vanguard Medical Associates, a large multispecialty group practice in eastern Massachusetts, between 1999 and 2002. Exclusion criteria included multiple gestation, inability to answer questions in English, gestational age ≥22 weeks at recruitment and plans to move away before delivery. Mothers provided written informed consent at study recruitment and at every follow-up visit. All study protocols were reviewed and carried out in accordance with guidelines approved by the human subjects committee of Harvard Pilgrim Health Care. Additional details of the cohort have been published elsewhere^70^. Of 2128 live births, 485 participants had genetic consent and cord blood DNA methylation data and 460 had genetic consent and mid-childhood (mean 7.8 years) DNA methylation data.

***Gestational age***

We calculated length of gestation in days by subtracting the date of the LMP from the date of delivery. If gestational age according to the 2^nd^ trimester ultrasound differed from that according to the LMP by >10 days, we used the ultrasound result to determine gestational duration.

***DNA Extraction and Sample Collection***

Trained medical personnel obtained umbilical cord blood samples immediately upon delivery storing them in a dedicated refrigerator at 4 °C and transported to a central location within 24 hours of sample collection. Similarly, whole blood samples collected during mid-childhood were stored at 4 °C and transported to the central storage location for sample processing. Subsequently, trained laboratory staff processed the samples on the same day of arrival, and DNA was extracted using the Qiagen Puregene Kit (Valencia, CA). Aliquots were then stored at −80 °C until analysis.

***DNA methylation Assessment and Quality Control***

Buffy coat DNA was sodium bisulfite converted using the EZ DNA Methylation-Gold Kit (Zymo Research, Irvine, CA). Samples were allocated to plates using a two-stage algorithm by randomizing 12 samples to each chip and then randomly assigning eight chips to each of the 15 plates used to ensure balance by sex across chips and plates. Samples were shipped to Illumina Inc. and analyzed using the Infinium Human Methylation450 BeadChip (Illumina, San Diego, CA) following standard manufacturer’s protocols. The Human Methylation450 BeadChip measures DNA methylation at >485,000 CpG sites simultaneously at a single nucleotide resolution, covering 99% of the RefSeq genes.

We processed raw methylation image files using the *minfi* package in R^19^. Samples were excluded as potentially miss-labelled if they were mismatches on sex (n = 6), genotype (n = 6) or were deemed to be low in quality (n = 12). Technical replicates were also excluded from the analysis (n = 40). Correlation coefficients for individual probes among all technical replicates ranged from 0.98 to 1. We excluded individual probes if they had non-significant detection *P*-values (*P* > 0.05) for more than 1% of the samples. Additionally, non-CpG probes (i.e. rs and ch), probes in X and Y chromosomes, SNP-associated probes at either the single base extension or within the target region were removed for SNPs that have a minor-allele frequency of >5%. Previously identified non-specific and cross-reactive probes within the array along with polymorphic CpG loci were also excluded from the analysis^30^. Background correction and dye-bias equalization was performed via the normal-exponential out-of-band (*noob*) correction method^36^. Finally, a β-mixture quantile intra sample normalization procedure (BMIQ) was applied to the resulting data to reduce the potential bias that can arise from

type2 probes^30^.

***Covariates***

Using a combination of self-administered questionnaires and interviews, we collected information about maternal age (continuous), education (≥ college graduate yes v. no), smoking status (any smoking during pregnancy yes v. no), and parity (0 v. ≥ 1) and child’s age (continuous) and sex (male v. female). From medical records, we collected birth weight and mode of delivery.

***Cell type adjustment***

White blood cell composition was estimated from DNA methylation measurements using the Houseman projection method^20^ from isolated cell types. To estimate cell types composition in cord blood we used a reference panel of nucleated cells isolated from cord blood (leukocytes and nucleated red blood cells)^12^ and an adult leukocyte reference panel for blood samples collected in mid-childhood as implemented in *minfi^19,25^*.

***Batch correction***

We used ComBat^27^ to correct for batch effects from plate and other potential sources of technical variability in methylation measurements, while protecting gestational age in the model statement of ComBat.

**Exclusion criteria**

We excluded participants who were non-white, had pre-eclampsia, gestational hypertension, or gestational diabetes during the index pregnancy, and those missing covariate data. Based on these exclusions, of 485 participants with genetic consent and cord blood DNA methylation data, we included 317 in the newborn analysis sample. Of 460 had genetic consent and mid-childhood DNA methylation data, we included 276 in the school age analysis sample.

## The West Australian Pregnancy Cohort (Raine)

***Design and study population***

Participants were from the Western Australian Pregnancy Cohort (Raine) Study^71^. Pregnant women (n=2900) were recruited through the public antenatal clinic at King Edward Memorial Hospital and nearby private clinics in Perth, Western Australia between May 1989 and November 1991. Total of 2868 newborns were available for follow-up. The King Edward Memorial Hospital and Princess Margaret Hospital Ethic Committees approved the study protocol. The participant and/or their primary caregiver provided written consent for their participation in the study. All measurements were performed by research personnel trained accordingly.

***Gestational age***

Gestational age was based on the date of the last menstrual period unless there was discordance of more than seven days with ultrasound measurements <18-weeks; in those cases, the estimate was based on ultrasound biometry at 18-weeks’ gestation.

***Methylation measurements***

For this methylation study we used data from the 17 years follow-up using DNA from whole blood. Epigenome-wide DNA methylation profiles of 1192 (58 technical replicates) individuals were examined using the Illumina Infinium HumanMethylation450 BeadChip array (Illumina San Diego, CA) and was done at the Centre for Molecular Medicine and Therapeutics (http://www.cmmt.ubc.ca). Quality control of the samples was done using the R statistical packages; shinyMethyl^72^ and MethylAid^55^. Three samples were outliers based on these two packages Gender was inferred using the RnBeads R package^73^ and identified a discrepancy for a single sample. Fifty-eight of the samples were run in duplicate or triplicate and 65 SNPs present on the array were used to assess genetic similarity between individuals and one contaminated sample was excluded Intentional SNP CpGs (n=65), sex chromosome CpG (n=11,648) and CpGs with a detection p-value > 0.05 in any sample (n=10,777) were removed. A further 160 probes with low bead counts (bead counts less than 3 in more than 5% of samples) were removed. Principal component analysis was performed on the top 20,000 most variable probes and permutation tests were used to test for association between the top 10 principal components and experimental variables. Beta-mixture quantile normalisation (BMIQ) was applied^30^ to each CpG and batch effects were still present, therefore were accounted for in all models across a total of 462,297 CpGs.

***Covariates***

Data on maternal age, educational level and parity and maternal smoking during pregnancy were assessed by questionnaires at 18- and 34-weeks pregnancy. Maternal age at delivery was derived from the mother’s report of her own and her baby’s dates of birth. Mode of delivery was placed into four categories; 1) vaginal delivery spontaneous; 2) vaginal delivery induced; 3) cesarean section delivery, elective; and 4) cesarean section, urgent. Birth weight was measured in grams at birth age. Age from the 17 years follow-up and sex was used as a covariate in all models. The first five principal components from genotype data were used to account for ancestry. After accounting for missing data there were a total 669 participants available for analysis.

***Cell type correction***

Cell type correction was determined applied using the reference-based Houseman method^20^ using the minfi package^21^ in the R statistical program^26^ and included relative proportions of six white blood cell subtypes (CD4+ T-lymphocytes, CD8+ T-lymphocytes, NK (natural killer) cells, B-lymphocytes, monocytes and granulocytes), based on a standard reference population.

***Batch correction***

Batch was corrected for by including sentrix plate ID, plate position, and pool ID in all statistical models.

***Exclusion criteria***

For all models we excluded n=57 infants born more than 42+0 weeks (more than 294 days) or multiple birth cases. In the no complication model whose mothers experienced pre-eclampsia hypertension or diabetes (n=154) during pregnancy or delivery start with induction (n=110) or caesarean section (n=148) were excluded.

# Fetal lung methods

***Design and study population***

These fetal lung data primary study has been described in Chhabra D et al.^8^. De-identified fetal lung tissue samples were acquired through the tissue retrieval program sponsored by the National Institute of Child Health and Development, the University of Maryland Brain and Tissue Bank for Developmental Disorders (Baltimore, MD), and the Center for Birth Defects Research (University of Washington, Seattle, WA). Phenotypic information for the fetal lung tissue samples is limited to their estimated gestational age, sex and a history of cigarette smoking as reported by the mother that was obtained at the time of tissue collection. Therefore, underlying pathologic conditions in the fetus are unknown. The use of these tissues was declared non–human subject research by the University of Missouri-Kansas City Pediatric Health Sciences Review Board.

***Methylation measurements***

This data is not publicly available, and the primary study has been described in Chhabra D et al.^8^. Lung tissues were flash frozen at the time of procurement and stored at −80ºC. Lung tissue DNA were quantified using standard pico-green methods. Genome-wide methylation assay was performed with 750 ng of bisulfite-treated DNA per sample using the Illumina IInfinium Human Methylation 450K BeadChip and aligned to reference genome hg19, according to manufacturer's recommended protocol. Results were extracted using GenomeStudio (Software v2011.1) and then read into Bioconductor (version 2.12) for further analyses. Percent methylation was reported as the Illumina β-value, which is the ratio between methylated signal intensity and total probe signal intensity quality control steps performed for DNA methylation marks have been described^8^. For this particular study, we investigated lung tissue of 74 fetal subjects from estimated ages 59 to 122 post conception, 35/39 (non IUS/IUS, in utero smoke exposed), 28/46 (female/male). We considered 349,355 CpGs that passed quality control.

***Covariates***

The potential biological covariates sex and IUS were adjusted for both gene expression and methylation data set.

***Batch correction***

We did not observe technical batch effects for either the methylation or gene expression data sets.

#

# Cohort-specific acknowledgements (alphabetical order)

## ALSPAC

We are extremely grateful to all the families who took part in this study, the midwives for their help in recruiting them, and the whole ALSPAC team, which includes interviewers, computer and laboratory technicians, clerical workers, research scientists, volunteers, managers, receptionists, and nurses. We would like to acknowledge Tom Gaunt, Oliver Lyttleton, Sue Ring, Nabila Kazmi, and Geoff Woodward for their earlier contribution to the generation of ARIES data (ALSPAC methylation data).

## BAMSE

We would like to thank all participating BAMSE children and parents for the engagement in our study and Ingrid Delin for technical assistance.

## CBC

We are grateful for the efforts of Marty Kharrazi, Robin Cooley, and Steve Graham from the California Department of Health Services, Genetic Diseases Branch.

## CHAMACOS

We are grateful to CHAMACOS participants, researchers, and field staff. Contributions of the personnel and students in Holland and Barcellos laboratories are acknowledged.

## CHS

We are indebted to the school principals, teachers, students and parents in each of the study communities for their cooperation and especially to the members of the health testing field team for their efforts. We would like to express our sincere gratitude to Steve Graham and Robin Cooley at the California Biobank Program and Genetic Disease Screening Program within the California Department of Public Health for their assistance and advice regarding newborn bloodspots. The biospecimens and/or data used in this study were obtained from the California Biobank Program, (SIS request number(s) 479)” Section 6555(b), 17 CCR.  The California Department of Public Health is not responsible for the results or conclusions drawn by the authors of this publication.

## EDEN

## We acknowledge the participating families, midwife research assistants for data collection.

## EXPOsOMICS: RHEA/ENVIRONAGE/Piccolipiù

## We thank all of the participants of the Rhea study and the interviewers, statisticians and the hospital personnel for their cooperation and contribution to this study.

## We are extremely grateful to the women and neonates participating in ENVIRONAGE, as well as the staff of the maternity ward, the midwives, and the staff of the clinical laboratory of East-Limburg Hospital in Genk.

## Our thanks go to all the families who took part in the Piccolipiù study, to the midwives for their help in recruiting them, and to the whole Piccolipiù team, which includes doctors, nurses, research scientists, and computer/laboratory technicians.

The authors would also like to acknowledge Paolo Vineis for his input and coordination of the EXPOsOMICS project. We thank Mr. Cyrille Cuenin for his help and dedication in the methylome experimental workflow, Mr. Vincent Cahais for his help in data management and the Genetic Cancer Susceptibility Group at IARC for their assistance in these experiments.

## GECKO

## We are grateful to the families who took part in the GECKO Drenthe birth cohort, the midwives, gyneacologists, nurses and GPs for their help for recruitment and measurement of participants, and the whole team from the GECKO Drenthe study.

## Gen3G

Gen3G investigators acknowledge the Blood sampling in pregnancy clinic at the Centre Hospitalier de l'Universite de Sherbrooke (CHUS), and the assistance of clinical research nurses for recruiting women and obtaining consent for the study at the Research Center of CHUS. They also thank the CHUS Research in obstetrics services for organization of biosamples collection at delivery.

## Generation R

The Generation R Study is conducted by Erasmus MC in close collaboration with the School of Law and Faculty of Social Sciences of the Erasmus University Rotterdam, the Municipal Health Service Rotterdam area, Rotterdam, the Rotterdam Homecare Foundation, Rotterdam and the Stichting Trombosedienst & Artsenlaboratorium Rijnmond (STAR-MDC), Rotterdam. We gratefully acknowledge the contribution of children and parents, general practitioners, hospitals, midwives and pharmacies in Rotterdam. The study protocol was approved by the Medical Ethical Committee of the Erasmus Medical Centre, Rotterdam. Written informed consent was obtained for all participants. The generation and management of the Illumina 450K methylation array data (EWAS data) for the Generation R Study was executed by the Human Genotyping Facility of the Genetic Laboratory of the Department of Internal Medicine, Erasmus MC, the Netherlands. We thank Mr. Michael Verbiest, Ms. Mila Jhamai, Ms. Sarah Higgins, Mr. Marijn Verkerk and Dr. Lisette Stolk for their help in creating the EWAS database.

## GOYA

The authors want to thank the many pregnant women who have taken part in the study and the GPs and midwives who have engaged themselves in the project. Without their help, there would be no cohort.

## INMA

INMA researchers would like to thank all the participants for their generous collaboration and grateful to Silvia Fochs, Nuria Pey, and Muriel Ferrer for their assistance in contacting the families and administering the questionnaires. A full roster of the INMA Project Investigators can be found at <http://www.proyectoinma.org/presentacion-inmapresentacioninma/listado-investigadores/en_listado-investigadores.html>.

## IOW F1

The authors gratefully acknowledge the cooperation of the children and parents who participated in this study and appreciate the hard work of the Isle of Wight research team in collecting data and of Nikki Graham for technical support.

## IOW F2

We are sincerely thankful to all the families who took part in this study, the nurses for their help in recruiting them, and the whole IOW team. In particular, we would like to thank Stephen Porter, Sharon Matthews, Frances Mitchell and Nikki Graham for technical support.

**MoBa1 and MoBa2**

The Norwegian Mother and Child Cohort Study are supported by the Norwegian Ministry of Health and Care Services and the Ministry of Education and Research, NIH/NIEHS (contract no N01-ES-75558), NIH/NINDS (grant no.1 UO1 NS 047537-01 and grant no.2 UO1 NS 047537-06A1). For this work, MoBa 1 and 2 were supported by the Intramural Research Program of the NIH, National Institute of Environmental Health Sciences (Z01-ES-49019) and the Norwegian Research Council/BIOBANK (grant no 221097), and through its Centres of Excellence funding scheme, project number 262700. We are grateful to all the participating families in Norway who take part in this on-going cohort study.

## MoBa3

We are grateful to all the participating families in Norway who take part in this on-going cohort study, and to the International Childhood Cancer Cohort Consortium (I4C). We thank Mr. Cyrille Cuenin for his help and dedication in the methylome experimental workflow, Mr. Vincent Cahais for his help in data management and the Genetic Cancer Susceptibility Group at IARC for their assistance in these experiments.

## NFBC1986

We gratefully acknowledge the contributions of the participants in the Northern Finland Birth Cohort 1986. We also thank all the field workers and laboratory personnel for their efforts.

## PIAMA

We thank all participating families in the Piama birth cohort, as well as the field and technical workers who assisted in collection and processing of the samples.

## PREDO

We thank all mothers who took part in the on-going PREDO study.

## Project Viva

We are indebted to the Project Viva mothers, children and families for their ongoing participation.

## Raine

The authors are grateful to the Raine Study participants and their families, and the Raine Study management team for cohort co-ordination and data collection, the National Health & Medical Research Council (NHMRC) for their long term contribution to funding the study over the last 20 years and The Telethon Kids Institute for long term support of the Study. We also acknowledge The University of Western Australia (UWA), Raine Medical Research Foundation, The Telethon Kids Institute, UWA Faculty of Medicine, Dentistry and Health Sciences, Women and Infants Research Foundation and Curtin University for providing funding for Core Management of the Raine Study. The DNA methylation work was supported by NHMRC grant 1059711. Collaborative analyses are supported by NHMRC 1142858. Data collection and biological specimens at the 17 years follow-up were funded by the NHMRC Program Grant ID 353514 and Project Grant #403981. RCH is supported by NHMRC Fellowship grant number 1053384. This work was supported by resources provided by The Pawsey Supercomputing Centre with funding from the Australian Government and the Government of Western Australia.

# Cohort-specific funding statements (alphabetical order)

## ALSPAC

The UK Medical Research Council (MRC) and the Wellcome Trust (Grant ref: 102215/2/13/2) and the University of Bristol provide core support for ALSPAC. The Accessible Resource for Integrated Epigenomics Studies (ARIES) which generated large scale methylation data was funded by the UK Biotechnology and Biological Sciences Research Council (BB/I025751/1 and BB/I025263/1). Additional epigenetic profiling on the ALSPAC cohort was supported by the UK Medical Research Council Integrative Epidemiology Unit and the University of Bristol (MC_UU_12013 and MC_UU_0011) the Wellcome Trust (WT088806) and the United States National Institute of Diabetes and Digestive and Kidney Diseases (R01 DK10324). DAL's contribution to this work is supported by grants from the US NIH [[R01 DK1034](https://www.sciencedirect.com/science/article/pii/S0277953618303927#gs2)] and the European Union's Seventh Framework Programme[[FP/2007-2013](https://www.sciencedirect.com/science/article/pii/S0277953618303927#gs3))/ERC Grant Agreement (Grant number [66945](https://www.sciencedirect.com/science/article/pii/S0277953618303927#gs4); DevelopObese)]. DAL is a National Institute of Health Research Senior Investigator [[NF-SI-0611-10196](https://www.sciencedirect.com/science/article/pii/S0277953618303927#gs5)]. The funders had no role in study design, data collection and analysis, decision to publish, or preparation of the manuscript. This publication is the work of the authors and GEMMA SHARP, DEBBIE LAWLOR and CAROLINE RELTON will serve as guarantors for the contents of this paper.

## BAMSE

BAMSE was supported by The Swedish Research Council, The Swedish Heart-Lung Foundation, MeDALL (Mechanisms of the Development of ALLergy) a collaborative project conducted within the European Union (grant agreement No. 261357), Stockholm County Council (ALF), Cancer- och Allergifonden, Swedish foundation for strategic research (SSF) (RBc08-0027), the Strategic Research Programme (SFO) in Epidemiology at Karolinska Institutet and the Swedish Research Council Formas. EM is supported by grants from the Swedish Research Council the Strategic Research Area Epidemiology at Karolinska Institutet and the European Research Council (ERC; grant agreement n° 757919, TRIBAL).

## CBC

This work was supported by the US National Institute of Environmental Health Sciences and the Environmental Protection Agency (P01ES018172); The National Institute of Environmental Health Sciences (R01ES09137); The US National Cancer Institute (grant number: R01CA155461); and the US National Cancer Institute Cancer Center Support Grant (5P30CA082103).

## CHAMACOS

This research was supported by grants of the National Institute of Environmental Health Science (NIEHS) [P01 ESO09605, R01ES023067, R01ES012503, R01ES021369, R24ES028529]; Environmental Protection Agency (RD83273401, R283171001); and the National Institutes of Health (NIH) (UG3OD023356).

## CHS

This work was supported by NIEHS grants K01ES017801, R01ES022216, and P30ES007048.

## EDEN

We acknowledge all funding sources for the EDEN study: Foundation for Medical Research (FRM), National Agency for Research (ANR), National Institute for Research in Public Health (IRESP: TGIR cohorte santé 2008 program), French Ministry of Health (DGS), French Ministry of Research, Inserm Bone and Joint Diseases National Research (PRO-A) and Human Nutrition National Research Programs, Paris–Sud University, Nestlé, French National Institute for Population Health Surveillance (InVS), French National Institute for Health Education (INPES), the European Union FP7 programmes (FP7/2007-2013, HELIX, ESCAPE, ENRIECO, MeDALL projects), Diabetes National Research Program (through a collaboration with the French Association of Diabetic Patients (AFD)), French Agency for Environmental Health Safety (now ANSES), Mutuelle Générale de l’Education Nationale (MGEN), French National Agency for Food Security, the EU FP7-ENV funded Health and Environment-wide Associations based on Large population Surveys (HEALS) project (N: 603946) and the French-speaking association for the study of diabetes and metabolism (ALFEDIAM). The funders had no role in study design, data collection and analysis, decision to publish, or preparation of the manuscript.

## EXPOsOMICS: RHEA/ENVIRONAGE/Piccolipiù

## The Rhea project was financially supported by European Union (EU) grants for specific projects (EU FP6-2003-Food-3-NewGeneris; EU FP6. STREP HiWATE; EU FP7 ENV.2007.1.2.2.2. Project no. 211250 ESCAPE; EU FP7-2008-ENV-1.2.1.4 Envirogenomarkers; EU FP7-HEALTH-2009-single stage CHICOS; EU FP7 ENV.2008.1.2.1.6. Proposal no. 226285 ENRIECO; EU-FP7-HEALTH-2012 Proposal no. 308333 HELIX), MeDALL (FP7 European Union project, no. 264357), and the Greek Ministry of Health (Program of Prevention of obesity and neurodevelopmental disorders in preschool children, in Heraklion district, Crete, Greece: 2011–2014; “Rhea Plus”: Primary Prevention Program of Environmental Risk Factors for Reproductive Health, and Child Health: 2012–15).

## The ENVIRONAGE birth cohort is supported by the European Research Council [ERC-2012-StG.310898] and by funds from the Flemish Scientific Research council [FWO, G.0.733.15.N].

## Piccolipiù was approved and initially funded by the Italian National Centre for Disease Prevention and Control (CCM grant 2010) and by the Italian Ministry of Health (art 12 and 12bis Dl.gs.vo 502/92).

The methylation assays of the three cohorts were funded by the European Community's Seventh Framework Programme FP7/2007–2013 project EXPOsOMICS (grant no. 308610). ZH and AG and the Epigenetics Group at IARC are supported by grants from the Institut National du Cancer (INCa, Plan Cancer-EVA-INSERM, France).

## GECKO

## The GECKO Drenthe birth cohort was funded by an unrestricted grant of Hutchison Whampoa Ld, Hong Kong and supported by the University of Groningen, Well Baby Clinic Foundation Icare, Noordlease and Youth Health Care Drenthe. This methylation project in the GECKO Drenthe cohort was supported by the Biobanking and Biomolecular Research Infrastructure Netherlands (CP2011-19). This project received funding from the European Union’s Horizon 2020 research and innovation programme (733206, LIFECYCLE).

## Gen3G

Gen3G was supported by Fonds de la recherche du Québec en santé (FRSQ) operation grant #20697 (M.F.H); a Canadian Institute of Health Research (CIHR) grant #MOP 115071 (M.F.H), and Diabète Québec grants (P.P. and L.B.). M.F.H. is supported by American Diabetes Association (ADA) Pathways to Stop Diabetes award (1-15-ACE-26).

## Generation R

The general design of the Generation R Study is made possible by financial support from the Erasmus Medical Center, Rotterdam, the Erasmus University Rotterdam, the Netherlands Organization for Health Research and Development and the Ministry of Health, Welfare and Sport. The EWAS data was funded by a grant to VWJ from the Netherlands Genomics Initiative (NGI)/Netherlands Organisation for Scientific Research (NWO) Netherlands Consortium for Healthy Aging (NCHA; project nr. 050-060-810), by funds from the Genetic Laboratory of the Department of Internal Medicine, Erasmus MC, and by a grant from the National Institute of Child and Human Development (R01HD068437). VWJ received an additional grant from the Netherlands Organization for Health Research and Development (VIDI 016.136.361) and a Consolidator Grant from the European Research Council (ERC-2014-CoG-648916). This project received funding from the European Union’s Horizon 2020 research and innovation programme (633595, DynaHEALTH and 733206, LifeCycle). LD received funding from the co-funded programme ERA-Net on Biomarkers for Nutrition and Health (ERA HDHL) (ALPHABET project, Horizon 2020 (grant agreement no 696295; 2017), ZonMw the Netherlands (no 529051014; 2017)). JFF received funding from the Joint Programming Initiative a Healthy Diet for a Healthy Life (JPI HDHL) (NutriPROGRAM, ZonMw the Netherlands, project no.529051022).

## GOYA

Genotyping for the GOYA Study was funded by the Wellcome Trust (grant ref: 084762MA). Generation of DNA methylation data was funded by the MRC Integrative Epidemiology Unit which is supported by the Medical Research Council (MC_UU_12013 and MC_UU_0011) and the University of Bristol.

## INMA

Main funding of the epigenetic studies in INMA was funded by grants from Instituto de Salud Carlos III (Red INMA G03/176, CB06/02/0041), Spanish Ministry of Health (FIS-PI041436PI04/1436, FIS-PI081151), PI08/1151 including FEDER funds, FIS-PI11/00610, FIS-FEDER-PI06/0867, FIS-FEDER-PI03-1615) Generalitat de Catalunya-CIRIT 1999SGR 00241, Fundació La marató de TV3 (090430), EU Commission (261357-MeDALL: Mechanisms of the Development of ALLergy). This project received funding from the European Union’s Horizon 2020 research and innovation programme (733206, LIFECYCLE). LAS was supported through a Colciencias PhD Scholarship, Colombia (Grant: 529/2011). Research Council (268479-BREATHE: BRain dEvelopment and Air polluTion ultrafine particles in school children).

## IOW F1

This work has been supported by National Institute of Health R01 AI091905 and R01HL132321, R01 AI121226, R01 HL082925; National Asthma Campaign, UK (Grant No 364). The content is solely the responsibility of the authors and does not necessarily represent the official views of the National Institutes of Health, USA.

## IOW F2

The third-generation study has been supported by National Institute of Health R01 AI091905 (and R01 HL082925. The content is solely the responsibility of the authors and does not necessarily represent the official views of the National Institutes of Health, USA.

## MoBa1, MoBa2 and MoBa3

The Norwegian Mother and Child Cohort Study are supported by the Norwegian Ministry of Health and Care Services and the Ministry of Education and Research, NIH/NIEHS (contract no N01-ES-75558), NIH/NINDS (grant no.1 UO1 NS 047537-01 and grant no.2 UO1 NS 047537-06A1). For this work, MoBa 1 and 2 were supported by the Intramural Research Program of the NIH, National Institute of Environmental Health Sciences (Z01-ES-49019) and the Norwegian Research Council/BIOBANK (grant no 221097). The work was also partly funded by the Norwegian Research Council’s Centre of Excellence funding scheme (project no. 262700). The work in MoBa3 was supported in part by a Postdoctoral Fellowship grant from the Ullevål Hospitals Research Council (now under Oslo University Hospital) and travel grants from the Unger -Vetlesens foundation and the Norwegian American Womens Club, all to MCMK. MoBa3 methylation sample retrieval was funded by INCA/INSERM-Plan Cancer, France, and the International Childhood Cancer Cohort Consortium (I4C). The work performed by the Epigenetics Group at the International Agency for Research on Cancer (IARC, Lyon, France), ZH and AG were supported by the grant from Institut National du Cancer (INCa) / INSERM-Plan Cancer (France, 2015), and Association pour la Recherche sur le Cancer (ARC, France). AG was also supported by the IARC Postdoctoral Fellowship, partially supported by the EC FP7 Marie Curie Actions-People-Co-funding of regional, national and international programmes (COFUND).

## NFBC1986

MW was supported by the European Union’s Horizon 2020 research and innovation programme under grant agreement No 633212. NFBC1986 received financial support from EU QLG1-CT-2000-01643 (EUROBLCS) Grant no. E51560, NorFA Grant no. 731, 20056, 30167, USA / NIHH 2000 G DF682 Grant no. 50945 and H2020-633595 DynaHEALTH action and academy of Finland EGEA-project (285547).

## PIAMA

The Piama birth cohort was funded by grants from the Dutch Lung Foundation, European Commission's Seventh Framework Programme (grant number 261357); Netherlands Organization for Health Research and Development; Netherlands Organization for Scientific Research; Netherlands Ministry of Spatial Planning, Housing, and the Environment; Netherlands Ministry of Health, Welfare, and Sport;

## PREDO

This work was supported by the Academy of Finland; European Commission (Horizon 2020 Award SC1-2016-RTD-733280 RECAP); Norface Dial (PremLife); Foundation for Pediatric Research; Juho Vainio Foundation; Novo Nordisk Foundation; Signe and Ane Gyllenberg Foundation; Sigrid Jusélius Foundation; Finnish Medical Foundation; Jane and Aatos Erkko Foundation; Päivikki and Sakari Sohlberg Foundation. Yrjö Jahnsson foundation and Jalmari and Rauha Ahokas foundation.

## Project Viva

R01 HL111108, R01 NR013945, R01 HD034568, UH3 OD023286

## Raine

The authors acknowledge the contributions to core funding of the Raine Study by the University of Western Australia, the Telethon Kids Institute, the Raine Medical Research Foundation, the Faculty of Medicine, Dentistry and Health Science (UWA), the Women and Infants Research Foundation, Curtin University, and Edith Cowan University. The authors also acknowledge the long-term support of the National Health and Medical Research Council of Australia. The epigenetic data collection is supported by NHMRC grant #1059711. Rae-Chi Huang supported by NHMRC fellowships 1053384. This work was supported by resources provided by The Pawsey Supercomputing Centre with funding from the Australian Government and the Government of Western Australia.

## Fetal lung project

P01 HL 132825 and R21 HL 107927

**References**

1. Bohlin, J.*, et al.* Prediction of gestational age based on genome-wide differentially methylated regions. *Genome biology* **17**, 207 (2016).

2. Simpkin, A.J.*, et al.* Longitudinal analysis of DNA methylation associated with birth weight and gestational age. *Human molecular genetics* **24**, 3752-3763 (2015).

3. Naeem, H.*, et al.* Reducing the risk of false discovery enabling identification of biologically significant genome-wide methylation status using the HumanMethylation450 array. *BMC genomics* **15**, 51 (2014).

4. Chen, Y.A.*, et al.* Discovery of cross-reactive probes and polymorphic CpGs in the Illumina Infinium HumanMethylation450 microarray. *Epigenetics* **8**, 203-209 (2013).

5. Hartigan, J.A.H.P.M. The Dip Test of Unimodality. *The Annals of Statistics* **13**, 70-84 (1985).

6. Maechler, M. Hartigan's Dip Test Statistics for Unimodality-Corrected. R package. (2015).

7. Xu, C.-J.*, et al.* The emerging landscape of dynamic DNA methylation in early childhood. *BMC genomics* **18**, 25 (2017).

8. Chhabra, D.*, et al.* Fetal lung and placental methylation is associated with in utero nicotine exposure. *Epigenetics* **9**, 1473-1484 (2014).

9. Spiers, H.*, et al.* Methylomic trajectories across human fetal brain development. *Genome research* **25**, 338-352 (2015).

10. Court, F.*, et al.* Genome-wide parent-of-origin DNA methylation analysis reveals the intricacies of human imprinting and suggests a germline methylation-independent mechanism of establishment. *Genome research* **24**, 554-569 (2014).

11. Flanagan, J.M.*, et al.* Temporal stability and determinants of white blood cell DNA methylation in the breakthrough generations study. *Cancer epidemiology, biomarkers & prevention : a publication of the American Association for Cancer Research, cosponsored by the American Society of Preventive Oncology* **24**, 221-229 (2015).

12. Bakulski, K.M.*, et al.* DNA methylation of cord blood cell types: Applications for mixed cell birth studies. *Epigenetics* **11**, 354-362 (2016).

13. Triche, T. FDb.InfiniumMethylation.hg19: Annotation package for Illumina Infinium DNA methylation probes. R package version 2.2.0. . (2014).

14. Joubert, B.R.*, et al.* DNA Methylation in Newborns and Maternal Smoking in Pregnancy: Genome-wide Consortium Meta-analysis. *American journal of human genetics* **98**, 680-696 (2016).

15. Pinto, J.P.*, et al.* StemChecker: a web-based tool to discover and explore stemness signatures in gene sets. *Nucleic acids research* **43**, W72-77 (2015).

16. Boyd, A.*, et al.* Cohort Profile: the 'children of the 90s'--the index offspring of the Avon Longitudinal Study of Parents and Children. *International journal of epidemiology* **42**, 111-127 (2013).

17. Fraser, A.*, et al.* Cohort Profile: the Avon Longitudinal Study of Parents and Children: ALSPAC mothers cohort. *International journal of epidemiology* **42**, 97-110 (2013).

18. Relton, C.L.*, et al.* Data Resource Profile: Accessible Resource for Integrated Epigenomic Studies (ARIES). *International journal of epidemiology* **44**, 1181-1190 (2015).

19. Aryee, M.J.*, et al.* Minfi: a flexible and comprehensive Bioconductor package for the analysis of Infinium DNA methylation microarrays. *Bioinformatics (Oxford, England)* **30**, 1363-1369 (2014).

20. Houseman, E.A.*, et al.* DNA methylation arrays as surrogate measures of cell mixture distribution. *BMC bioinformatics* **13**, 86 (2012).

21. Jaffe, A.E. & Irizarry, R.A. Accounting for cellular heterogeneity is critical in epigenome-wide association studies. *Genome biology* **15**, R31 (2014).

22. Leek, J.T., Johnson, W.E., Parker, H.S., Jaffe, A.E. & Storey, J.D. The sva package for removing batch effects and other unwanted variation in high-throughput experiments. *Bioinformatics (Oxford, England)* **28**, 882-883 (2012).

23. Wickman, M., Kull, I., Pershagen, G. & Nordvall, S.L. The BAMSE project: presentation of a prospective longitudinal birth cohort study. *Pediatric allergy and immunology : official publication of the European Society of Pediatric Allergy and Immunology* **13 Suppl 15**, 11-13 (2002).

24. Pidsley, R.*, et al.* A data-driven approach to preprocessing Illumina 450K methylation array data. *BMC genomics* **14**, 293 (2013).

25. Reinius, L.E.*, et al.* Differential DNA methylation in purified human blood cells: implications for cell lineage and studies on disease susceptibility. *PloS one* **7**, e41361 (2012).

26. Team, R.C. A language and environment for statistical computing. *R Foundation for Statistical Computing, Vienna, Austria. ISBN 3-900051-07-0, URL* [*http://www.R-project.org/*](http://www.R-project.org/)*.* (2013).

27. Johnson, W.E., Li, C. & Rabinovic, A. Adjusting batch effects in microarray expression data using empirical Bayes methods. *Biostatistics (Oxford, England)* **8**, 118-127 (2007).

28. Ma, X.*, et al.* Ethnic difference in daycare attendance, early infections, and risk of childhood acute lymphoblastic leukemia. *Cancer epidemiology, biomarkers & prevention : a publication of the American Association for Cancer Research, cosponsored by the American Society of Preventive Oncology* **14**, 1928-1934 (2005).

29. Fortin, J.P.*, et al.* Functional normalization of 450k methylation array data improves replication in large cancer studies. *Genome biology* **15**, 503 (2014).

30. Teschendorff, A.E.*, et al.* A beta-mixture quantile normalization method for correcting probe design bias in Illumina Infinium 450 k DNA methylation data. *Bioinformatics (Oxford, England)* **29**, 189-196 (2013).

31. Joo, J.E.*, et al.* The use of DNA from archival dried blood spots with the Infinium HumanMethylation450 array. *BMC biotechnology* **13**, 23 (2013).

32. Eskenazi, B.*, et al.* CHAMACOS, A Longitudinal Birth Cohort Study: Lessons from the Fields. *Journal of Children's Health* **1**, 3-27 (2003).

33. Eskenazi, B.*, et al.* Association of in utero organophosphate pesticide exposure and fetal growth and length of gestation in an agricultural population. *Environmental health perspectives* **112**, 1116-1124 (2004).

34. Yousefi, P.*, et al.* Considerations for normalization of DNA methylation data by Illumina 450K BeadChip assay in population studies. *Epigenetics* **8**, 1141-1152 (2013).

35. McConnell, R.*, et al.* Traffic, susceptibility, and childhood asthma. *Environmental health perspectives* **114**, 766-772 (2006).

36. Triche, T.J., Jr., Weisenberger, D.J., Van Den Berg, D., Laird, P.W. & Siegmund, K.D. Low-level processing of Illumina Infinium DNA Methylation BeadArrays. *Nucleic acids research* **41**, e90 (2013).

37. Touleimat, N. & Tost, J. Complete pipeline for Infinium((R)) Human Methylation 450K BeadChip data processing using subset quantile normalization for accurate DNA methylation estimation. *Epigenomics* **4**, 325-341 (2012).

38. Pritchard, J.K., Stephens, M. & Donnelly, P. Inference of population structure using multilocus genotype data. *Genetics* **155**, 945-959 (2000).

39. Heude, B.*, et al.* Cohort Profile: The EDEN mother-child cohort on the prenatal and early postnatal determinants of child health and development. *International journal of epidemiology* **45**, 353-363 (2016).

40. Gagnon-Bartsch, J.A. & Speed, T.P. Using control genes to correct for unwanted variation in microarray data. *Biostatistics (Oxford, England)* **13**, 539-552 (2012).

41. Vineis, P.*, et al.* The exposome in practice: Design of the EXPOsOMICS project. *International journal of hygiene and environmental health* **220**, 142-151 (2017).

42. Chatzi, L.*, et al.* Cohort Profile: The Mother-Child Cohort in Crete, Greece (Rhea Study). *International journal of epidemiology* **46**, 1392-1393k (2017).

43. Janssen, B.G.*, et al.* Cohort Profile: The ENVIRonmental influence ON early AGEing (ENVIRONAGE): a birth cohort study. *International journal of epidemiology* **46**, 1386-1387m (2017).

44. Farchi, S.*, et al.* Piccolipiu, a multicenter birth cohort in Italy: protocol of the study. *BMC pediatrics* **14**, 36 (2014).

45. L'Abee, C.*, et al.* Cohort Profile: the GECKO Drenthe study, overweight programming during early childhood. *International journal of epidemiology* **37**, 486-489 (2008).

46. Guillemette, L.*, et al.* Genetics of Glucose regulation in Gestation and Growth (Gen3G): a prospective prebirth cohort of mother-child pairs in Sherbrooke, Canada. *BMJ open* **6**, e010031 (2016).

47. Kooijman, M.N.*, et al.* The Generation R Study: design and cohort update 2017. *European journal of epidemiology* **31**, 1243-1264 (2016).

48. Kruithof, C.J.*, et al.* The Generation R Study: Biobank update 2015. *European journal of epidemiology* **29**, 911-927 (2014).

49. Whole-genome sequence variation, population structure and demographic history of the Dutch population. *Nature genetics* **46**, 818-825 (2014).

50. Bonder, M.J.*, et al.* Genetic and epigenetic regulation of gene expression in fetal and adult human livers. *BMC genomics* **15**, 860 (2014).

51. Paternoster, L.*, et al.* Genome-wide population-based association study of extremely overweight young adults--the GOYA study. *PloS one* **6**, e24303 (2011).

52. Nohr, E.A.*, et al.* Severe obesity in young women and reproductive health: the Danish National Birth Cohort. *PloS one* **4**, e8444 (2009).

53. Guxens, M.*, et al.* Cohort Profile: the INMA--INfancia y Medio Ambiente--(Environment and Childhood) Project. *International journal of epidemiology* **41**, 930-940 (2012).

54. Westerway, S.C., Davison, A. & Cowell, S. Ultrasonic fetal measurements: new Australian standards for the new millennium. *The Australian & New Zealand journal of obstetrics & gynaecology* **40**, 297-302 (2000).

55. van Iterson, M.*, et al.* MethylAid: visual and interactive quality control of large Illumina 450k datasets. *Bioinformatics (Oxford, England)* **30**, 3435-3437 (2014).

56. Lehne, B.*, et al.* A coherent approach for analysis of the Illumina HumanMethylation450 BeadChip improves data quality and performance in epigenome-wide association studies. *Genome biology* **16**, 37 (2015).

57. Nygaard, V., Rodland, E.A. & Hovig, E. Methods that remove batch effects while retaining group differences may lead to exaggerated confidence in downstream analyses. *Biostatistics (Oxford, England)* **17**, 29-39 (2016).

58. Arshad, S.H.*, et al.* Cohort Profile: The Isle Of Wight Whole Population Birth Cohort (IOWBC). *International journal of epidemiology* **47**, 1043-1044i (2018).

59. Arshad, S.H., Karmaus, W., Zhang, H. & Holloway, J.W. Multigenerational cohorts in patients with asthma and allergy. *The Journal of allergy and clinical immunology* **139**, 415-421 (2017).

60. Magnus, P.*, et al.* Cohort Profile Update: The Norwegian Mother and Child Cohort Study (MoBa). *International journal of epidemiology* **45**, 382-388 (2016).

61. Haberg, S.E.*, et al.* Maternal folate levels in pregnancy and asthma in children at age 3 years. *The Journal of allergy and clinical immunology* **127**, 262-264, 264.e261 (2011).

62. Joubert, B.R.*, et al.* 450K epigenome-wide scan identifies differential DNA methylation in newborns related to maternal smoking during pregnancy. *Environmental health perspectives* **120**, 1425-1431 (2012).

63. Ronningen, K.S.*, et al.* The biobank of the Norwegian Mother and Child Cohort Study: a resource for the next 100 years. *European journal of epidemiology* **21**, 619-625 (2006).

64. Jarvelin, M.R., Hartikainen-Sorri, A.L. & Rantakallio, P. Labour induction policy in hospitals of different levels of specialisation. *British journal of obstetrics and gynaecology* **100**, 310-315 (1993).

65. Jaaskelainen, A.*, et al.* Meal frequencies modify the effect of common genetic variants on body mass index in adolescents of the northern Finland birth cohort 1986. *PloS one* **8**, e73802 (2013).

66. Wijga, A.H.*, et al.* Cohort profile: the prevention and incidence of asthma and mite allergy (PIAMA) birth cohort. *International journal of epidemiology* **43**, 527-535 (2014).

67. Girchenko, P.*, et al.* Cohort Profile: Prediction and prevention of preeclampsia and intrauterine growth restriction (PREDO) study. *International journal of epidemiology* **46**, 1380-1381g (2017).

68. Morin, A.M.*, et al.* Maternal blood contamination of collected cord blood can be identified using DNA methylation at three CpGs. *Clinical epigenetics* **9**, 75 (2017).

69. Price, M.E.*, et al.* Additional annotation enhances potential for biologically-relevant analysis of the Illumina Infinium HumanMethylation450 BeadChip array. *Epigenetics & chromatin* **6**, 4 (2013).

70. Oken, E.*, et al.* Cohort profile: project viva. *International journal of epidemiology* **44**, 37-48 (2015).

71. Straker, L.*, et al.* Cohort Profile: The Western Australian Pregnancy Cohort (Raine) Study-Generation 2. *International journal of epidemiology* **46**, 1384-1385j (2017).

72. Fortin, J.P., Fertig, E. & Hansen, K. shinyMethyl: interactive quality control of Illumina 450k DNA methylation arrays in R. *F1000Research* **3**, 175 (2014).

73. Assenov, Y.*, et al.* Comprehensive analysis of DNA methylation data with RnBeads. *Nature methods* **11**, 1138-1140 (2014).

74. Kho, A.T.*, et al.* Age, Sexual Dimorphism, and Disease Associations in the Developing Human Fetal Lung Transcriptome. *American journal of respiratory cell and molecular biology* **54**, 814-821 (2016).

75. Vyhlidal, C.A.*, et al.* Cotinine in human placenta predicts induction of gene expression in fetal tissues. *Drug metabolism and disposition: the biological fate of chemicals* **41**, 305-311 (2013).
