# Supplementary material for: Epigenome-wide meta-analysis of blood DNA methylation in newborns and children identifies numerous loci related to gestational age
Source: Genome Med. 2020 Mar 2;12:25. doi: 10.1186/s13073-020-0716-9 (PMC7050134; doi:10.1186/s13073-020-0716-9)

**Figure S1.** Forest plot for the top 10 Bonferroni-significant CpGs from the meta-analysis on the association between continuous GA and offspring DNA methylation at birth adjusted for estimated cell proportions.


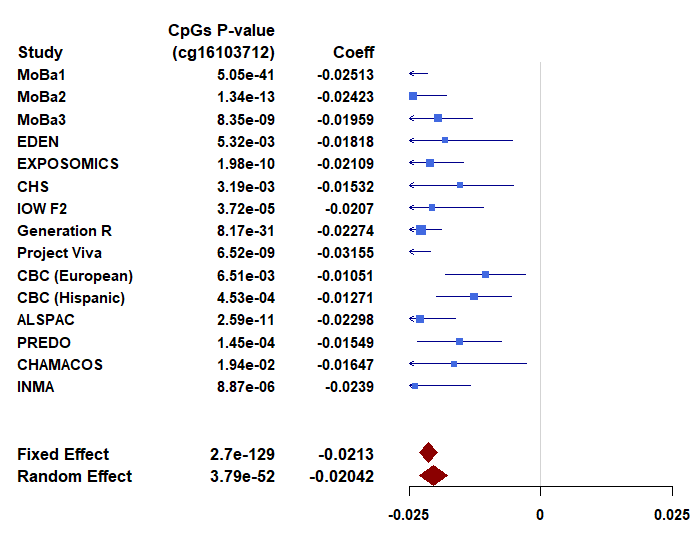

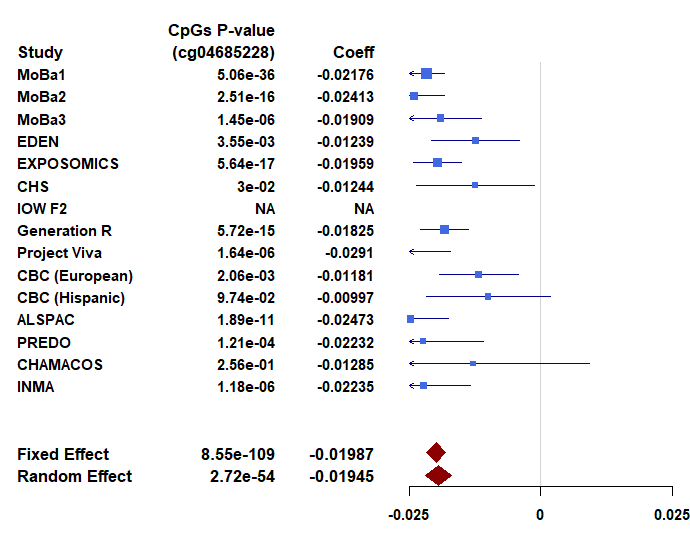


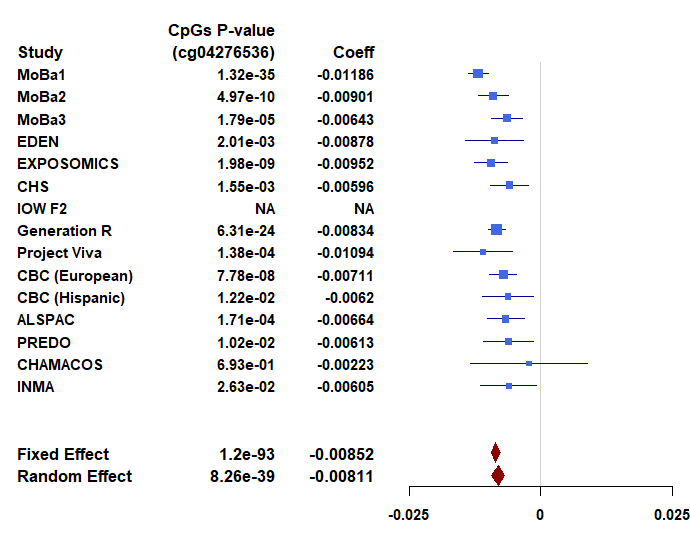

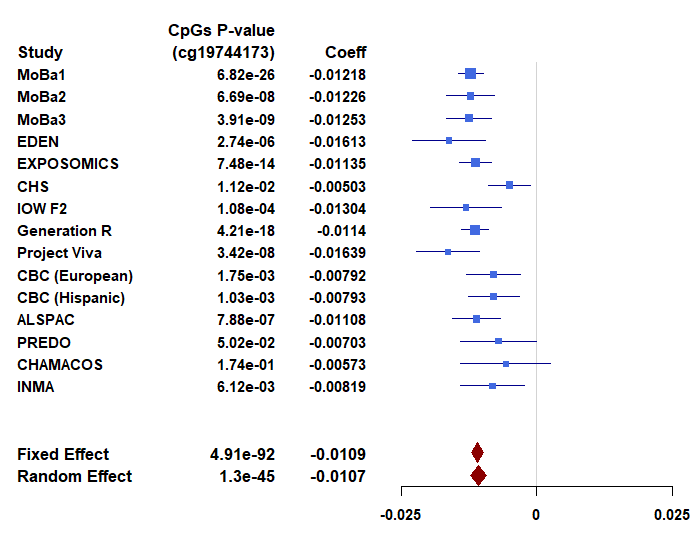


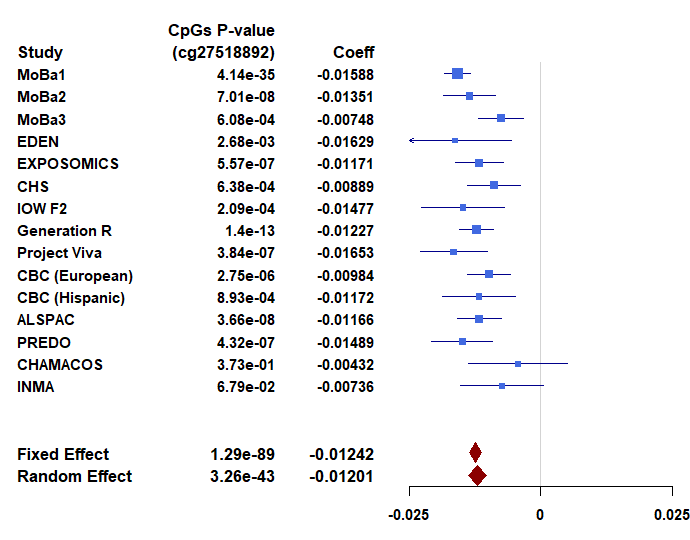

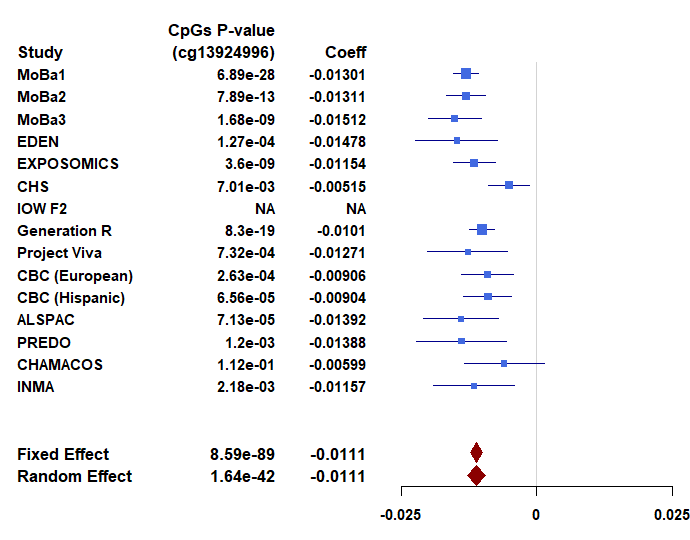


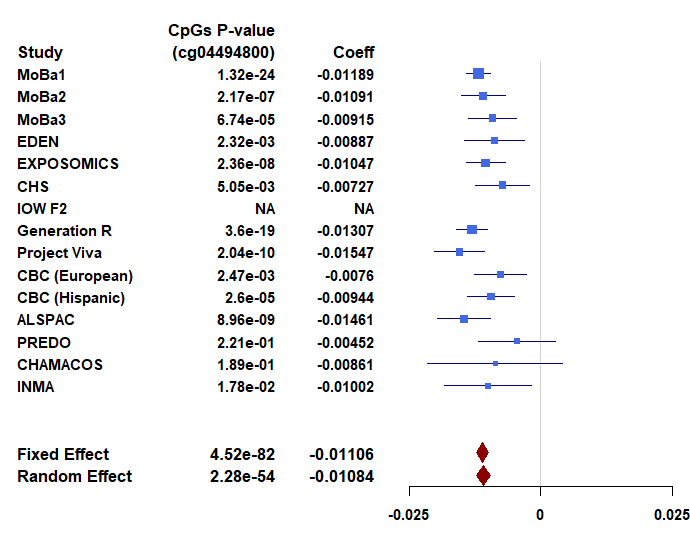

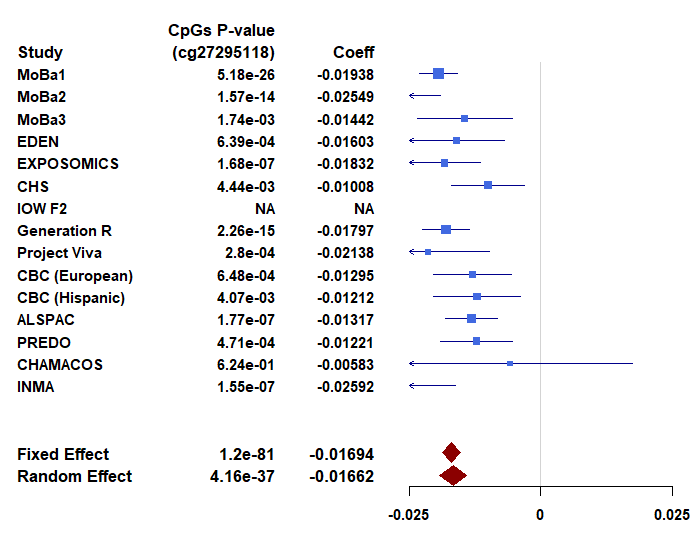


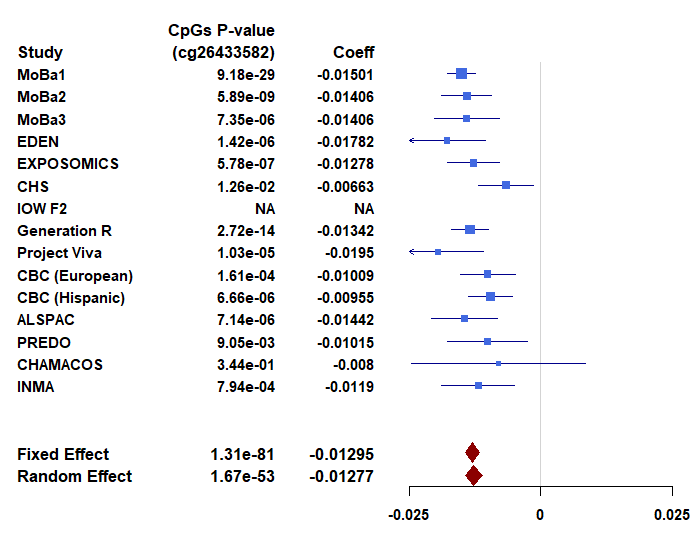

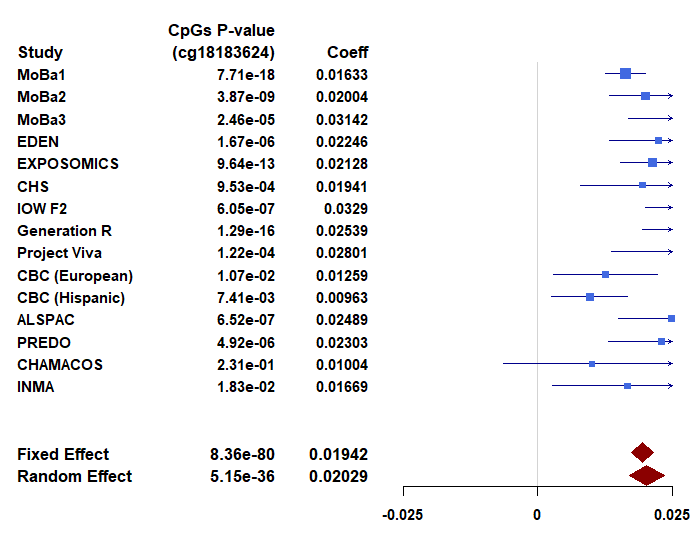


# **Figure S2.** Sensitivity analysis: Correlation of the point estimates for the no complications model main association of DNA methylation with gestational age (y-axis representing 3,648 participants from 17 cohorts) with point estimates for a meta-analysis after excluding three cohorts (MoBa1, MoBa2 and ALSPAC) that were included in a previous publication1,2 (x-axis representing 2,190 participants from 14 cohorts).


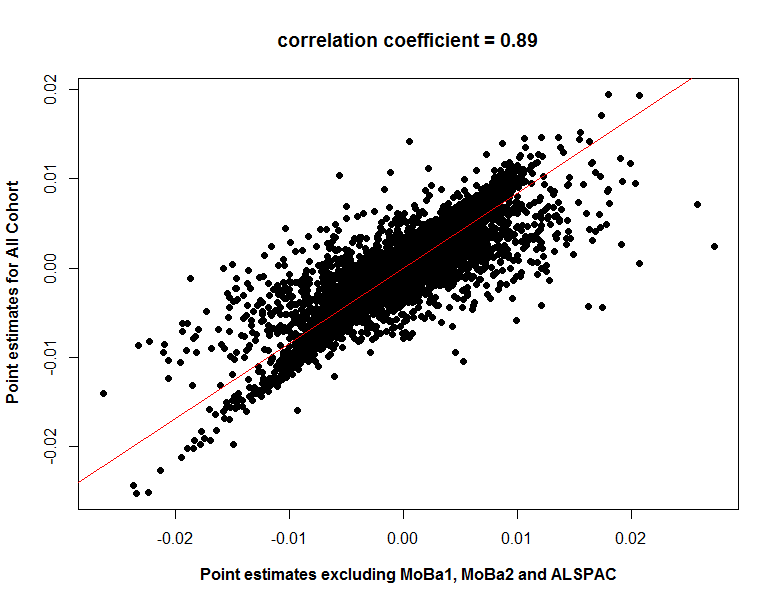


# **Figure S3.** Correlations between methylation and gene expression levels for selected four pairs. First, we created residuals for mRNA expression and residuals for DNA methylation and used linear regression models to evaluate correlations between expression residuals and methylation residuals. These residual models were adjusted for covariates, estimated white blood cell proportions, and technical variation.


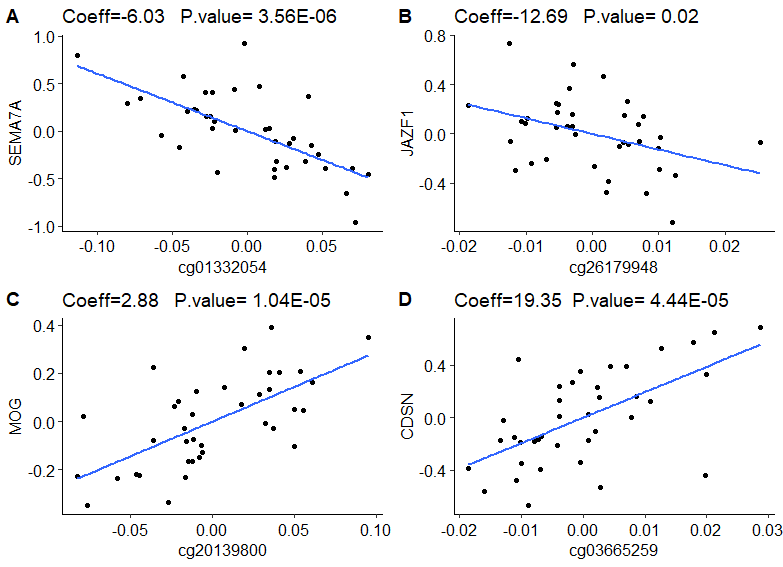


# **Figure S4.** Sensitivity analysis: Correlation of the point estimates for the no complications model main association of DNA methylation with gestational age (y-axis representing 3,648 participants from 17 cohorts) with point estimates for a meta-analysis after excluding Non-European three cohorts (CBC, CHS and CHAMACOS) (x-axis representing 3,290 participants from 14 cohorts).


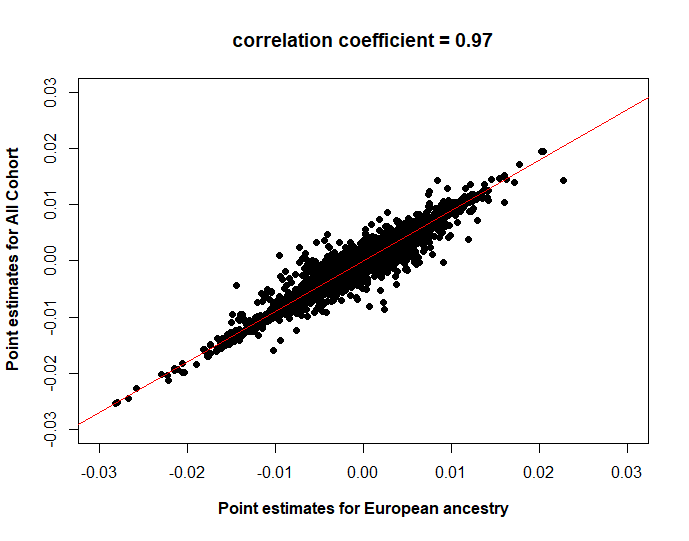

Supplement: Supplementary file 3 — Figure S1. Forest plot for the top 10 Bonferroni-significant CpGs from the meta-analysis on the association between continuous GA and offspring DNA methylation at birth adjusted for estimated cell proportions. Figure S2. Sensitivity analysis: Correlation of the point estimates for the no complications model main association of DNA methylation with gestational age (y-axis representing 3648 participants from 17 cohorts) with point estimates for a meta-analysis after excluding three cohorts (MoBa1, MoBa2 and ALSPAC) that were included in a previous publication1,2 (x-axis representing 2190 participants from 14 cohorts). Figure S3. Correlations between methylation and gene expression levels for selected four pairs. First, we created residuals for mRNA expression and residuals for DNA methylation and used linear regression models to evaluate correlations between expression residuals and methylation residuals. These residual models were adjusted for covariates, estimated white blood cell proportions, and technical variation. Figure S4. Sensitivity analysis: Correlation of the point estimates for the no complications model main association of DNA methylation with gestational age (y-axis representing 3648 participants from 17 cohorts) with point estimates for a meta-analysis after excluding Non-European three cohorts (CBC, CHS and CHAMACOS) (x-axis representing 3290 participants from 14 cohorts). [file 13073_2020_716_MOESM3_ESM.docx]
